# Design, Synthesis and Biological Characterization of Histone Deacetylase 8 (HDAC8) Proteolysis Targeting Chimeras (PROTACs) with Anti-Neuroblastoma Activity

**DOI:** 10.3390/ijms23147535

**Published:** 2022-07-07

**Authors:** Salma Darwish, Ehab Ghazy, Tino Heimburg, Daniel Herp, Patrik Zeyen, Rabia Salem-Altintas, Johannes Ridinger, Dina Robaa, Karin Schmidtkunz, Frank Erdmann, Matthias Schmidt, Christophe Romier, Manfred Jung, Ina Oehme, Wolfgang Sippl

**Affiliations:** 1 Department of Medicinal Chemistry, Institute of Pharmacy, Martin-Luther University of Halle-Wittenberg, 06120 Halle (Saale), Germany; salma.darwish@pharmazie.uni-halle.de (S.D.); ehab.ghazy@pharmazie.uni-halle.de (E.G.); tino.heimburg@web.de (T.H.); patrik.zeyen@gmail.com (P.Z.); dina.robaa@pharmazie.uni-halle.de (D.R.); frank.erdmann@pharmazie.uni-halle.de (F.E.); matthias.schmidt@pharmazie.uni-halle.de (M.S.); 2 Department of Pharmaceutical Chemistry, Faculty of Pharmacy, Alexandria University, Alexandria 21521, Egypt; 3 Institute of Pharmaceutical Sciences, University of Freiburg, 79104 Freiburg, Germany; daniel.herp@web.de (D.H.); karin.schmidtkunz@pharmazie.uni-freiburg.de (K.S.); manfred.jung@pharmazie.uni-freiburg.de (M.J.); 4 Hopp Children’s Cancer Center Heidelberg (KiTZ), Im Neuenheimer Feld 280, 69120 Heidelberg, Germany; r.altintas@kitz-heidelberg.de (R.S.-A.); j.ridinger@kitz-heidelberg.de (J.R.); i.oehme@kitz-heidelberg.de (I.O.); 5 Clinical Cooperation Unit Pediatric Oncology, German Cancer Research Center (DKFZ), 69120 Heidelberg, Germany; 6 German Cancer Consortium (DKTK), 69120 Heidelberg, Germany; 7 Faculty of Medicine, Heidelberg University, 69120 Heidelberg, Germany; 8 Département de Biologie Structurale Intégrative, Institut de Génétique et de Biologie Moléculaire et Cellulaire (IGBMC), Centre National de la Recherche Scientifique, Institut National de la Santé et de la Recherche Médicale, Université de Strasbourg, CEDEX, 67404 Illkirch, France; romier@igbmc.fr

**Keywords:** histone deacetylases (HDACs), HDAC8, proteolysis targeting chimera (PROTAC), neuroblastoma, synthesis

## Abstract

In addition to involvement in epigenetic gene regulation, histone deacetylases (HDACs) regulate multiple cellular processes through mediating the activity of non-histone protein substrates. The knockdown of HDAC8 isozyme is associated with the inhibition of cell proliferation and apoptosis enhancement in several cancer cell lines. As shown in several studies, HDAC8 can be considered a potential target in the treatment of cancer forms such as childhood neuroblastoma. The present work describes the development of proteolysis targeting chimeras (PROTACs) of HDAC8 based on substituted benzhydroxamic acids previously reported as potent and selective HDAC8 inhibitors. Within this study, we investigated the HDAC8-degrading profiles of the synthesized PROTACs and their effect on the proliferation of neuroblastoma cells. The combination of in vitro screening and cellular testing demonstrated selective HDAC8 PROTACs that show anti-neuroblastoma activity in cells.

## 1. Introduction

Reversible acetylation and deacetylation of histone tails influence gene expression. While acetylation is catalyzed by histone acetyltransferases (HATs), the removal of the acetyl mark is catalyzed by histone deacetylases (HDACs). In addition to histones, these opposing enzymes regulate many cellular processes through dynamic acetylation and deacetylation of non-histone proteins such as transcription factors (p53), nuclear import factors, and cytoskeletal proteins (α-tubulin). Abnormal acetylation/acylation of histones and non-histone proteins has been found to contribute to the development of various diseases [1,2,3].

Neuroblastoma is the most common childhood extracranial solid tumor. It emerges from precursor cells in the sympathetic nervous system, leading to the development of tumors in the adrenal glands and/or the sympathetic ganglia [4]. HDAC8 is a unique class I zinc-dependent HDAC. From all classical HDACs, only HDAC8 overexpression was significantly correlated with the advanced stage of the disease and metastasis. However, it was found to be downregulated in 4S neuroblastoma cases which are characterized by increased spontaneous incidence of regression and high survival rate despite metastasis into liver, skin, and bone marrow. In addition, the knockdown of HDAC8 in cultured neuroblastoma cells resulted in inhibition of proliferation and induction of cell cycle arrest and differentiation [5,6,7]. Consequently, selective HDAC8 inhibition or degradation is a promising therapeutic strategy in neuroblastoma.

**Figure 1:**
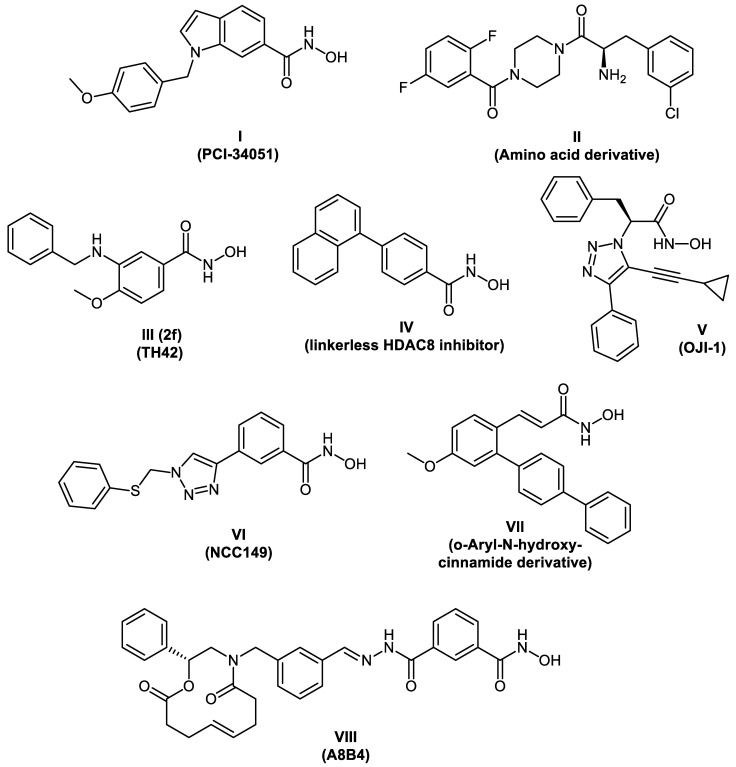
Chemical structures of reported HDAC8 inhibitors.

In recent years, several HDAC8 inhibitors have been reported (representative examples in Figure 1) [8,9,10,11,12,13,14,15,16]. In 2008, PCI-34051 I was reported as a potent and selective HDAC8 inhibitor. It shows good selectivity in vitro for HDAC8 compared to other subtypes tested (HDACs 1, 2, 3, 6, and 10) [8]. Chemotypes other than hydroxamic acids have also been reported to potently inhibit HDAC8. In the investigation made by Whitehead et al., the amino acid derivative II showed good activity against HDAC8 (IC_50_ = 0.20 μM) accompanied with a good selectivity profile against HDAC1, HDAC2, and HDAC6 [15].

In 2017, we reported the synthesis of a series of para-substituted 3-aminobenzhydroxamic acids as potent HDAC8 inhibitors. Compound III with a methoxy group in the para position exhibited strong HDAC8 inhibitory activity (IC_50_ = 0.07 μM) coupled with selectivity over both HDAC isoforms 1 and 6. In addition, Compound III showed an anti-proliferative effect in several neuroblastoma cell lines [16].

The aforementioned HDAC8 inhibitors were designed based on the occupancy pharmacology in which the inhibitor exerts its function only by occupying a well-defined active or allosteric site instead of the biological substrate. The developed small molecules have to bind to the targeted site with strong affinity. This approach is connected with the evolution of side effects and resistance [17]. On the other hand, targeted protein degradation aims to induce degradation of the targeted protein specifically through hi-jacking the cellular protein quality control machinery. It offers a new concept to chemically knock out protein targets. Recent studies report several advantages over the classical occupancy-driven approach [17,18,19].

Proteolysis targeting chimeras (PROTACs) are heterobifunctional molecules composed of a protein-targeting warhead and an E3 ligase ligand linked by a linker. This way it can bind the protein targeted for degradation and the E3 ligase simultaneously. The key to success in developing the bifunctional molecule is the right pairing of an E3 ligase recruiting ligand with a POI targeting ligand and linking both with a suitable linker. When the linker employed possesses the appropriate flexibility and length, ubiquitination of the protein of interest can take place. The labeled protein is recognized by the 26S proteasome and is degraded [20,21]. Several types of PROTACs are available based on the different properties and characteristics of the E3-ligase ligands [22,23,24,25] and the POI warheads [26,27,28,29]. 

**Figure 2:**
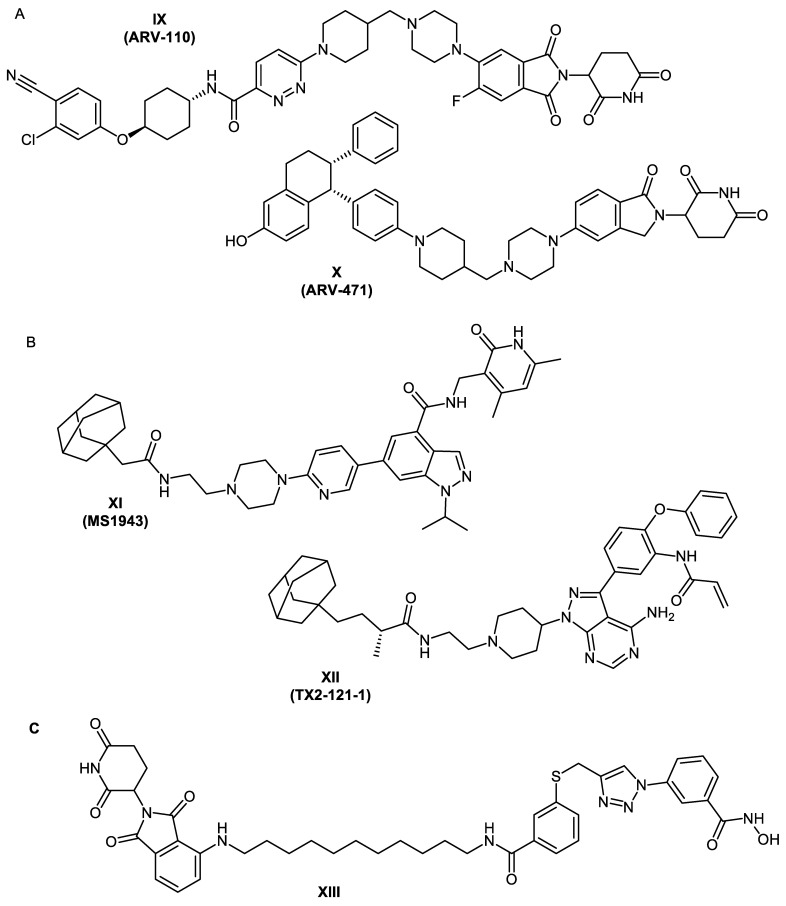
(**A**) First PROTACs to enter in-human clinical trials (https://www.guidetopharmacology.org (accessed on 1 March 2022)). (**B**) Examples of reported bifunctional hydrophobically tagged molecules. (**C**) Reported HDAC8 PROTAC.

In 2019, the first PROTACs entered human clinical trials. The orally bioavailable bifunctional molecules ARV-110 and ARV-471 (Figure 2A) target androgen receptors [30] and estrogen receptor alpha [31], respectively. As a result of their promising results and their acceptable safety and tolerability, these two PROTACs are currently in phase 2 trials [32]. These examples prove that PROTAC technology is a promising therapeutic approach.

Another approach to protein degradation is based on the attachment of hydrophobic labels to the desired inhibitors. Taking the knowledge that, in eukaryotic cells, exposed hydrophobic residues in misfolded proteins result in their degradation, Neklesa et al. and Long et al. proved that covalent [33,34] and non-covalent [35] attachment of hydrophobic groups to the POI target it for degradation by the cell’s quality control machinery. The most studied and applied hydrophobic markers are the adamantyl group [33,34] and tert-butyl carbamate-protected arginine (Boc_3_Arg) moiety [35]. Similar to a PROTAC, the bifunctional molecule employed for hydrophobic tagging (HyT) is composed of a hydrophobic group, a ligand of the POI linked together through a linker [17,33,36]. The hydrophobic label can initiate the proteasomal degradation either through destabilizing the POI [17,33,37], thereby recruiting chaperones to it or being directly recognized by the chaperones [17,38]. In both cases, the chaperones mediate the proteasomal degradation. The latter can take place in different ways that are discussed in detail in several references [33,35,37,38,39].

During the past decade, some examples of degraders utilizing the hydrophobic tagging strategy have been developed. One of these is the first-in-class enhancer of zeste homolog 2 (EZH2) selective degrader MS1943 (Figure 2B) [40]. TX2-121-1 (Figure 2B) is another bifunctional degrader which leads to partial degradation of Her3 and reduction in Her3-dependant signaling [41]. More examples are discussed in references [42,43,44].

In the past few years, increasing interest in targeting HDACs using the PROTAC technology developed. In 2018, we published the development of the first degrader of an epigenetic eraser protein which was the Sirt2 deacetylase [27]. In the same year, Yang et al. were able to synthesize the first HDAC6 PROTAC utilizing a non-selective HDAC-inhibitor [45]. After the development of this degrader, several PROTACs targeting HDAC6 enzyme were developed [46,47]. Furthermore, a first-in-class HDAC3 specific PROTAC was reported [48].

Recently, Chotitumnavee et al. [49] reported the development of an HDAC8 PROTAC. In the three synthesized degraders, a reported NCC-149 analogue [11] was used as POI ligand and pomalidomide was used as the E3 ubiquitin ligase ligand. Both warheads were connected with aliphatic linkers of three different lengths. From the synthesized degraders, Compound XIII (Figure 2C) resulted in efficient degradation of HDAC8 enzyme in T-cell leukemia Jurkat cells without affecting the levels of HDACs 1, 2, and 6.

In the present work, we aimed at the development of bifunctional molecules that potently and selectively degrade HDAC8 in neuroblastoma cells, while not affecting the activity of the other HDAC isozymes. A further focus was the analysis of the antiproliferative effects of the PROTACs in neuroblastoma cell lines. 

## 2. Results and Discussion

### 2.1. Design Concept

**Figure 3:**
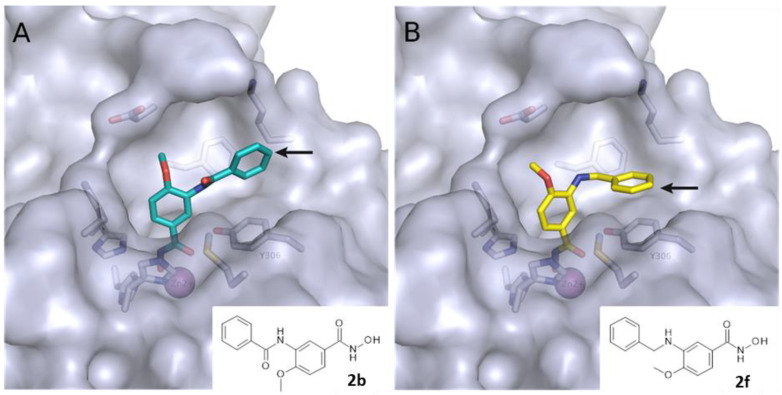
Predicted binding mode of (**A**) 2b (colored cyan) and (**B**) 2f (colored yellow) in human HDAC8 (PDB ID: 6ODB). The arrow indicates the point of linker attachment (p-position of the terminal phenyl ring).

The developed PROTACs (Table 1) were built based on previously published HDAC8 inhibitors possessing IC_50_ values in the low nanomolar range [16,50] (Table 2) In previous studies, we found that benzhydroxamates showed good HDAC8 inhibitory activity and selectivity over HDAC1 and 6. Crystallographic studies, as well as molecular docking studies on several HDAC subtypes [51], revealed that the selectivity of this series of compounds can be attributed to the fact that the aromatic capping group occupies an HDAC8-specific pocket, which is absent in the other HDAC isoforms. Based on these findings, and the fact that the aromatic capping group of the HDAC inhibitors is facing towards the exit of the binding tunnel, it can be used as an attachment point for a linker. The para-position of the phenyl capping group was chosen as an appropriate point for the attachment of the linker (Figure 3). 

**Figure 4:**
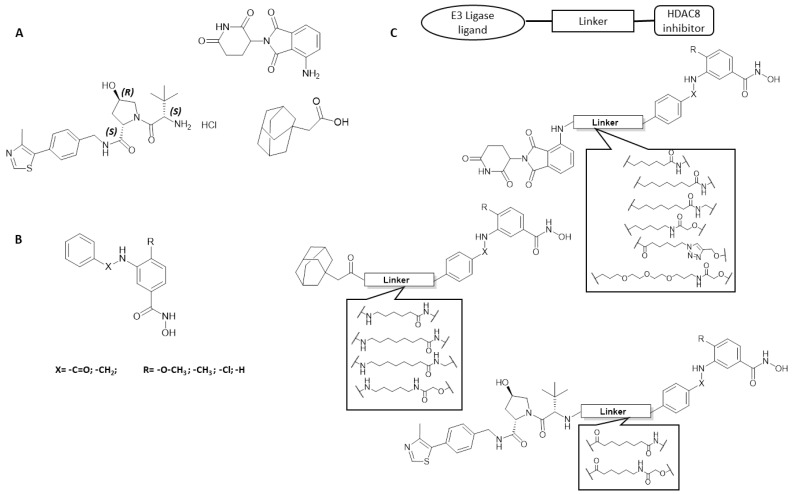
(**A**). Structure of E3 ligase ligands pomalidomide and VHL ligand as well as the adamantyl derivative HyT group. (**B**). General structure of HDAC8 inhibitors. (**C**). Schematic representation of designed HDAC8 PROTACs.

The synthesized HDAC8 degraders were designed to act through PROTAC technology or HyT (Figure 4). In several studies, it was proven that the selectivity profile of bifunctional molecules towards protein isoforms that are closely related can be influenced by many factors including the E3 ligase recruited [52,53], the length of the linker [53,54,55,56], as well as the point of linker attachment on each of the recruiting units [53,57,58]. The reason is that the formation of the ternary complex is highly affected by these factors. In a trial to increase the probability of the engagement of a ligase by the developed degraders, we used two different ligands to recruit the two E3 ligases which are most utilized in degrader development: the cereblon (CRBN) ligand pomalidomide and a VHL ligand. We also used a variety of linkers, including PEG- and hydrocarbon-based linkers, with varying lengths in addition to triazole ring-containing linkers. The in vitro activity of the synthesized compounds against human HDAC enzymes, as well as on neuroblastoma cells, were determined.

### 2.2. Chemical Synthesis of PROTACs and Inhibitors

**Scheme 1:**
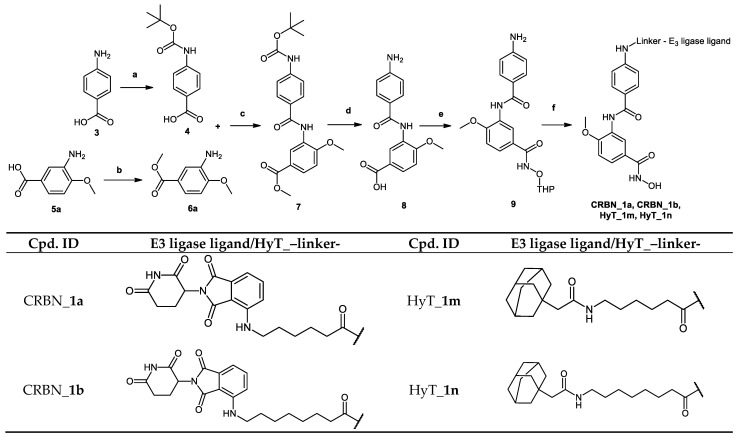
Reagents and conditions: (a) TEA, Boc_2_O, 1,4-dioxane, H_2_O, room temperature, 24 h; (b) MeOH, SOCl_2_, reflux, 3 h; (c) DIPEA, HATU, DMF, 50 °C, 24 h; (d) (1) 1 M NaOH, MeOH, reflux, 2 h; (2) TFA, DCM, room temperature, 2 h; (e) DIPEA, HATU, NH_2_OTHP, DMF, 50 °C, 24 h; (f) (1) E3 ligase ligand-linker-COOH 43a,b/HyT-linker-COOH 47a,b (see Scheme S4.1 in Supplementary Materials.), DIPEA, HATU, DMF, 50 °C, 24 h; (2) 1 M HCl, THF, US, room temperature, 2 h.

**Scheme 2:**
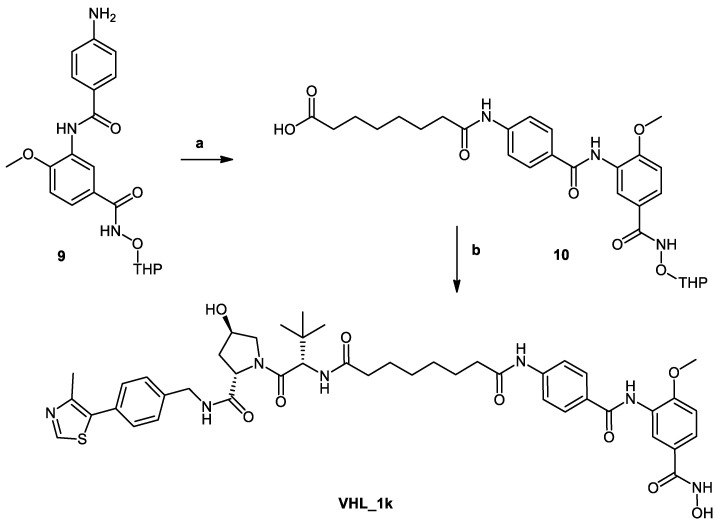
Reagents and conditions: (a) Suberic acid, NMI, TCFH, ACN, room temperature, 24 h; (b) (1) VHL ligand 40 (see S3 in Supplementary Material), DIPEA, HATU, DMF, 0 °C, 2 h; (2) 1 M HCl, THF/MeOH, US, 0 °C, 2 h.

**Scheme 3:**
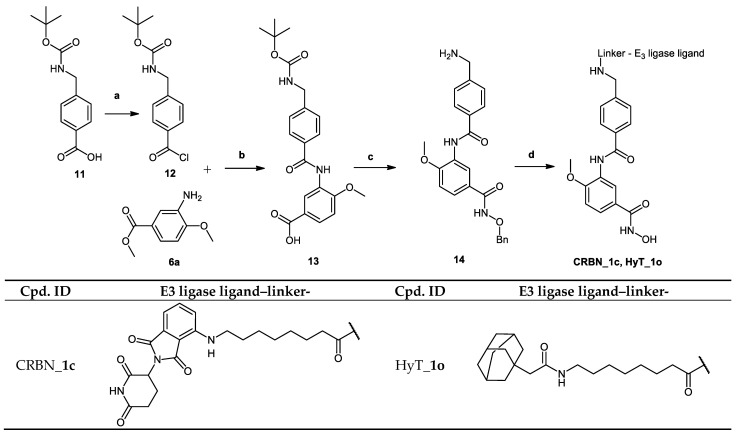
Reagents and conditions: (a) DMF, C_5_H_5_N, C_2_O_2_Cl_2_, room temperature, 6 h; (b) (1) C_5_H_5_N, room temperature, 24 h; (2) 1 M NaOH, MeOH, reflux, 2 h; (c) (1) DIPEA, HATU, NH_2_OBn·HCl, DMF, 50 °C, 24 h; (2) TFA, DCM, room temperature, 2 h; (d) (1) E3 ligase ligand-linker-COOH 43b/HyT-linker-COOH 47b (see Scheme S4.1 in Supplementary Materials), DIPEA, HATU, DMF, 50 °C, 24 h; (2) Pd/C (5%), H_2_, THF, 24 h.

**Scheme 4:**
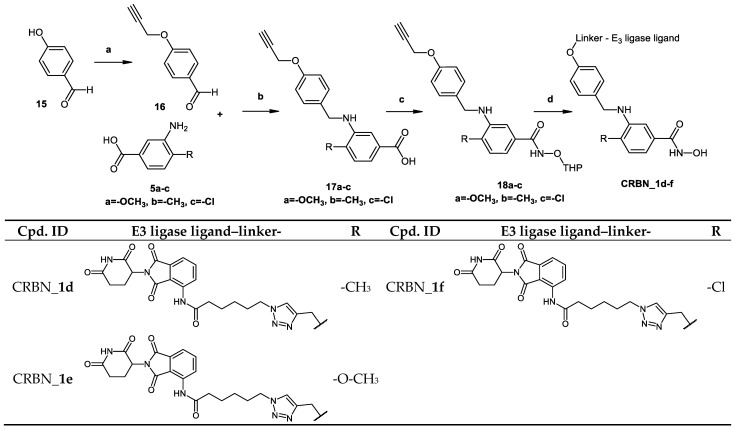
Reagents and conditions: (a) (1) K_2_CO_3_, DMF, room temperature, 1 h; (2) propargyl bromide at 0 °C; (3) 24 h at room temperature; (b) (1) toluene, reflux, 2 h; (2) THF, 0 °C, CH₃COOH, Na(CH₃COO)₃BH, 30 min; (3) 24 h, room temperature; (c) DIPEA, HATU, NH_2_OTHP, DMF, 50 °C, 24 h; (d) (1) E3 ligase ligand-linker-N_3_ 50 (see Scheme S4.2 in Supplementary Materials), THF/H_2_O, Sod. Ascorbate, CuSO_4_ × 5H_2_O, 24 h, room temperature; (2) 1 M HCl, THF, US, room temperature, 2 h.

**Scheme 5:**
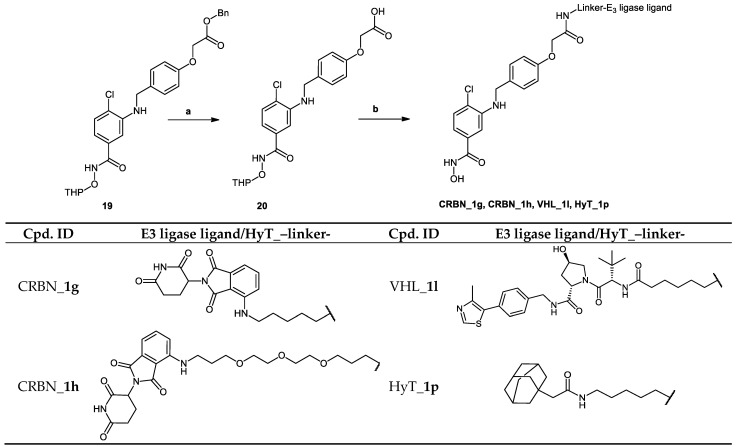
Reagents and conditions: (a) Pd/C (5%), H_2_, EtOAc: THF (1:1); (b) (1) E3 ligase ligand-linker-NH_2_ 53, 56, 59/HyT-linker-NH_2_ 57 (see Scheme S4.3 in Supplementary Materials), DIPEA, HATU, DMF, room temperature; (2) 2 M HCl, THF, room temperature.

**Scheme 6:**
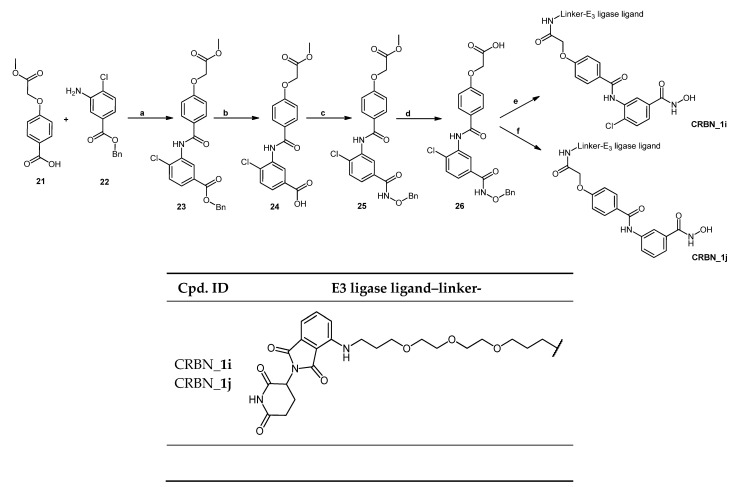
Reagents and conditions: (a) (1) SOCl_2_, reflux, (2) DIPEA, THF; (b) Pd/C (5%), H_2_, EtOAc: THF (1:1); (c) DIPEA, HATU, NH_2_OBn·HCl, DMF, room temperature; (d) LiOH, H_2_O: THF (1:1), room temperature; (e) (1) E3 ligase ligand-linker-NH_2_ 53 (see Scheme S4.3 in Supplementary Materials), HATU, DIPEA, DMF; (2) H_2_, Pd/C (5%), MeOH. (f) (1) E3 ligase ligand-linker-NH_2_ 53 (see Scheme S4.3 in Supplementary Materials), DIPEA, HATU, DMF; (2) H_2_, Pd/C (10%), MeOH.

The synthesis of the HDAC degraders (CRBN_1a-j, VHL_1k-l, HyT_1m-1p, Table 1) is summarized in Scheme 1, Scheme 2, Scheme 3, Scheme 4, Scheme 5 and Scheme 6. According to the nature of the linker, the para position of the phenyl cap group of the protected form of the hydroxamic acid-based HDAC inhibitor was functionalized. 

To synthesize the degraders (CRBN_1a–c, VHL_1k, and HyT_1m-o, Scheme 1, Scheme 2 and Scheme 3), the protected form of the hydroxamic acid based HDAC inhibitors were synthesized to contain an amino group or an aminomethyl group on the cap phenyl ring. These free amino groups were reacted with the respective carboxylic acid at the terminal part of the aliphatic linker of the E3 ligase ligand-linker-conjugates or adamantane-linker-conjugates (see Scheme S4.1 in Supplementary Materials) forming an amide bond. An exception is VHL_1k (Scheme 2) where the protected HDAC inhibitor was first linked to the linker through amidation, then the formed protected HDAC inhibitor-linker conjugate was reacted with the VHL ligand to form the protected PROTAC. Finally, the protecting group, whether benzyl or 2-tetrahydropyranyl, was removed to yield the free hydroxamic acid containing degraders.

For the synthesis of degrader molecules (CRBN_1d–f, Scheme 4) an alkyne functional group was introduced to the protected form of the hydroxamic acid-based inhibitor. On the other hand, conjugates composed of pomalidomide attached through an amide bond to an aliphatic linker terminated with an azide group were synthesized (see Scheme S4.2 in Supplementary Materials). The two units were then attached via azide-alkyne Huisgen cycloaddition, followed by deprotection of the tetrahydropyran protected hydroxamic acid to yield the bifunctional molecules. 

In the degraders (CRBN_1g–j, VHL_1l and HyT_1p) whose synthesis is demonstrated in Scheme 5 and Scheme 6, the protected form of the hydroxamic acid based HDAC inhibitor was functionalized in the para position of the cap phenyl ring with a carboxylic acid group. This group was then reacted with the amino-group in the E3 ligase ligand/HyT-linker conjugate to form an amide bond.

Scheme 6 displays the synthesis of degraders (CRBN_1i and CRBN_1j). In the first trial of the deprotection step of the benzyl protected degrader through catalytic hydrogenation, Pd/C (10%) was used. This resulted in the unwanted removal of the chlorine atom. Consequently, Pd/C (5%) was used in the subsequent deprotection trial which led to the retainment of the halogen. The unexpected degrader molecule formed was then included in the testing to investigate the effect of the absence of the para-halogen atom on the degradation profile of the degrader.

In some synthesized degraders (e.g., CRBN_1a and HyT_1m), the HDAC8 inhibitor and the linker were retained, while the entity interacting with the protein degrading machinery was changed. In other cases, both pharmacophores were kept unchanged while the length and/or nature of the linker was changed (e.g., CRBN_1a and CRBN_1b; CRBN_1g and CRBN_1h). Furthermore, as shown in Scheme 4 (CRBN_1d–f), we synthesized compounds in which only the HDAC8 inhibitor was changed through different substitution. All these designs were aimed at creating a pool of compounds for the investigation of the effect of the different factors on the degradation ability of the synthesized degraders to optimize the design of a successful HDAC8 degrader. 

The synthesis of the PROTACs (CRBN_1a, CRBN_1b, HyT_1m and HyT_1n) is elucidated in Scheme 1. After 4-((tert-butoxycarbonyl) amino)benzoic acid (4) was prepared as previously reported [14], it was reacted with methyl 3-amino-4-methoxybenzoate (6a) following Method IIIA (see Materials and Methods part) to yield methyl 3-(4-((tert-butoxycarbonyl)amino)benzamido)-4-methoxybenzoate (7). Next, the methyl ester group was hydrolyzed using Method IIA (see Materials and Methods part) and the tert-butyloxycarbonyl protecting group was removed using Method VI (see Materials and Methods part) to yield 3-(4-aminobenzamido)-4-methoxybenzoic acid (8). To complete the synthesis of the 2-tetrahydropyranyl-protected HDAC ligand (9), O-(tetrahydro-2H-pyran-2-yl) hydroxylamine was reacted with the free carboxylic acid group following Method IIIA (see Materials and Methods part). The 2-tetrahydropyranyl protected PROTACs were synthesized by reacting the different E3 ligase ligand-linker-COOH (43a,b) or HyT-linker-COOH (47a,b) (see Scheme S4.1 in Supplementary Material) with the 2-tetrahydropyranyl-protected HDAC ligand (9) as stated in Method IIIA (see Materials and Methods part). Finally, deprotection according to the Method V (see Materials and Methods part) took place to obtain the free hydroxamic acid containing PROTACs (CRBN_1a, CRBN_1b, HyT_1m and HyT_1n).

In Scheme 2, the synthesis of PROTAC (VHL_1k) is shown. First, the 2-tetrahydropyranyl-protected HDAC ligand (9) was synthesized as previously indicated in Scheme 1. Next, suberic acid was attached to the protected ligand following Method IIID to yield 8-((4-((2-methoxy-5-(((tetrahydro-2H-pyran-2-yl)oxy)carbamoyl)phenyl) carbamoyl)phenyl)amino)-8-oxooctanoic acid (10). Then the formed conjugate was reacted with the VHL ligand (40) (see S3 in Supplementary Material) using Method IIIA (see Materials and Methods part) to give the protected PROTAC which was deprotected according to Method V (see Materials and Methods part).

The synthesis of the PROTACs (CRBN_1c and HyT_1o) is shown in Scheme 3. According to Method IIIB, compound 3-(4-(((tert-butoxycarbonyl)amino) methyl)benzamido)-4-methoxybenzoic acid (13) was synthesized starting from 4-(((tert-butoxycarbonyl)amino)methyl)benzoic acid (11) and methyl 3-amino -4-methoxybenzoate (6a). Tert-butyl (4-(chlorocarbonyl)benzyl)carbamate (12) was prepared in accordance to a previously reported method [15]. The methyl ester was hydrolyzed according to Method IIA (see Materials and Methods part) to obtain the free carboxylic acid which was reacted with O-benzylhydroxylamine hydrochloride according to Method IIIA (see Materials and Methods part). Next, the tert-butyloxycarbonyl protecting group was removed using Method VI (see Materials and Methods part) to give the benzyl protected HDAC ligand (14). The PROTAC synthesis was completed by reacting the E3 ligase ligand-linker-COOH (43b) or HyT-linker-COOH (47b) (see Scheme S4.1 in Supplementary Materials) with the protected HDAC ligand (14) using Method IIIA followed by removing the benzyl group according to Method VII (see Materials and Methods part) to obtain the free hydroxamic acid. 

In Scheme 4, the synthesis of PROTACs (CRBN_1d–f) is presented. To prepare 4-substituted-3-{[4-(proparg-1-yloxy)benzyl]amino}benzoic acid (17a–c), 4-proparg-1-yl oxybenzaldehyde (16) and the corresponding amine (5a–c) were reacted according to Method IA. Afterwards, the 2-tetrahydropyranyl-protected HDAC ligands were prepared by reacting the free carboxylic acid in (17a–c) with O-(tetrahydro-2H-pyran-2-yl)hydroxylamine following Method IIIA (see Materials and Methods part). Next, these HDAC ligands (18a–c) were linked to the E3 ligase ligand–linker-N_3_ (50) (see Scheme S4.2 in Supplementary Materials) via the azide-alkyne Huisgen cycloaddition as stated in Method IV (see Materials and Methods part). Finally, the free hydroxamic acids were obtained by removing the 2-tetrahydropyranyl group following Method V (see Materials and Methods part). 

In Scheme 5, the synthesis of PROTACs (CRBN_1g, CRBN_1h, VHL_1l and HyT_1p) is shown. Benzyl 2-(4-(((2-chloro-5-(((tetrahydro-2H-pyran-2-yl)oxy)carbamoyl)phenyl) amino)methyl)phenoxy) acetate (19) was synthesized as shown in Scheme S1.2 in Supplementary Materials. Afterwards the benzyl protecting group was removed according to Method VII (see Materials and Methods part) to yield the 2-tetrahydropyranyl protected HDAC ligand (20), which was reacted with E3 ligase-linker-NH_2_ conjugates (53, 56, 59) or HyT-linker-NH_2_ (57) (see Scheme S4.3 in Supplementary Materials) via Method IIIA (see Materials and Methods part). Finally, the protected PROTACs were deprotected using Method V (see Materials and Methods part) to yield the final PROTACs.

The two starting materials 4-(2-methoxy-2-oxoethoxy)benzoic acid (21) [16] and benzyl 3-amino-4-chlorobenzoate (22) [17]—prepared as previously described—were reacted together using Method IIIC (see Materials and Methods part) to afford benzyl 4-chloro-3-[4-(2-methoxy-2-oxoethoxy)benzamido]benzoate (23). Deprotection of the benzyl ester was achieved using Method VII (see Materials and Methods part), and the resultant acid was reacted with O-benzylhydroxylamine hydrochloride using Method IIIA (see Materials and Methods part) to afford methyl 2-[4-({5-[(benzyloxy)carbamoyl]-2-chlorophenyl}carbamoyl)phenoxy]acetate (25). The ester was then hydrolyzed using Method IIB (see Materials and Methods part) to afford the benzyl protected HDAC ligand 2-[4-({5-[(benzyloxy)carbamoyl]-2-chlorophenyl}carbamoyl)phenoxy] acetic acid (26). Finally, the protected HDAC ligand was reacted with E3 ligase-linker-NH_2_ conjugate (53) (see Scheme S4.3 in Supplementary Materials) following Method IIIA (see Materials and Methods part) to yield the protected PROTAC which was deprotected to yield PROTAC (CRBN_1i) using Method VII (see Materials and Methods part). Scheme 6 shows the synthesis of the described compound.

As illustrated in Scheme 6, the use of 10% Pd/C in the catalytic hydrogenation to deprotect compound (26) resulted in the loss of the chlorine atom in the final PROTAC (CRBN_1j). That is the reason why a lower concentration of the catalyst was used. This led to the successful removal of the benzyl group while retaining the chlorine atom.

### 2.3. In Vitro Testing Using Recombinant HDACs 

In vitro testing for HDAC-inhibition (see Section 4.2. for details) using recombinant HDACs and the peptidic Fluor-de-Lys as substrate showed that the synthesized degraders having 4-substituted 3-aminobenzhydroxamates (e.g., CRBN_1d–f) as HDAC inhibitor part exhibited preference for HDAC8 over the other tested human HDACs (HDAC1 and 6) as shown in Table 1. However, degraders possessing 4-substituted 3-amidobenzhydroxamates (e.g., CRBN_1b and CRBN_1c) as a warhead showed comparable inhibitory activity against HDAC6 and 8 (Table 1). 

While CRBN-based PROTACs CRBN_1a–c were designed on the basis of the potent HDAC8 inhibitor 2b, CRBN_1d–f were synthesized based on the potent HDAC8 inhibitors 2e, 2f, 2h, and 2g respectively. The difference between the degraders CRBN_1a–c is in the length of the linker. While in CRBN_1a there is a six-carbon amide linker attached to the target binding unit, in CRBN_1b the linker is eight carbon atoms long. In the case of CRBN_1c, the unit linking the inhibitor and the E3 ligase part is elongated via the functionalization of the cap group of the inhibitor with aminomethyl group instead of amine group as in CRBN_1b. The HDAC8 inhibitory activity was comparable to the parent inhibitor in the case of CRBN_1a (SI (HDAC6/HDAC8) = 2) and CRBN_1c (SI (HDAC6/HDAC8) = 2). It decreased in the case of CRBN_1b (SI (HDAC6/HDAC8) = 0.7), but remained in the submicromolar range. On the other hand, the selectivity over HDAC6 decreased in the three PROTACs compared to 2b (SI (HDAC6/HDAC8) = 28). 

While CRBN_1a–c possess a hydrocarbon chain as a linker, CRBN_1d–f have a triazole containing linker [27]. CRBN_1d (SI (HDAC6/HDAC8) = 18) and CRBN_1e (SI (HDAC6/HDAC8) = 69) maintained a significant selectivity over HDAC6, while the selectivity of CRBN_1f over HDAC6 (SI (HDAC6/HDAC8) = 5) was found to be 3-fold lower than its parent inhibitor 2g (SI (HDAC6/HDAC8) = 16). 

The pomalidomide-based PROTACs CRBN_1g and CRBN_1h were based on the inhibitor 2e. Degraders CRBN_1g and CRBN_1h, which only differ in the type of the linker used showed an almost equipotent activity as the parent inhibitor and significant selectivity over HDAC6. The difference between degrader molecules CRBN_1i, which was designed based on inhibitor 2c, and CRBN_1j is the absence of the chloro-substituent at position-4 of the benzhydroxamic acid. CRBN_1j is the result of reductive dechlorination which took place during the synthesis of CRBN_1i. The HDAC8 activity was greatly affected and changed from the nanomolar range to the micromolar range, further confirming the importance of para-substitution for HDAC8 inhibitory activity. This observation was in accordance with our previous reports [16,50] and further confirms the importance of the para-substitution for HDAC8 inhibitory activity.

VHL_1k and CRBN_1b differ in the degradation machinery recruiting unit. While VHL_1k (SI (HDAC6/HDAC8) = 3) has a VHL_-ligand as the E3 ligase binding unit, CRBN_1b (SI (HDAC6/HDAC8) = 0.7) bears the CRBN_-warhead pomalidomide. Both degraders showed an HDAC8 inhibitory activity with the IC_50_ values in the submicromolar range. However, the selectivity over HDAC6 was lost compared to the parent inhibitor 2b (SI (HDAC6/HDAC8) = 28).

HyT_1m-p possess adamantane as the degrading machinery engaging unit. In HyT_1m-o, the linker is extended through amide formation with an acetic acid handle bound to the adamantane. In HyT_1o (SI (HDAC6/HDAC8) = 4), an elongation of the linker is achieved through the aminomethyl functionalization of the inhibitor’s cap group as compared with HyT_1n (SI (HDAC6/HDAC8) = 2). This elongation resulted in an inhibitory activity on HDAC8, comparable to the parent inhibitor 2b and was accompanied with a 2-fold increase in the selectivity over HDAC6 compared to HyT_1n. As their pomalidomide based counterparts CRBN_1a–c, HyT_1m-o demonstrated good inhibitory activity towards HDAC8 and a significant decrease in the selectivity over HDAC6 compared to the parent inhibitor 2b (SI (HDAC6/HDAC8) = 28).

Interestingly, the addition of the methylene group between the amide group and the cap group of the inhibitor in CRBN_1c and HyT_1o resulted in the improvement of the inhibitory activity against HDAC8 in comparison with CRBN_1b and HyT_1n respectively, so that the IC_50_ values of CRBN_1c and HyT_1o were similar to that determined for 2b. Although different ligands for the degrading machinery and different linkers were employed in VHL_1l and HyT_1p, both displayed equal inhibitory activity towards HDAC8 and significant selectivity over HDAC6.

Collectively, all synthesized PROTACs—with the exception of the para-unsubstituted derivative CRBN_1j—showed HDAC8 inhibitory activity in the submicromolar range, which should guarantee the ability of the bifunctional molecules to bind to HDAC8. In addition, the inhibition of HDAC1 was found to be weak. The negative control 33a (see Scheme S2.1 in Supplementary Materials) which possesses a carboxylic ester group instead of the hydroxamic acid group did not demonstrate a strong inhibitory activity against any of the tested HDACs. This confirms the necessity of the presence of a zinc binding group—in this case the hydroxamic acid group—for the degraders/inhibitors to bind to the HDAC enzymes.

All synthesized compounds and reference ligands were analytically characterized by ^1^H NMR, ^13^C NMR, MS, and HPLC purity determination. All data are shown in S1–S5 in the Supplementary Materials.

The chemical stability of two CRBN targeting PROTACs (CRBN_1b and CRBN_1e as examples) was tested under assay condition using HPLC. We used a non-enzymatic stability test in assay medium at 37 °C (see S6, Supplementary Materials) and observed that both CRBN PROTACs tested were stable at 6 h (100.0% and 91.6%) and moderately stable at 24 h (91.6% and 65.5%). This is in agreement with the reported stability of other CRBN-based degraders [20,21].

### 2.4. Cytotoxicity Assay

HDACi should have low toxicity to normal mammalian cells. To test the potential toxicity of the in vitro active PROTACs, cytotoxicity tests were performed on human embryonic kidney-derived HEK293 cells. The cells were incubated for 48 h with the PROTACs at a concentration of 50 µM, and cell viability was determined by the Alamar Blue assay. As shown in Table 3, the HDAC8 inhibitors and PROTACs showed weak to no cytotoxic effects against HEK293 cells at the used concentration of 50 µM.

### 2.5. Testing on Neuroblastoma Cells

**Figure 5:**
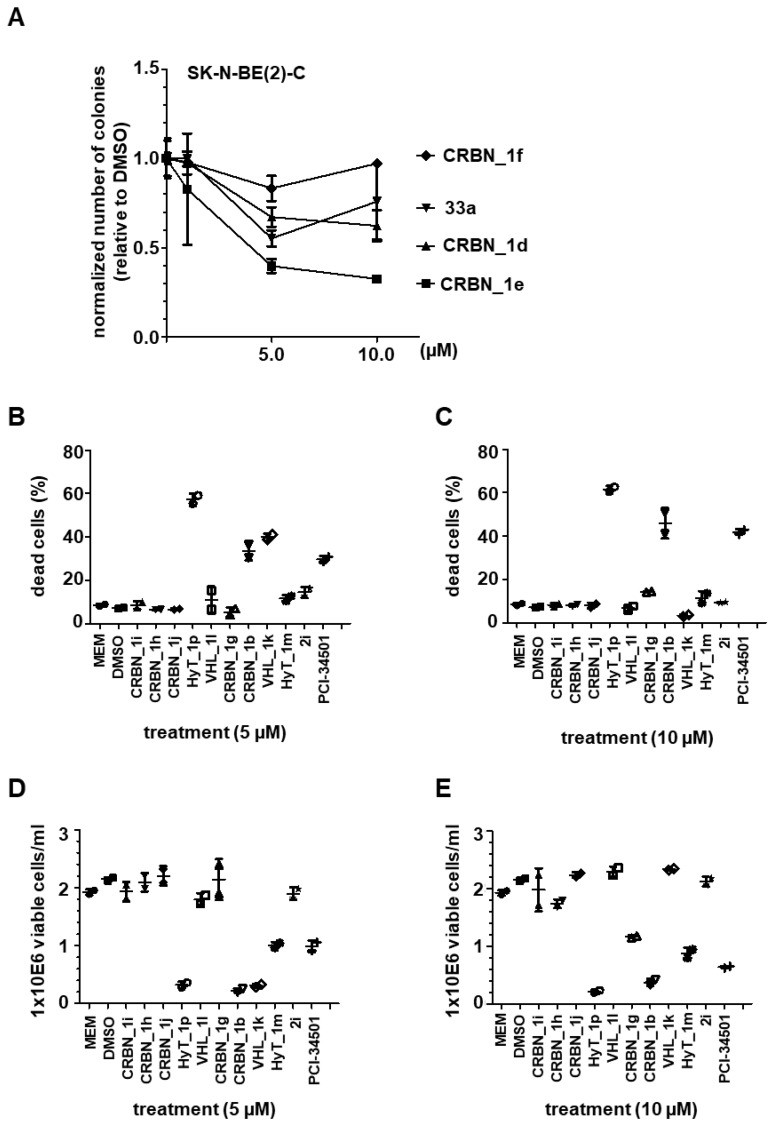
(**A**) Neuroblastoma SK-N-BE(2)-C cells; Colony Assay, 10 days (compound treatment within first 96 h). Stained with crystal violet and quantified with ImageJ. (**B**,**C**) Trypan Blue assay for detection of dead cells. Neuroblastoma IMR-32 cells were either treated with 5 µM (**B**) or 10 µM (**C**). (**D**,**E**) Cell proliferation assessed via counting of viable cells. IMR-32 cells were either treated with 5 µM (**D**) or 10 µM (**E**) PROTACs. HDAC8i PCI-34501 served as a positive control, untreated (MEM) and solvent (DMSO) treated cells served as negative controls.

In order to measure the functional consequence of HDAC8 inhibition, two different cell lines were used: SK-N-BE(2)-C cells, which display MYCN amplification and non-functional p53; and IMR-32 cells having p53 wild type, which both respond with growth arrest and signs of neuronal differentiation upon knockdown or selective inhibition of HDAC8. Growth arrest was determined by colony formation and viability assays. Cells were treated with 5 and 10 μM of each of the CRBN_ based HDAC8 PROTACs having a triazole linker (CRBN_1d, CRBN_1e, CRBN_1f) for 96 h, followed by culturing for another 6 days without treatment (Figure 5A). This assesses whether the treatment impairs the clonogenic growth capacity of tumor cells, indicating effectiveness of compounds on the survival and proliferation of tumor cells. CRBN_1d and CRBN_1e showed the strongest effect on colony formation, whereas the related analogue CRBN_1f and the negative control 33a were found to be inactive.

Moreover, we treated neuroblastoma IMR-32 cells with the remaining PROTACs and counted the resulting viable cells. We quantified the percentage of dead cells as shown in (Figure 5B,C). In addition, cell proliferation was assessed via counting of viable cells (Figure 5D,E). The CRBN_ PROTAC CRBN_1b and the HyT_ degrader HyT_1p significantly decreased the ability to form colonies at 5 and 10 µM concentrations and showed also the strongest effect in the cell proliferation assay. In case of VHL_1k and HyT_1m the results were less significant, whereas the remaining PROTACs were all found to be inactive. As reference, the HDAC8 inhibitor PCI-34501 was used [16].

**Figure 6:**
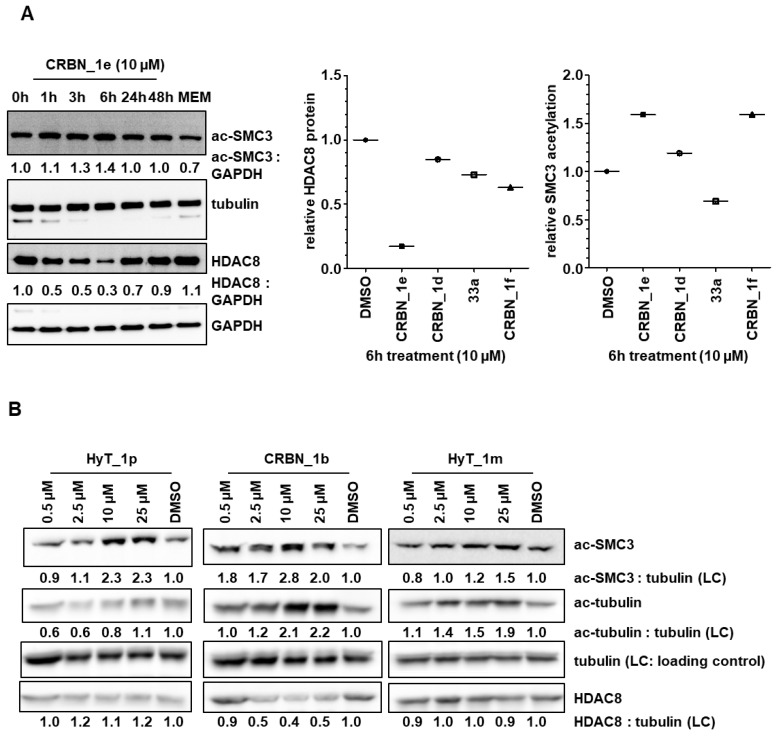

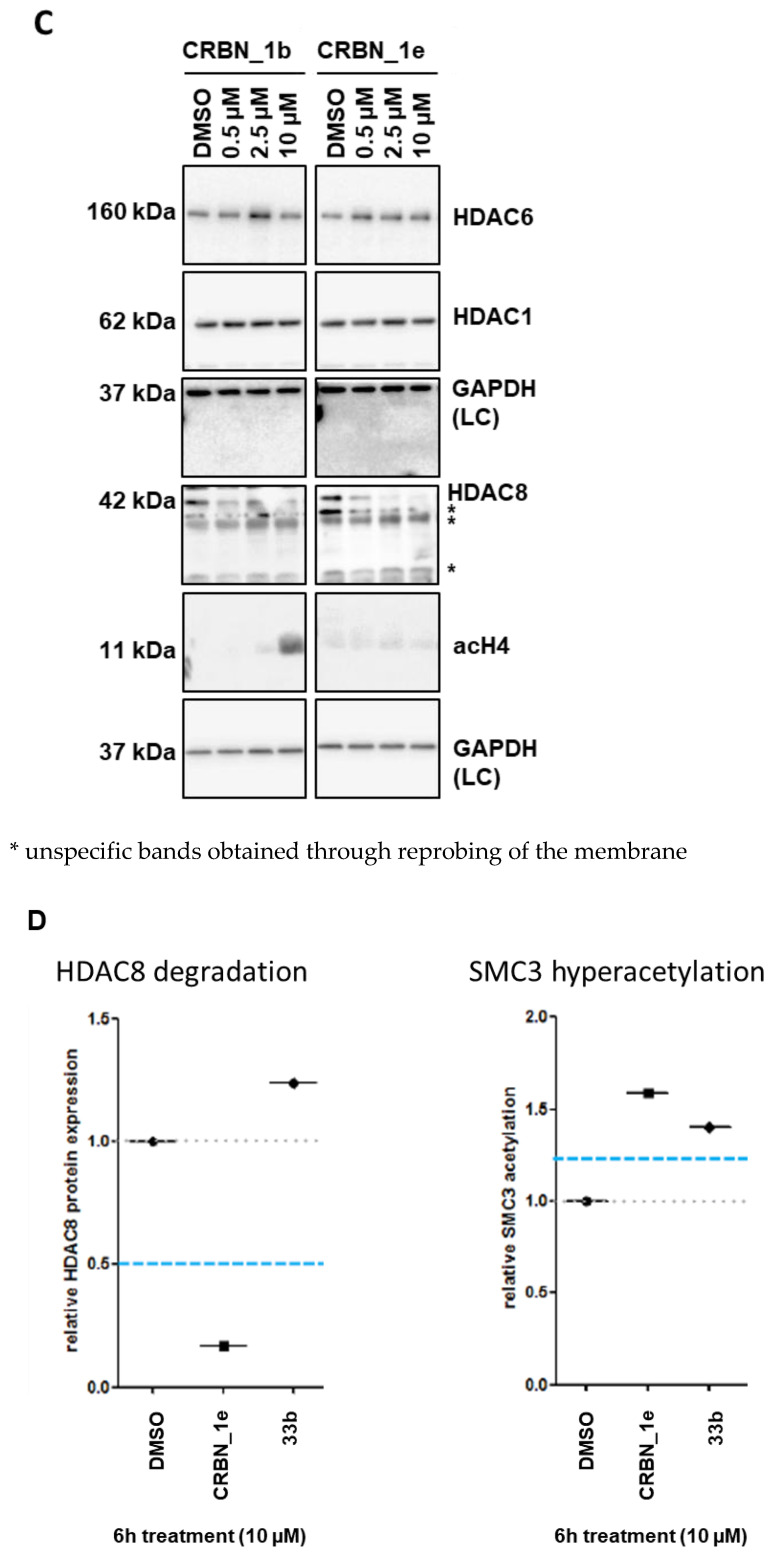
(**A**) SK-N-BE(2)-C neuroblastoma cells were treated for indicated time points with 10 µM of PROTACs. Degradation of HDAC8 and acetylation of HDAC8 target was analyzed via Western blot. (**B**) SK-N-BE(2)-C neuroblastoma cells were treated for 6 h with indicated concentrations of HyT_1p, CRBN_1b and HyT_1m. Acetylation of HDAC8 target SMC3 and HDAC6 target tubulin, as well as total HDAC8 levels, were assessed by Western blot. Quantified ac-SMC3 or ac-tubulin expression, respectively, was normalized to the respective tubulin loading control and to the solvent control (DMSO). This quantification is reflected by the numbers below each blot. (**C**) SK-N-BE(2)-C neuroblastoma cells were treated for 6 h with indicated concentrations of PROTACs CRBN_1b and CRBN_1e. Total HDAC6, total HDAC1, total HDAC8, and acetyl-histone H4 expression levels, were assessed by Western blot. Total GAPDH protein levels served as a loading control (LC). * unspecific bands obtained through reprobing of the membrane. (**D**) HDAC8 degradation and SMC3 hyperacetylation profiles of negative control 33b (HDAC8 inhibitor with an IC_50_ of 698 ± 41 nM) compared to PROTAC CRBN_1e.

To test the degradation of HDAC8 with the developed PROTACs, we selected the most promising compounds obtained from the cellular neuroblastoma testing; namely CRBN_1b, CRBN_1d, CRBN_1e, HyT_1m, and HyT_1p. Whole cell lysates from treated SK-N-BE(2)-C neuroblastoma cells were taken and the protein levels for HDAC8 and the acetylation of its substrate SMC3 were determined (Figure 6). As a control for HDAC6, we also assessed acetylation levels of α-tubulin. It revealed that 6 h treatment and a concentration of 10 µM gives the highest degradation of HDAC8 for the CRBN_ based PROTACs CRBN_1b and CRBN_1e in SK-N-BE(2)-C cells (Figure 6A–C). The CRBN_ PROTAC CRBN_1b resulted in 40% remaining HDAC8 protein whereas CRBN_ PROTAC CRBN_1e reduced HDAC8 down to 30% protein level. A clear dose-dependent degradation of HDAC8 by PROTAC CRBN_1e was observed (Figure 6C). After 48 h, the HDAC8 protein level was back to baseline, presumably due to de novo protein synthesis. To stop this effect, in another experiment the de novo protein synthesis inhibitor cycloheximide was used at low concentration (35 nM) together with the PROTAC CRBN_1e (10 μM). By inhibiting the de novo synthesis, HDAC8 degradation of CRBN_1e was still significant after 24 h, whereas cycloheximide alone showed no effect (S7, Supplementary Materials).

Both PROTACs also showed a strong hyperacetylation of the HDAC8 substrate SMC3. As expected, the negative control 33a (bearing a carboxyl ester instead of the hydroxamic acid) did not show hyperacetylation of SMC3 or HDAC8 degradation. Furthermore, the CRBN_ PROTAC CRBN_1d, and the HyT_ PROTACs HyT_1m and HyT_1p failed to degrade HDAC8 in this neuroblastoma cell line (Figure 6A,B). We also tested the most potent PROTACs CRBN_1b and CRBN_1e to see whether or not they are able to degrade HDAC1 or HDAC6 (Figure 6C and Figure S8, Supplementary Materials). PROTAC CRBN_1e showed no significant effect at the highest concentration of 10 µM. Thus, the active HDAC8 degraders CRBN_1b and CRBN_1e are selective and do not degrade HDAC1 and HDAC6. As a further negative control, we used 33b to measure HDAC8 degradation. 33b is an analog of PROTAC CRBN_1e without the pomalidomide warhead (benzyl group instead). 33b is a HDAC8 inhibitor with an IC_50_ of 698 ± 41 nM and showed hyperacetylation of SMC3 but no HDAC8 degradation when tested at 10 μM concentration (Figure 6D). Therefore, we assume that HDAC8 degradation can only be achieved by linking a CRBN ligand and a benzhydroxmate based HDAC8i as in case of CRBN_1b and CRBN_1e.

**Figure 7:**
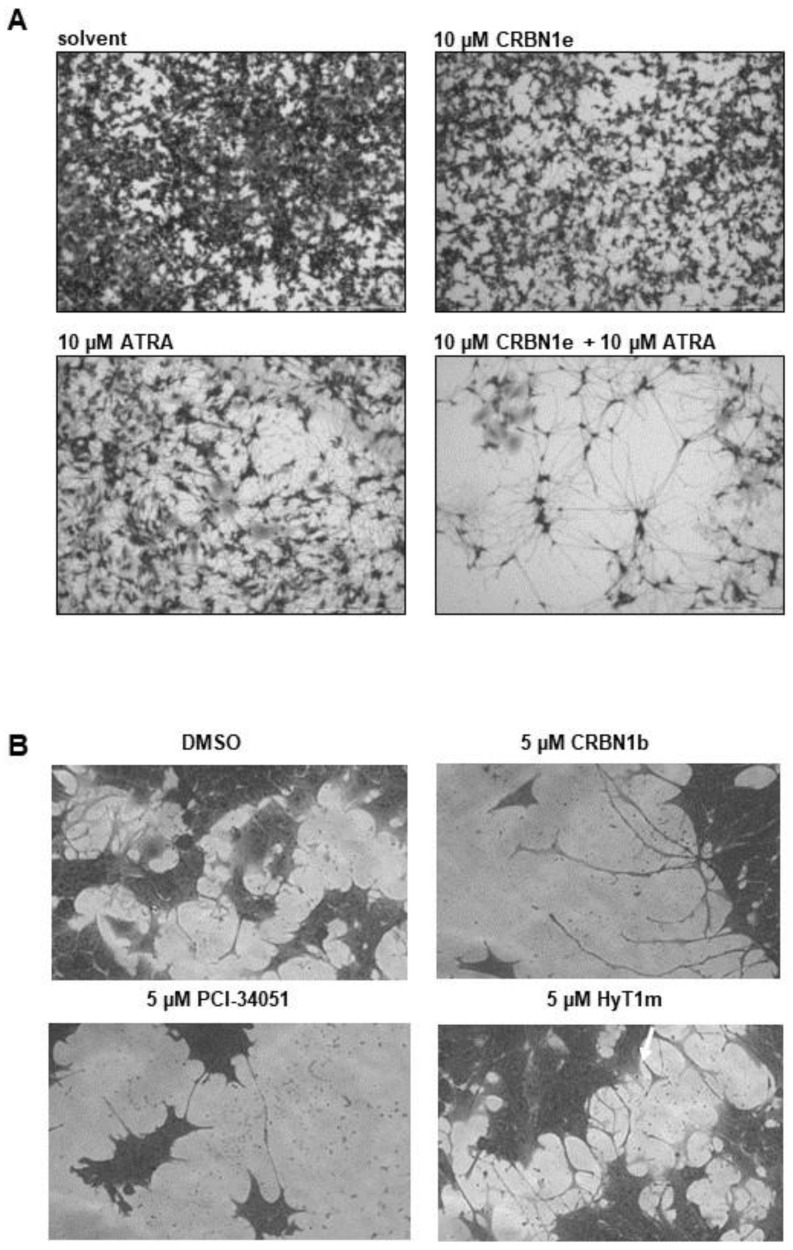
(**A**) SK-N-BE(2)-C cells, treated for 10 days. Scale bar: 500 µm. Stained with crystal violet. (**B**) SK-N-BE(2)-C cells, treated for 6 days. Stained with crystal violet.

As HDAC8 inhibition is known to induce signs of neuronal differentiation, such as neurite-like outgrowths in neuroblastoma cells [6]), we treated SK-N-BE(2)-C cells with CRBN_1b, CRBN_1e, HyT_1m, and PCI-34051 for 6–10 days then stained the cells with crystal violet to visualize neurite-like outgrowths. For comparison, we treated the cells with the known neuronal differentiation inducer retinoic acid (ATRA) which is a known drug, that is applied for neuroblastoma treatment under some circumstances. We also combined one PROTAC—namely, CRBN_1e—with ATRA, which substantially enhanced the differentiation phenotype (Figure 7). These results are in line with the published differentiation enhancement effect (longer outgrowth, more cells with outgrowth in combination) of HDAC8 inhibitors such as PCI-34051 [6].

## 3. Conclusions

In summary, we designed a pool of bifunctional PROTACs based on previously published HDAC8 inhibitors with good inhibitory activity. Different linker types and lengths in addition to various degradation machinery recruiting units were employed. The effect of these factors on the degradation ability of the synthesized PROTACs was demonstrated through testing them on SK-N-BE(2)-C neuroblastoma cells and determination of the protein levels for HDAC8 and the acetylation level of its substrate, SMC3. From the synthesized compounds only the CRBN_ based PROTACs CRBN_1b and CRBN_1e resulted in strong HDAC8 degradation connected with SMC3 acetylation. The synthesized VHL_ and HyT_ based PROTACs did not show significant HDAC8 degradation. Testing of the active PROTACs CRBN_1b and CRBN_1e against HDAC1 and HDAC6 showed no degradation, confirming the good selectivity of these compounds. Besides the good HDAC8 degradation effect of CRBN_1e, the PROTAC also exhibited good anti-neuroblastoma activity and showed enhancing of the differentiation phenotype. Overall, the developed PROTACs represent useful tools to investigate the physiological functions of HDAC8 in other cancer cells in future studies.

## 4. Materials and Methods

### 4.1. Chemistry 

#### 4.1.1. General

All materials and reagents were purchased from Sigma-Aldrich Co. Ltd (St. Louis, MI, USA) and abcr GmbH (Karlsruhe, Germany). All solvents were analytically pure and were dried before use. Thin layer chromatography was carried out on aluminum sheets coated with silica gel 60 F254 (Merck, Darmstadt, Germany). For medium pressure chromatography (MPLC) silica gel Biotage^®^ (Biotage, Uppsala, Sweden) SNAP ultra-HP-sphere 25 µm containing columns were used.

Chloroform:methanol, n-hexane:ethyl acetate, or ethyl acetate:acetonitrile were the elution systems used for medium pressure chromatography. Triethyl amine was added in a concentration of 0.1% to chloroform or ethyl acetate, according to the solvent system used, in purification of compounds protected with 2-tetrahydropyranyl group.

In the preparative high-pressure chromatography used for cleaning of the final PROTACs, LiChrosorb^®^ RP-18 (7 µm) 250-25 Merck (Merck, Darmstadt, Germany) column was used. The applied mobile phase was a gradient with increasing polarity composed of acetonitrile/water.

Final compounds’ purities were determined using high-pressure chromatography (HPLC). Purity was measured by UV absorbance at 254 nm. Two analytical methods were used while determining the purity. In the first method (M1), the components of the HPLC were an XTerra RP18 column (3.5 mm, 3.9 mm × 100 mm) from the manufacturer Waters (Milford, MA, USA) and two LC-10AD pumps, a SPD-M10A VP PDA detector, and a SIL-HT autosampler, all from the manufacturer Shimadzu (Kyoto, Japan). In the second method (M2), only the column was changed to LiChrosorb^®^ RP-18 (5 µm) 100-4.6 Merck column (Merck, Darmstadt, Germany).

Mass spectrometry analyses were performed with a Finnigan MAT710C (Thermo Separation Products, San Jose, CA, USA) for the ESIMS spectra and with an LTQ (linear ion trap) Orbitrap XL hybrid mass spectrometer (Thermo Fisher Scientific, Bremen, Germany) for the HRMS-ESI (high resolution mass spectrometry) spectra. For the HRMS analyses, the signal for the isotopes with the highest prevalence was given and calculated (35Cl, 79Br). 

^1^H NMR and ^13^C NMR spectra were taken on a Varian Inova 500 using deuterated chloroform or deuterated dimethyl sulfoxide as solvent. Chemical shifts are referenced to the residual solvent signals. 

Non-enzymatic stability of selected final compounds was determined using 10 µM concentration of the tested PROTACs in one of the following assay media; Dulbecco’s modified Eagle medium (DMEM) (50%)/dimethylsulfoxid (10%)/acetonitrile (40%) or DMEM (50%)/dimethylsulfoxid (10%)/methanol (40%) mixture at pH7.4. The formed solution mixtures were incubated for 0, 6, 12, and 24 h at 37 °C. Analyte decomposition was monitored by HPLC using XTerra RP18 column (3.5 mm, 3.9 mm × 100 mm) from the manufacturer Waters (Milford, MA, USA) and two LC-10AD pumps, a SPD-M10A VP PDA detector, and a SIL-HT autosampler, all from the manufacturer Shimadzu (Kyoto, Japan).

#### 4.1.2. General Synthetic Methods

Method I, reductive amination

- A mixture of the benzaldehyde (1 eq.) and the amine (5% molar excess) was dissolved in toluene and was heated under reflux using a water trap for 2 h. Afterwards, the solvent was removed under reduced pressure. The remaining residue was dissolved in dry tetrahydrofuran and the formed solution was cooled to 0 °C. Glacial acetic acid (2 eq.) was added followed by sodium triacetoxyborohydride (4 eq.) and the reaction mixture was stirred for 30 min at 0 °C. Afterwards, the ice bath was removed and stirring was continued for 24 h at room temperature. The reaction was then quenched by the addition of sodium bicarbonate and the product was extracted with ethyl acetate. The organic layer was washed with 1 M hydrochloric acid followed by brine and was dried over anhydrous sodium sulfate. Finally, it was filtered and evaporated under reduced pressure. The crude product was purified using the MPLC. The yields were in the 60–95% range.
- A mixture of benzaldehyde (1.1 eq.), the corresponding amine (1 eq.), trifluoroacetic acid (2 eq.), and sodium triacetoxyborohydride (1.2 eq.) was dissolved in a mixture of tetrahydrofuran and ethyl acetate (1:1). After stirring the reaction mixture at room temperature for 2 h, the reaction was quenched by adding water and the crude product was extracted with ethyl acetate. The combined organic layer was dried over anhydrous sodium sulfate, filtered, and concentrated in vacuo. The crude residue was purified using MPLC. The yield was around 50%.

Method II, ester hydrolysis

- To a solution of the methyl ester (1 eq.) in methanol, 1 M aqueous sodium hydroxide (10 eq.) was added. The formed reaction mixture was refluxed for 2–4 h. After complete ester hydrolysis, the solvent was evaporated under reduced pressure to yield a crude residue that was dissolved in water. The aqueous solution was extracted using ethyl acetate to remove any organic impurities. In the next step, 1 M aqueous hydrochloric acid (10 eq.) was added to the aqueous solution to liberate the free acid which was extracted using ethyl acetate. The combined organic layer was washed with brine and dried over anhydrous sodium sulfate. It was then filtered, and the solvent was evaporated under reduced pressure to give the crude product which was purified using the MPLC. The yields were 70–96%.
- To the suspension of the methyl ester (1 eq.) in a mixture of tetrahydrofuran and water (1:1), lithium hydroxide (5 eq.) was added. The mixture was stirred at room temperature until complete hydrolysis of the ester then tetrahydrofuran was evaporated. Using aqueous 1 M hydrochloric acid, the pH of the remaining aqueous solution was adjusted to pH 6. The liberated free acid was extracted using a mixture of ethyl acetate and tetrahydrofuran. The combined organic layer was then dried over anhydrous sodium sulfate, filtered, and concentrated in vacuo to yield the product, which required no further purification. Crude yields were around 80–90%.

Method III, amide bond formation

- A solution of the carboxylic acid (1–1.2 eq.) and N,N-diisopropylethylamine (3 eq.) in dimethylformamide was stirred for 15 min at room temperature then O-(7-Azabenzotriazol-1-yl)-N,N,N′,N′-tetramethyluronium hexafluorophosphate (1.2–1.5 eq.) was added and stirring was continued for another 30 min. Next, the corresponding amine (1–1.5 eq.) was added to the solution. The formed reaction mixture was stirred at 0 °C or at room temperature or at 50 °C for 2–24 h. After completion of the reaction, water was added to the reaction mixture and the formed solution was extracted using ethyl acetate. The combined organic layer was washed with aqueous 1 M sodium bicarbonate solution followed by aqueous 1 M ammonium chloride solution and brine. After drying over anhydrous sodium sulfate, the organic layer was filtered then concentrated in vacuo to yield the crude compound which was purified using MPLC. The yields were around 27–100%.
- To a suspension of the carboxylic acid (1 eq.) in toluene, drops of dimethylformamide were added followed by pyridine then oxalyl chloride (2 eq.). The reaction mixture was stirred at room temperature for 6 h. The formed precipitate was then filtered and washed with toluene. Afterwards, the combined organic filtrates were concentrated under reduced pressure to give the acid chloride that was used directly without further purification. It was dissolved in pyridine and the amine (1 eq.) was added to the solution. The formed reaction mixture was stirred at room temperature for 24 h. After evaporation of the solvent the remaining residue was dissolved in chloroform and was successively washed with 10% hydrochloric acid, 1 M sodium bicarbonate, and brine. After drying the organic layer over anhydrous sodium sulfate, it was evaporated under reduced pressure to give the crude product which was purified using the MPLC. The yield was around 48%.
- After the dropwise addition of thionyl chloride (3 eq.) to the carboxylic acid (1 eq.) at 0 °C, the reaction mixture was heated under reflux for 2 h then the excess thionyl chloride was evaporated under vacuum. The formed acid chloride was dissolved in dry tetrahydrofuran and was added dropwise to a solution of the corresponding amine (0.9 eq.) and N,N-diisopropylethylamine (3 eq.) in tetrahydrofuran. The reaction mixture was stirred at room temperature until completion. Afterwards, it was diluted with ethyl acetate and was washed with a saturated aqueous solution of ammonium chloride followed by brine. Finally, the organic layer was dried over anhydrous sodium sulfate, filtered, and concentrated in vacuo to obtain the crude residue which was purified using MPLC. The yield was around 50–70%.
- A mixture of the carboxylic acid (3 eq.), N-methylimidazole (3.5 eq.), and chloro-N,N,N′,N′-tetramethylformamidinium-hexafluorophosphate (1.2 eq.) were stirred in acetonitrile for 15 min. The amine (1 eq.) was dissolved in some acetonitrile, then was added to the mixture. The formed reaction mixture was stirred at room temperature for 24 h. After completion of the reaction was confirmed by TLC, water was added, and the mixture was extracted using ethyl acetate. The combined organic layer was washed with water followed by brine. After drying over anhydrous sodium sulfate, the organic layer was filtered then concentrated in vacuo to yield the crude compound which was purified using MPLC. The yield was around 67%.

Method IV, azide-alkyne Huisgen cycloaddition

In a two-necked flask, a mixture of azide containing conjugate (1 eq.), propagyl group containing ligand (1 eq.), sodium ascorbate (0.2 eq.), and copper(II) sulfate pentahydrate (0.2 eq.) were dissolved in a solvent mixture composed of tetrahydrofuran and water (2:1). After purging the reaction mixture with argon, the flask was placed in the dark and the mixture was stirred for 24 h at room temperature. After the completion of the reaction, the solvent was evaporated under reduced pressure. The remaining residue was dissolved in 1 M aqueous ammonium chloride, and the formed aqueous solution was extracted with ethyl acetate. The combined organic layer was washed with brine then it was dried over anhydrous sodium sulfate. Finally, the solvent was evaporated under reduced pressure to yield the crude product which was purified using the MPLC. The yield was around 67–70%.

Method V, deprotection of tetrahydropyranyl ether

To a solution of the 2-tetrahydropyranyl-protected product (1 eq.) in tetrahydrofuran or tetrahydrofuran with few drops of methanol, 10–15 drops of 1 M hydrochloric acid were added, and the reaction mixture was sonicated at 0 °C or room temperature for 2 h or until TLC showed completion of the reaction. The solvent was then evaporated under vacuum and the crude product was purified by preparative HPLC. The yields were around 20–50%.

Method VI, deprotection of tert-butyl protected carbamates and tert-butyl ester protected carboxylic acids

To a solution of tert-butyl protected carbamate (1 mmol) or tert-butyl ester protected carboxylic acid (1 mmol) in dichloromethane, trifluoroacetic acid (1 mL) was added. The reaction mixture was then stirred at room temperature for 2–24 h. After completion of the reaction, the solvent and excess trifluoroacetic acid were evaporated under reduced pressure to yield the crude product which was purified using the MPLC. The yields were around 78–100%.

Method VII, catalytic hydrogenation

A mixture of the benzyl-protected starting material (1 eq.) was dissolved in tetrahydrofuran or ethyl acetate or methanol or a mixture of tetrahydrofuran and ethyl acetate (1:1), then a catalytic amount of 5% Pd/C catalyst was added. The reaction mixture was put under vacuum followed by hydrogen atmosphere. The mixture was stirred at room temperature until complete consumption of the starting material. The mixture was then filtered through celite, and the solvent was evaporated to give the crude residue which was purified using MPLC. The yields were around 25–90%.

### 4.2. In Vitro HDAC Inhibitory Activity Assay

The in vitro testing on recombinant HDACs was performed as previously described [59]. Recombinant human HDAC1 and −6 were purchased from BPS Biosciences. The enzyme inhibition was determined by using a reported homogenous fluorescence assay [60]. The enzymes were incubated for 90 min at 37 °C, with the fluorogenic substrate ZMAL (Z-(Ac)Lys-AMC) in a concentration of 10.5 mM and increasing concentrations of inhibitors with subsequent addition of 60 mL of buffer containing trypsin (1 mg/mL) and TSA (2.75 mM) and further incubation for 20 min at 37 °C. Fluorescence intensity was measured at an excitation wavelength of 390 nm and an emission wavelength of 460 nm in a microtiter plate reader (BMG Polarstar).

Recombinant hHDAC8 was produced by Romier et al. in Strasbourg [61]. The HDAC8 activity assays were performed according to the commercial HDAC8 Fluorometric Drug Discovery Kit (Fluor de Lys(R)-HDAC8, BML-KI178) corresponding to the instructions of the manufacturer. As substrate a tetrapeptide connected to aminomethylcoumarin (AMC) H2N-Arg- His-Lys(Ac)-Lys(Ac)-AMC was synthesized as previously described [59]. The enzyme was incubated for 90 min at 37 °C, with a substrate concentration of 50 µM and increasing concentrations of inhibitors. The stop-solution-containing inhibitor was added to stop the hHDAC8 activity, and Trypsin was added to release the AMC. The solution was incubated for 20 min at 37 °C to develop the assay. Fluorescence intensity was measured at an excitation wavelength of 355 nm and an emission wavelength of 460 nm in a microtiter plate reader (BMG Polarstar).

Inhibition was measured at increasing concentration and IC_50_ was calculated by nonlinear regression with Origin 9.0G software.

### 4.3. Cellular Assay

- Cell Culture

Human neuroblastoma cell lines SK-N-BE(2)-C (European Collection of Authenticated Cell Cultures, ECACC, Salisbury, UK) and IMR-32 (German Collection of Microorganisms and Cell Cultures, DSMZ, Darmstadt, Germany) were cultured under standard conditions in Dulbecco’s modified Eagle medium (DMEM containing L-glutamine and 4.5 g/L glucose, Gibco Invitrogen cell culture, Invitrogen, Paisley, UK) supplemented with 10% fetal calf serum (FCS; Sigma, St. Louis, MO, USA) and 1% non-essential amino acids (NEAA; Invitrogen, Carlsbad, CA, USA). All cell lines were regularly checked for mycoplasma and multiple contaminations (Multiplexion, Heidelberg, Germany) and routinely verified using DNA fingerprinting authentication by Multiplexion.

B. Western blot

Western blot analysis was performed as described previously (Kolbinger et al.). The following antibodies were used: anti-HDAC8 (H-145) (polyclonal; Santa Cruz, Santa Cruz, CA, USA), anti-HDAC6 (sc-11420, Santa Cruz), anti-HDAC10 (H3413, Sigma-Aldrich, St. Louis, USA), anti-tubulin (#2148, Cell Signaling Technology), anti-acetylated tubulin (#6793, Sigma-Aldrich, St. Louis, USA), anti-acetylated SMC3 (kindly provided by Katsuhiko Shirahige, Institute for Molecular and Cellular Biosciences, University of Tokyo, Japan (Nishiyama et al. 2010)), anti-GAPDH (clone 6C5; Merck, Darmstad, Germany), and anti-β-actin (#5441, Sigma-Aldrich, St. Louis, USA).

C. Cell viability assay (Trypan blue assay)

Adherent cells were detached using trypsin–EDTA (ThermoFisher Scientific, Bremen, Germany) and pooled with corresponding supernatant, centrifuged, and resuspended in 1 mL of cell culture medium. Cell viability (viable cell number, % viability, % dead cells) was measured by automated trypan blue staining using the Vi-Cell XR Cell Viability Analyzer (Beckman Coulter, Krefeld, Germany).

D. Colony formation assay

In six-well plates, 500 cells were seeded and treated as indicated. Viable colonies were stained after a minimum of 10 days with crystal violet. For quantification, the mean intensity of each well of the 8-bit binary picture was measured with ImageJ software (U. S. National Institutes of Health, Bethesda, MD, USA; http://imagej.nih.gov/ij/ (accessed on 1 March 2022)).

E. Cell differentiation assay

Adherent cells plated on 6-well plates were treated as indicated. For staining, cells were rinsed once with (PBS) and incubated with crystal violet staining solution (1% (w/v) in 70% EtOH) for 1 min. Subsequently, the staining solution was removed and cells were rinsed two to three times with autoclaved purified water and allowed to dry. All-trans retinoic acid (ATRA, Sigma-Aldrich, stock concentration 10 mM) was dissolved in ethanol (EtOH, Sigma-Aldrich, St. Louis, USA).

F. Cytotoxicity Studies

HEK293 cells (DSMZ Braunschweig, ACC305) were incubated at 37 °C in a humidified incubator with 5% CO2 in Dulbecco’s modified Eagle medium (DMEM) supplemented with 10% FCS and 5 mM glutamine. The cells were seeded out at 1.5 × 103 cells per well in a 96-well cell culture plate (TPP, Techno Plastic Products AG, Trasadingen, Switzerland). All tested compounds were added immediately to the medium at 50 μM or increasing concentrations to determine IC_50_ values. After 24 h, Alamar Blue reagent (Invitrogen, Carlsbad, CA, USA) was added according to the manufacturer’s instructions and incubated again for 21 h before the samples were analyzed. Detection of the viable cells which convert the resazurine of reagent into the high fluorescent resorufin was performed by using a FLUOstar OPTIMA microplate reader (BMG Labtec, Ortenberg, Germany) with the following filter set: Ex 560 nm/Em 590 nm.

The measurements were performed in triplicate, and data are the mean with SD ≤ 12%. As a positive control daunorubicin was used, and an IC_50_ value of 2.1 ± 0.2 μM was obtained.

## Figures and Tables

**Table 1:** IC_50_ values for synthesized PROTACs.

ID	Structure	HDAC1 IC_50_ (µM) or % inhib. at Given conc.	HDAC6 IC_50_ (µM) or % inhib. at Given conc.	HDAC8 IC_50_ (µM)
CRBN-based PROTACs				
CRBN_**1a**	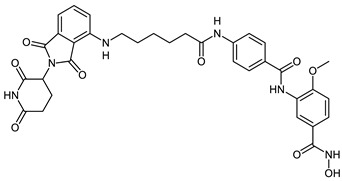	4.37 ± 0.65	0.22 ± 0.06	0.09 ± 0.03
CRBN_**1b**	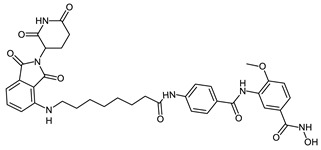	>20	0.56 ± 0.1	0.81 ± 0.17
CRBN_**1c**	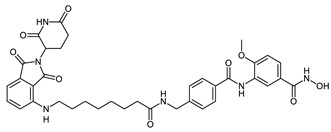	10 µM: 40.1% 1 µM: 22.2%	0.25 ± 0.02	0.15 ± 0.01
CRBN_**1d**	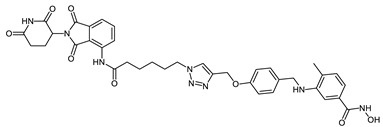	13.1 ± 0.6	6.7 ± 0.6	0.37 ± 0.05
CRBN_**1e**	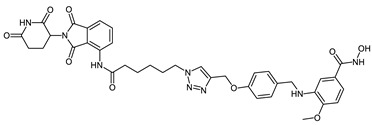	16.2 ± 0.8	17.2 ± 2.4	0.25 ± 0.07
CRBN_**1f**	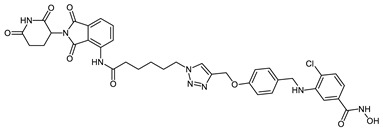	10.8 ± 0.7	1.3 ± 0.3	0.25 ± 0.03
CRBN_**1g**	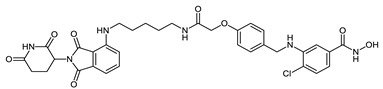	10 µM: 69% 1µM: 10.1%	10 µM: 72.6% 1µM: 24.8%	0.59 ± 0.11
CRBN_**1h**	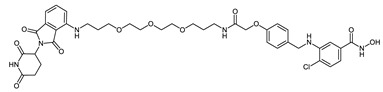	3.91 ± 0.48	10 µM: 66.8% 1µM: 33.5%	0.33 ± 0.19
CRBN_**1i**	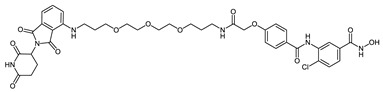	10 µM: 67.4% 1µM: 20.7%	10 µM: 92.3% 1µM: 85.4%	0.65 ± 0.14
CRBN_**1j**	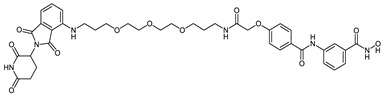	10 µM: 60.0% 1µM: 13.9%	10 µM: 80.9% 1µM: 46.2%	4.84 ± 1.05
VHL-based PROTACs				
VHL_**1k**	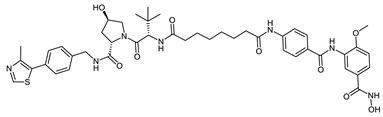	>20	0.31 ± 0.01	0.11 ± 0.01
VHL_**1l**	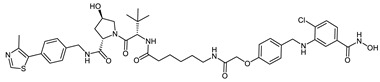	10 µM: 45.5% 1µM: 6.0%	10 µM: 72.4% 1µM: 8.3%	0.72 ± 0.15
HyT-based PROTACs				
HyT_**1m**	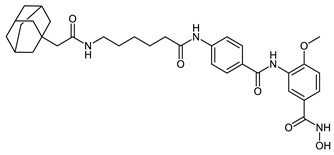	10 µM: 81.9% 1µM: 47.7%	0.25 ± 0.08	0.57 ± 0.11
HyT_**1n**	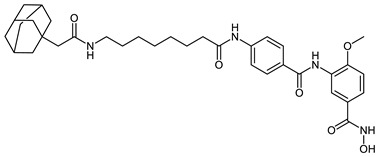	10 µM: 30.0% 1 µM: 38.6%	0.82 ± 0.05	0.39 ± 0.03
HyT_**1o**	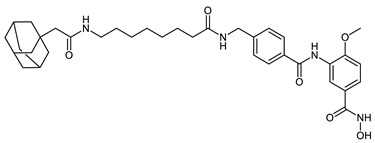	10 µM: 52.6% 1 µM: 9.8%	0.37 ± 0.03	0.09 ± 0.01
HyT_**1p**	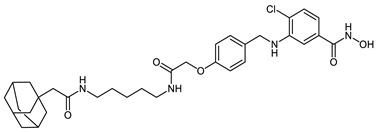	10 µM: 40.0% 1µM: 2.3%	10 µM: 72.8% 1µM: 12.4%	0.75 ± 0.09

**Table 2:** IC_50_ values for benzhydroxamate-based HADC inhibitors underlying PROTAC development.

ID	Inhibitor Structure	HDAC1 IC_50_ (µM)	HDAC6 IC_50_ (µM)	HDAC8 IC_50_ (µM)
**2a**	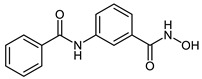	33.6 ± 1.8	3.0 ± 0.3	0.58 ± 0.05
**2b**	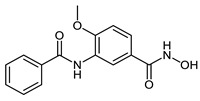	2.3 ± 1.2	2.5 ± 1.1	0.09 ± 0.02
**2c**	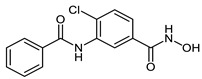	11.6 ± 3.9	0.12 ± 0.02	0.12 ± 0.04
**2d**	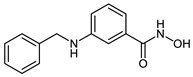	2.3 ± 1.2	2.5 ± 1.1	0.14 ± 0.01
**2e**	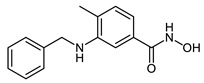	21.8 ± 2.1	5.1 ± 0.3	0.26 ± 0.04
**2f**	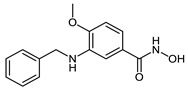	14.5 ± 1.4	5.1 ± 0.8	0.07 ± 0.02
**2g**	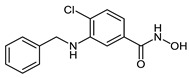	10.4 ± 1.2	4.0 ± 0.2	0.25 ± 0.04
**2h**	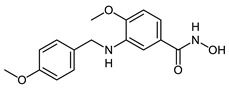	>20	0.15 ± 0.001	0.01 ± 0.001
**2i**	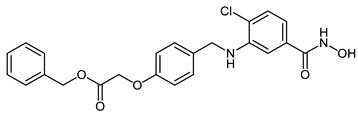	>20	7.4 ± 0.6	0.41 ± 0.05

**Table 3:** Cytotoxicity on HEK293 cells (cell viability at 50 µM inhibitor treatment). Daunorubicin was used as positive control.

ID	HEK293 viability 50 µM	ID	HEK293 viability 50 µM	ID	HEK293 viability 50 µM
2a	72.0 ± 2.9	CRBN_1a	85.6 ± 2.4	CRBN_1j	83.7 ± 3.5
2b	67.3 ± 3.9	CRBN_1b	64.1 ± 1.7	VHL_1k	78.2 ± 3.7
2c	72.2 ± 3.5	CRBN_1c	70.1 ± 6.7	VHL_1l	90.4 ± 2.3
2d	78.8 ± 6.1	CRBN_1d	80.2 ± 2.8	HyT_1m	51.5 ± 6.2
2e	90.4 ± 1.7	CRBN_1e	65.1 ± 4.3	HyT_1n	60.0 ± 3.7
2f	68.1 ± 1.2	CRBN_1f	100.5 ± 2.8	HyT_1o	69.3 ± 1.2
2g	87.4 + 3.4	CRBN_1g	68.2 ± 2.5	HyT_1p	80.1 ± 1.9
2h	70.4 ± 7.5	CRBN_1h	61.3 ± 0.9	Daunorubicin	IC_50_ 2.1 ± 0.2 μM
2i	88.1 ± 0.1	CRBN_1i	97.6 ± 7.0		

## Data Availability

Not applicable.

## Supplementary Materials

The following supporting information can be downloaded at: https://www.mdpi.com/article/10.3390/ijms23147535/s1. References [16,50,62,63,64,65,66,67,68,69,70,71,72,73] are cited in the Supplementary Materials.
