# Supplementary material for: Design, Synthesis and Biological Characterization of Histone Deacetylase 8 (HDAC8) Proteolysis Targeting Chimeras (PROTACs) with Anti-Neuroblastoma Activity
Source: Int J Mol Sci. 2022 Jul 7;23(14):7535. doi: 10.3390/ijms23147535 (PMC9322761; doi:10.3390/ijms23147535)
Supplement: Supplementary file 1 [file ijms-23-07535-s001.zip › ijms-1763408-supplementary.pdf]

Design, Synthesis and Biological Characterization of Histone Deacetylase 8 (HDAC8) Proteolysis Targeting Chimeras (PROTACs) with Anti-Neuroblastoma Activity

Salma Darwish[1, 2], Ehab Ghazy[1, 2], Tino Heimburg[1], Daniel Herp[3], Patrik Zeyen[1], Rabia Salem-Altintas[4, 5, 6,7], Johannes Ridinger[4, 5, 6], Dina Robaa[1], Karin Schmidtkunz[3], Frank Erdmann[1], Matthias Schmidt[1], Christophe Romier[8], Manfred Jung[3], Ina Oehme[4, 5, 6] and Wolfgang Sippl[1, *]

[1] Department of Medicinal Chemistry, Institute of Pharmacy, Martin-Luther University of Halle-Wittenberg, 06120 Halle (Saale), Germany

[2] Department of Pharmaceutical Chemistry, Faculty of Pharmacy, Alexandria University, 21521 Alexandria, Egypt

[3] Institute of Pharmaceutical Sciences, University of Freiburg, 79104 Freiburg, Germany

[4] Hopp Children's Cancer Center Heidelberg (KiTZ), 69120 Heidelberg, Germany

[5] Clinical Cooperation Unit Pediatric Oncology, German Cancer Research Center (DKFZ), 69120 Heidelberg, Germany

[6] German Cancer Consortium (DKTK), 69120 Heidelberg, Germany

[7] Faculty of Medicine, Heidelberg University, 69120 Heidelberg, Germany

[8] Département de Biologie Structurale Intégrative, Institut de Génétique et de Biologie Moléculaire et Cellulaire (IGBMC), Centre national de la recherche scientifique, Institut national de la santé et de la recherche médicale, Université de Strasbourg, CEDEX, 67404 Illkirch, France

* Correspondence: wolfgang.sippl@pharmazie.uni-halle.de ; Tel.: +493455525040.

Table of content

	Topic	Page
**S1**	**Synthesis of HDAC8 inhibitors (2h and 2i)**	**2**
**S2**	**Synthesis of negative controls (33a and 33b)**	**3**
**S3**	**Synthesis of E3 ligase ligands (Pomalidomide, 4-Fluorothalidomide, VHL ligand)**	**5**
**S4**	**Synthesis of E3 ligase ligand-linker-conjugates and HyT-linker-conjugates (Synthesis of E3 ligase ligand-linker-COOH and HyT-linker-COOH conjugates, E3 ligase ligand-linker-N_3, and Synthesis of E3 ligase ligand-linker-NH_2 and HyT-linker-NH_2)**	**7**
**S5**	**Characterization data of final compounds, intermediates and relevant compounds (HPLC, ^{1}HNMR, ^{13}CNMR, HRMS)**	**10**
**S6**	**Non- Enzymatic stability data for CRBN_1b and CRBN_1e**	**65**

**S7**	**HDAC8 degradation by CRBN1_e in combination with the de novo synthesis inhibitor cycloheximide**	**66**
**S8**	**HDAC6 degradation and tubulin hyperacetylation by PROTACs and control compounds**	**67**

S1 Synthesis of HDAC8 inhibitors (2h and 2i)

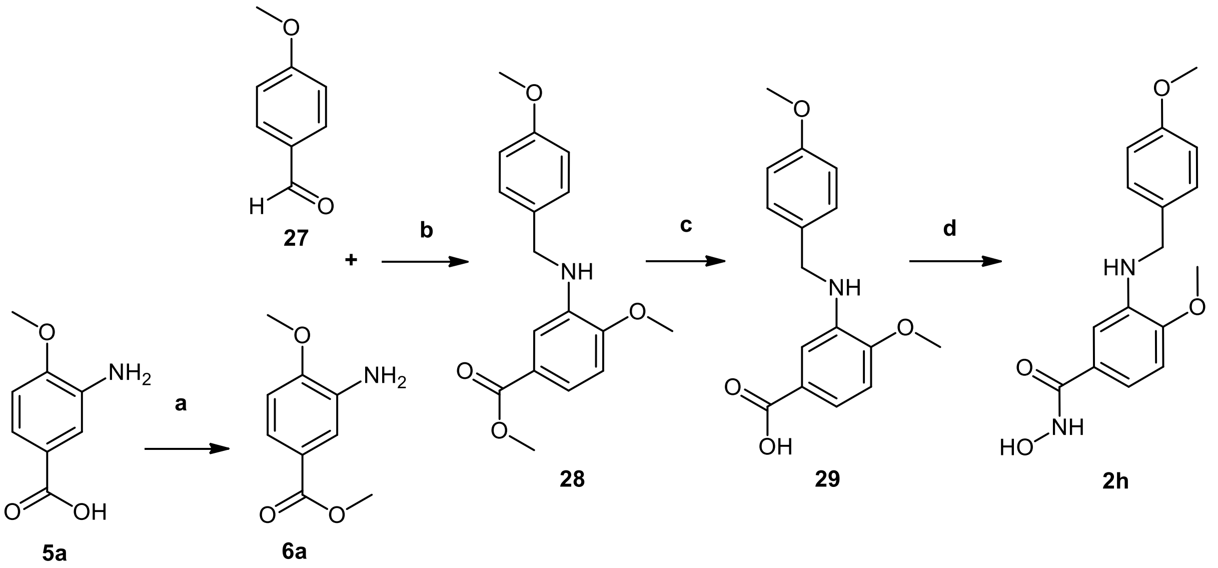

Scheme S1.1: Reagents and conditions: (a) MeOH, $SOCl_2$, reflux, 3hrs; (b) 1) toluene, reflux, 2h; 2) THF, 0°C, CH_3COOH, $Na(CH_3COO)_3BH$, 30min; 3) room temperature, 24h; (c) 1M NaOH, MeOH, reflux, 4h; (d) 1) DIPEA, HATU, NH_2OTHP, DMF, 50°C, 24h; 2) 1M HCl, THF, US, room temperature, 2h.

Methyl 4-methoxy-3-((4-methoxybenzyl)amino)benzoate (28) was prepared from 4-methoxybenzaldehyde (27) and methyl 3-amino-4-methoxybenzoate (6a) according to the general method IA. It was then hydrolysed according to the method IIA to yield 4-methoxy-3-((4-methoxybenzyl)amino)benzoic acid (29), which was reacted with *O*-(tetrahydro-2*H*-pyran-2-yl)hydroxylamine following method IIIA. Finally, the 2-tetrahydropyranyl-protected compound was hydrolysed following method V to yield the final HDAC8 inhibitor *N*-hydroxy-4-methoxy-3-((4-methoxybenzyl)amino)benzamide (2h).

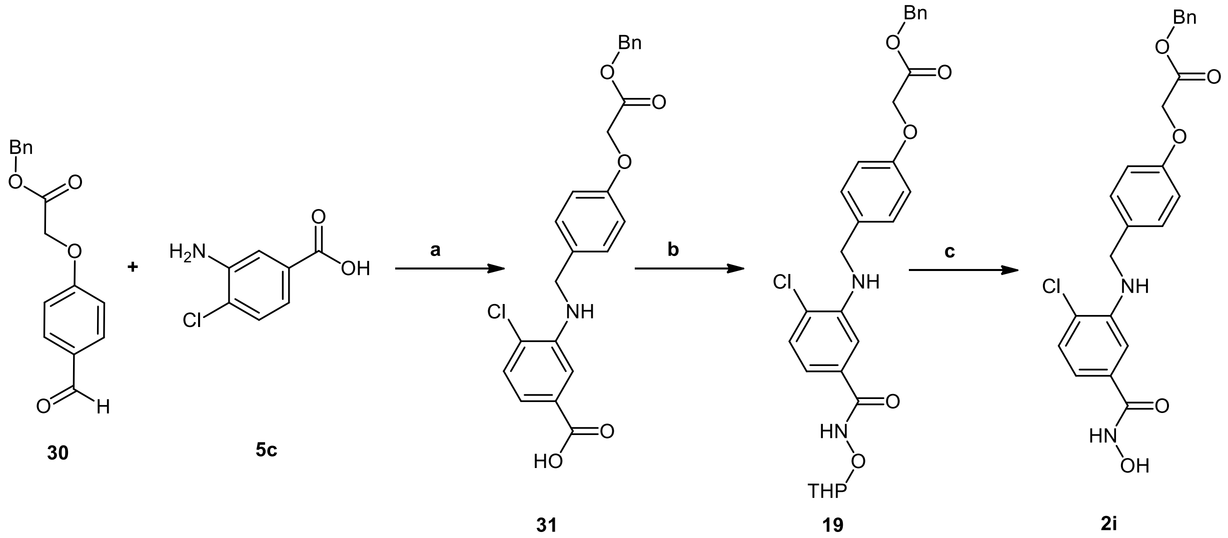

Scheme S1.2: Reagents and conditions: (a) $Na(CH_3COO)_3BH$, TFA, EtoAc: THF (1:1), room temperature, 2h; (b) DIPEA, HATU, NH_2OTHP, DMF, rt., 5h; (c) 1M HCl, THF, room temperature.

Benzyl 2-(4-formylphenoxy)acetate (30) was prepared as previously described [62] then it was reacted with 3-amino-4-chlorobenzoic acid (5c) using method IB to afford 3-({4-[2-(benzyloxy)-2-oxoethoxy]benzyl}amino)-4-chlorobenzoic acid (31). The latter was coupled with O-(tetrahydro-2H-pyran-2-yl) hydroxylamine using method IIIA to yield the 2-tetrahydropyranyl-protected compound (19) which was finally deprotected using method V to afford the final HDAC8 inhibitor (2i).

The synthesis schemes of inhibitors 2a-g are reported in previous publications [16,50].

S2 Synthesis of negative controls (33a and 33b)

- **Negative control 33a**

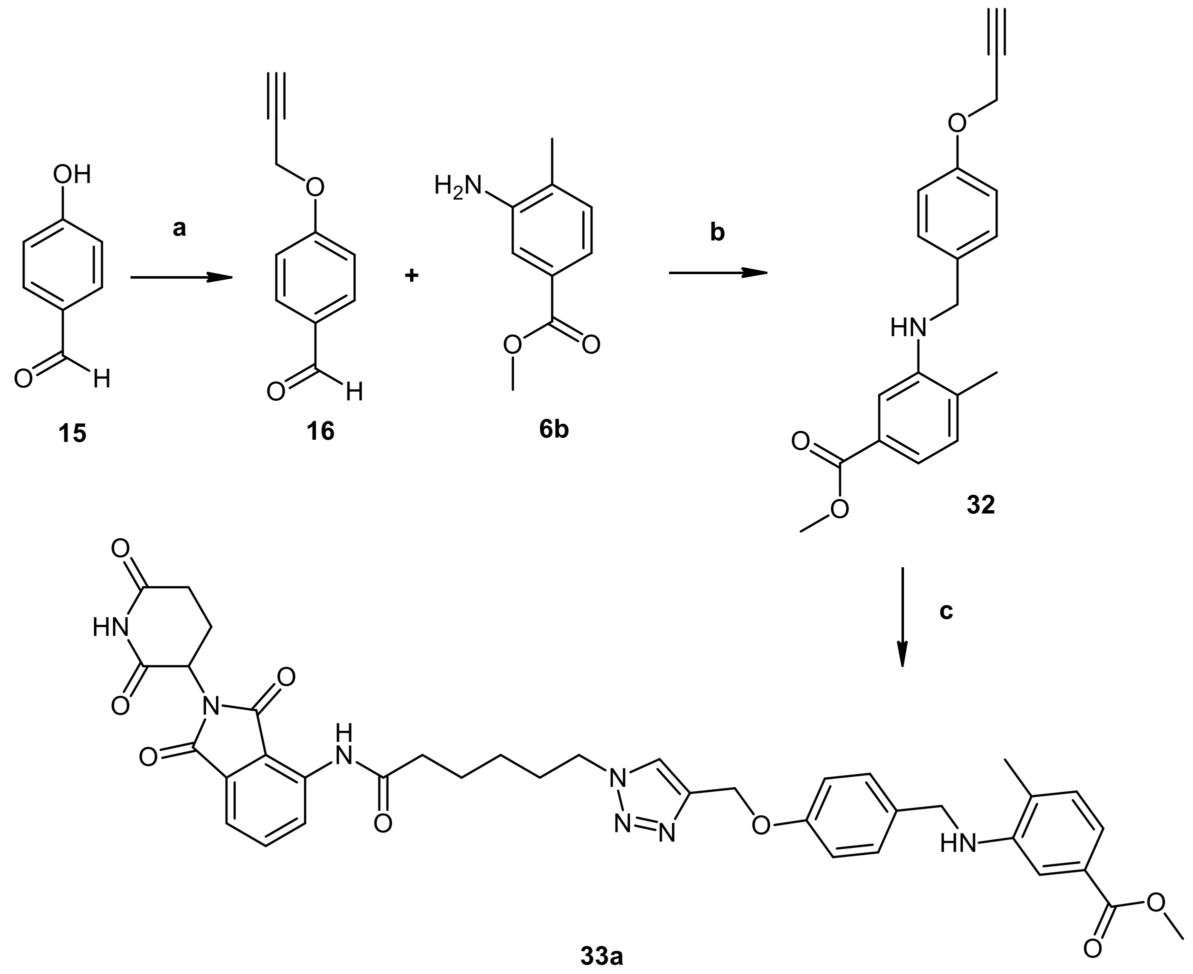

Scheme S2.1: (a) 1) K_2CO_3, DMF, room temperature, 1hr; 2) propargyl bromide at 0°C; 3) 24hrs at room temperature; (b) 1) toluene, reflux, 2hr; 2) THF, 0°C, CH_3COOH, $Na(CH_3COO)_3BH$, 30min; 3) room temperature, 24h; (c) 1) E3 ligase ligand–linker-N_3 50, THF / H_2O, Sod. Ascorbate, $CuSO_4$ x $5H_2O$, 24h, room temperature.

To prepare the methyl 4-methyl-3-((4-(proparg-1-yloxy)benzyl)amino)benzoate (32), 4-proparg-1-yloxybenzaldehyde (16) and methyl 3-amino-4-methylbenzoate (6b) were reacted according to the method IA. Afterwards, the formed ligand was linked to the E3 ligase ligand–linker-N_3 (50) via the azide-alkyne Huisgen cycloaddition reaction as stated in method IV to give compound (33). The compound was confirmed by ^{1}HNMR and mass (see S5 for details).

- **Negative control 33b**

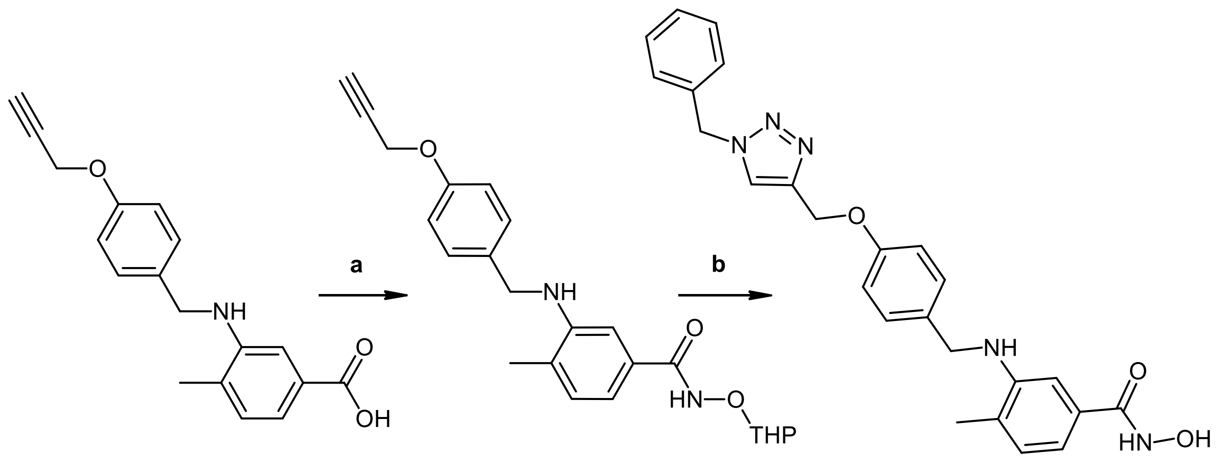

17b **18 b** **33b**

Scheme S2.2: (a) DIPEA, HATU, NH_2OTHP, DMF, 50°C, 24h; (b) 1) $C_7H_7N_3$, THF / H_2O, Sod. Ascorbate, $CuSO_4$ x $5H_2O$, 24h, room temperature; 2) 1M HCl, THF, US, room temperature, 2h.

After the 2-tetrahydropyranyl-protected compound (18b) was prepared by reacting the free carboxylic acid (17b) with O-(tetrahydro-2H-pyran-2-yl)hydroxylamine following the general method IIIA, it was linked to benzyl azide via the azide-alkyne Huisgen cycloaddition as stated in method IV. Finally, the free hydroxamic acid was obtained by removing the 2-tetrahydropyranyl group following method V. The compound was confirmed by ^{1}HNMR (see S5 for details).

S3 Synthesis of E3 ligase ligands

- **Pomalidomide (38)**

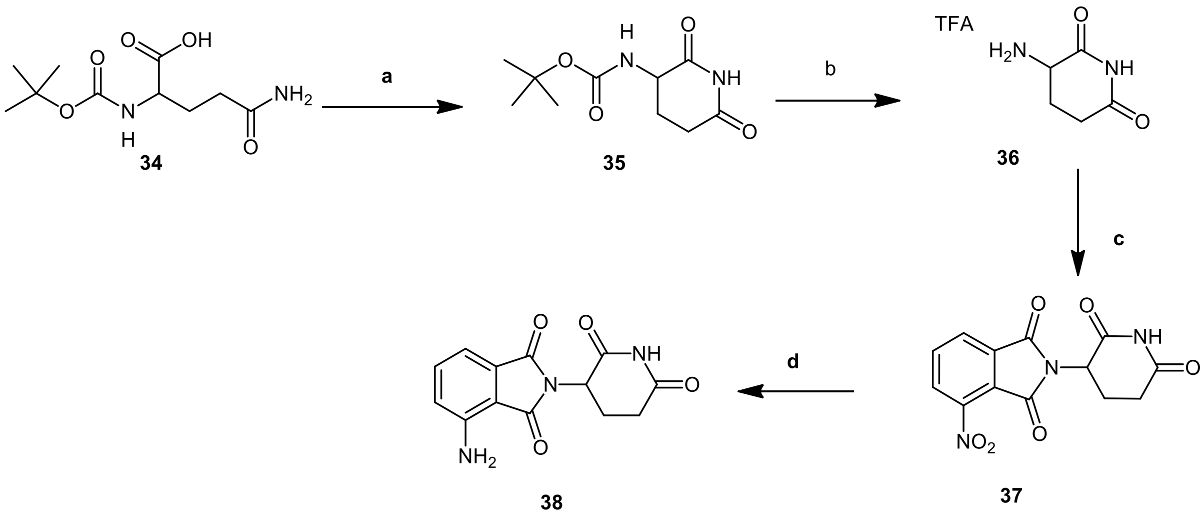

Scheme S3.1: a) CDI, THF, Mw 20 min; b) TFA, DCM, rt, 30 min; c) 1) 3- Nitrophthalic acid anhydride, DIPEA, THF, rt, 15 min; 2) $SOCl_2$, rt, Overnight; d) $SnCl_2 \cdot 2H_2O$, EtOH, relux, 1 hr.

In the microwave a solution of Boc-L-glutamin (34, 1 eq.) and 1,1′-Carbonyldiimidazol (1,25 eq.) in tetrahydrofuran was heated at 150°C for 20 min. After the reaction ends the reaction mixture was cooled in an ice bath for 30 min. Afterwards the precipitated tert-butyl (2,6-dioxopiperidin-3-yl)carbamate (35) was filtered using a sintered glass funnel and was washed with cold tetrahydrofuran. (yield: 80%)

In the next step tert-butyl (2,6-dioxopiperidin-3-yl)carbamate (35) was stirred in a mixture of dichloromethane and trifluoroacetic acid (4:1) at room temperature for 30min. After completion of the reaction the solvent and excess trifluoroacetic acid were evaporated using the rotary evaporator to yield 3-aminopiperidine-2,6-dione (36) as a trifluoroacetic acid salt which was used without further purification.

The crude product was dissolved with DIPEA (1:4) in tetrahydrofuran. Afterwards 3-Nitrophthalic acid anhydride (1 eq.) was added and the reaction mixture was stirred at room temperature for 15 min. Thionyl chloride (3 eq.) was then added dropwise and stirring was continued for 24 hr at room temperature. After completion of the reaction the solvent is evaporated under reduced pressure. The remaining residue, 2-(2,6-dioxopiperidin-3-yl)-4-nitroisoindoline-1,3-dione (37) was washed with cold water and filtered using a sintered glass funnel.

In the last step, 2-(2,6-dioxopiperidin-3-yl)-4-nitroisoindoline-1,3-dione (37) was dissolved in ethanol and stannous chloride dihydrate (5 eq.) was added to it. The formed mixture was then refluxed for 1 hr. After the completion of the reaction the solvent was

removed under reduced pressure and the remaining residue was washed with water and filtered using a porcelain funnel and was left to dry in a desiccator. The compound was confirmed by ^{1}HNMR and ^{13}CNMR (see S5 for details).

- **4-Fluorothalidomide (39)**

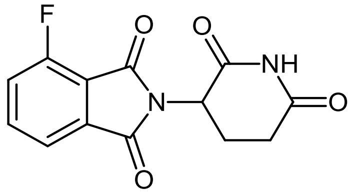

This compound was synthesized according to a previously reported procedure [63]. The obtained product was used without further purification. The compound was confirmed with ^{1}HNMR and mass (see S5 for details).

- **VHL ligand (40)**

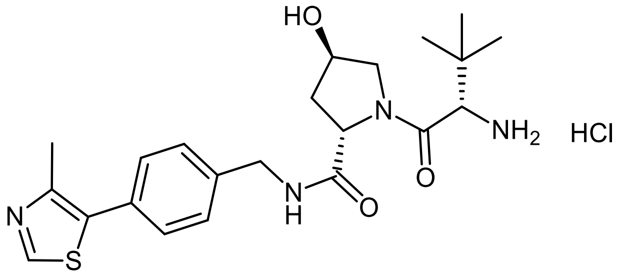

A previously reported procedure [64] was followed to synthesize this compound. The compound was confirmed with ^{1}HNMR and mass (see S5 for details).

S4 Synthesis of E3 ligase ligand-linker-conjugates and HyT-linker-conjugates

A. Synthesis of E3 ligase ligand-linker-COOH and HyT-linker-COOH conjugates

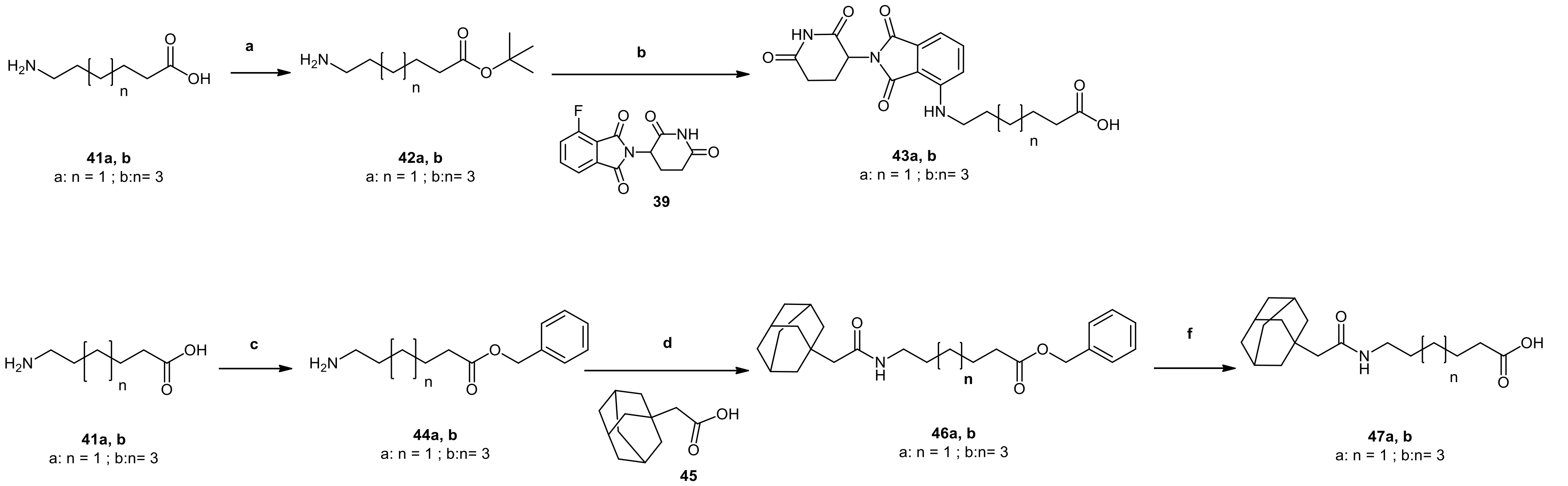

Scheme S4.1: (a) 1) $SOCl_2$, room temperature, 2h; 2) $NaHCO_3$ in tert-butanol, room temperature, 2h; (b) 1) DIPEA, NMP, 110°C, 24h; 2) DCM, TFA, room temperature, 24h; (c) TsOH H_2O, BnOH, reflux, 6h, room temperature, 24h; (d) DIPEA, HATU, DMF, room temperature, 24h; (e) Pd / C (5%), H_2, THF, 24h.

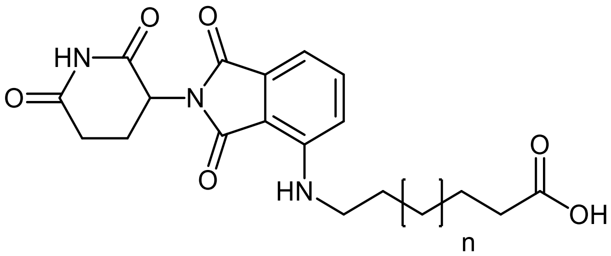

43a, b
a: n = 1 ; b:n= 3

The aminoalkanoic acids (41a,b) were protected according to a reported procedure [65] to yield the tert-butyl esters (42a,b) that were reacted with the 4-Fluorothalidomide (39) as previously published [66]. Finally, the tert-butyl ester protecting groups were removed following method VI. The compounds were confirmed with ^{1}HNMR and mass.

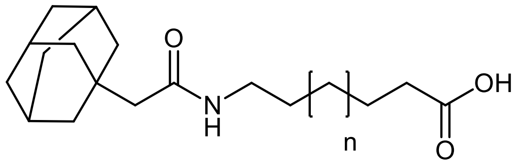

47a, b
a: n = 1 ; b:n= 3

The aminoalkanoic benzyl acid esters (44a,b) were prepared as reported [67]. They were then reacted with adamantane acetic acid (45) according to the general method IIIA. Finally, the benzyl protecting group was removed using the general method VII to yield the free carboxylic acid group. The compounds were confirmed with ^{1}HNMR and/or mass.

B. Synthesis of E3 ligase ligand-linker-N_3

6-Azido-N-(2-(2,6-dioxopiperidin-3-yl)-1,3-dioxoisoindolin-4-yl)hexanamide (50)

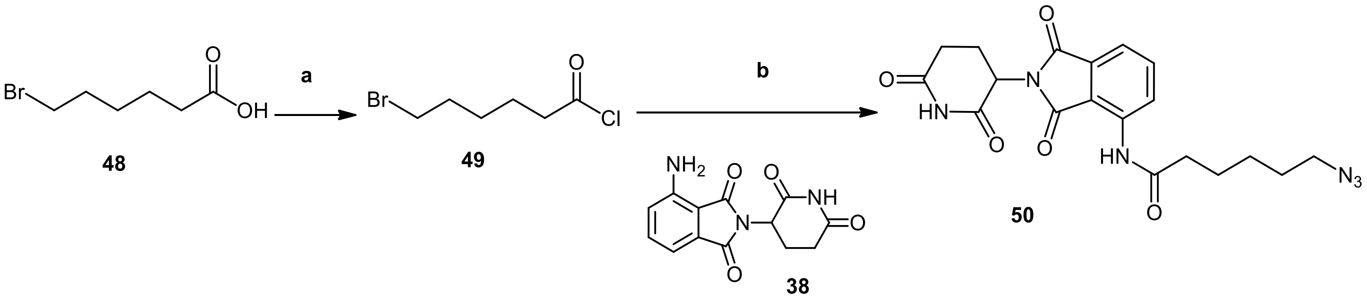

Scheme S4.2: (a) $C_2O_2Cl_2$, Et_2O, DMF, 0°C, room temperature, 5h; (b) 1) DIPEA, THF, reflux, 4h; 2) NaN_3, DMF, 70–80°C, 24h.

6-bromohexanoyl chloride (49) was synthesized following a reported procedure [68]. The acid chloride was reacted with pomalidomide (38) as reported to yield 6-bromo-N-(2-(2,6-dioxopiperidin-3-yl)-1,3-dioxoisoindolin-4-yl)hexanamide. The latter was converted into the azide derivative [69]. The formation of the compound was confirmed by mass.

C. Synthesis of E3 ligase ligand-linker-NH_2 and HyT-linker-NH_2

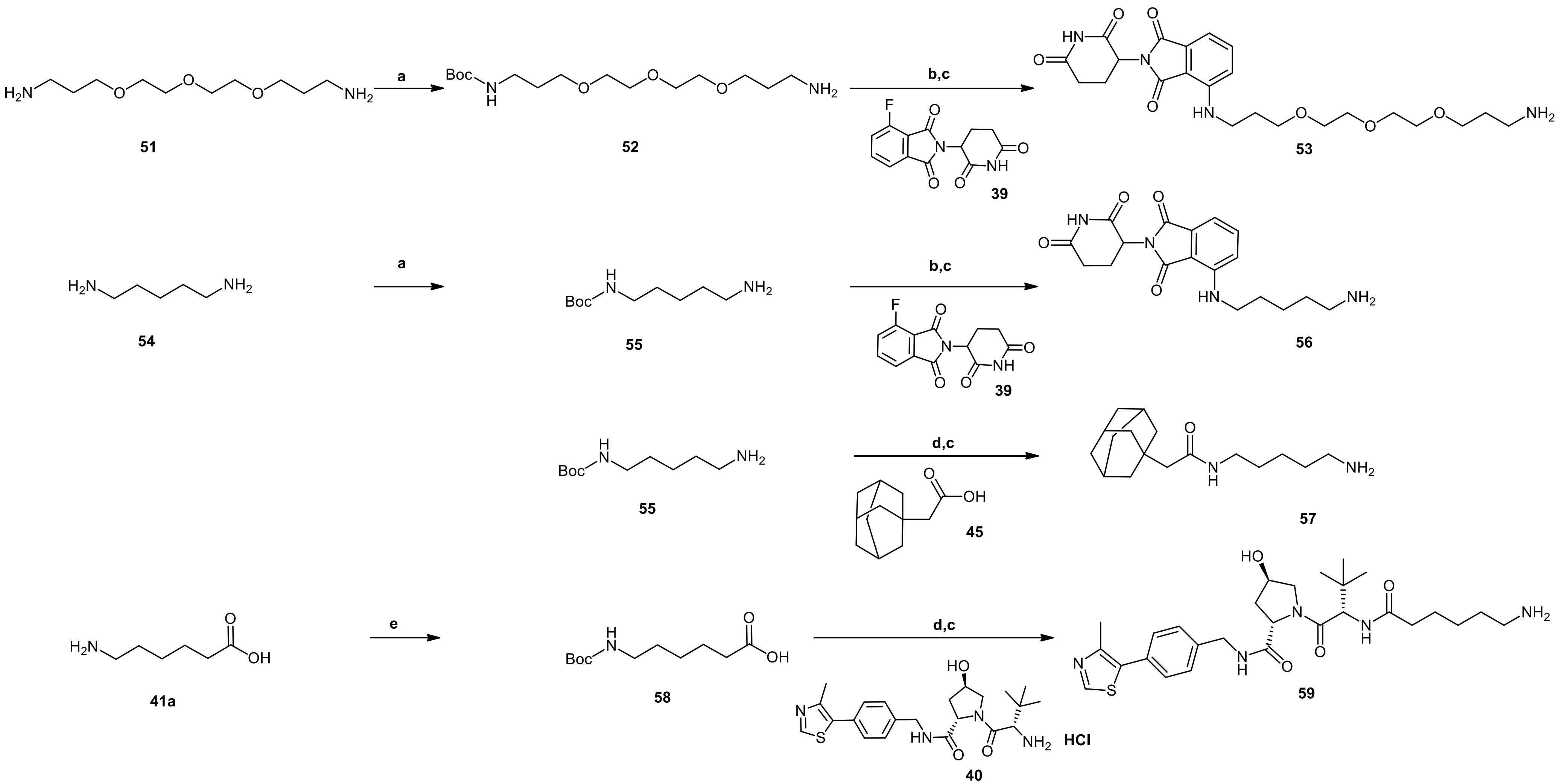

Scheme S4.3: Reagents and conditions: (a) Boc-anhydride, 1,4-dioxane [70]; (b) DIPEA, DMF, 90°C, 8 hours [71]; (c) trifluoroacetic acid, DCM; (d) HATU, DIPEA, DMF; (e) Boc-anhydride, 1 M NaOH, 1,4-dioxane: H_2O (1:1) [72]

S5 Characterization data of final compounds, intermediates and relevant compounds

Abbreviation	Meaning
br	broad
s	singlet
d	doublet
t	triplet
dd	doublet of doublets
tt	triplet of triplet
dt	doublet of triplets
m	multiplet
ddd	doublet of doublets of doublets

3-(4-(6-((2-(2,6-dioxopiperidin-3-yl)-1,3-dioxoisoindolin-4-yl)amino)hexanamido)benzamido)-N-hydroxy-4-methoxybenzamide (CRBN_1a)

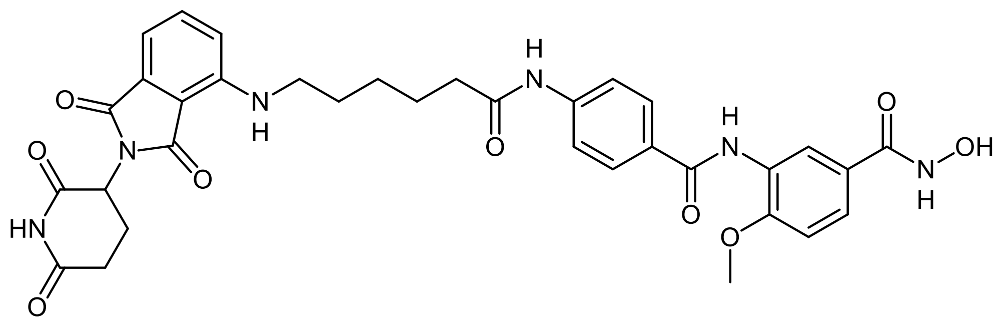

MS m/z: 669.34 $[M-H]^-$

^{1}H NMR (400 MHz, DMSO-d6) δ 11.06 (br s, 2H), 10.14 (s, 1H), 9.36 (s, 1H), 8.91 (br s, 1H) 8.18 (d, J = 2.1 Hz, 1H), 7.90 (d, J = 8.8 Hz, 2H), 7.71 (d, J = 8.7 Hz, 2H), 7.62 – 7.52 (m, 2H), 7.12 (d, J = 8.7 Hz, 1H), 7.09 (d, J = 8.6 Hz, 1H), 7.00 (d, J = 7.0 Hz, 1H), 6.53 (t, J = 5.9 Hz, 1H), 5.03 (dd, J = 12.8, 5.4 Hz, 1H), 3.86 (s, 3H), 2.94 – 2.78 (m, 2H), 2.68 – 2.51 (m, 2H), 2.35 (t, J = 7.3 Hz, 2H), 2.09 – 1.90 (m, 2H), 1.63 (tt, J = 15.0, 7.3 Hz, 4H), 1.45 – 1.32 (m, 2H).

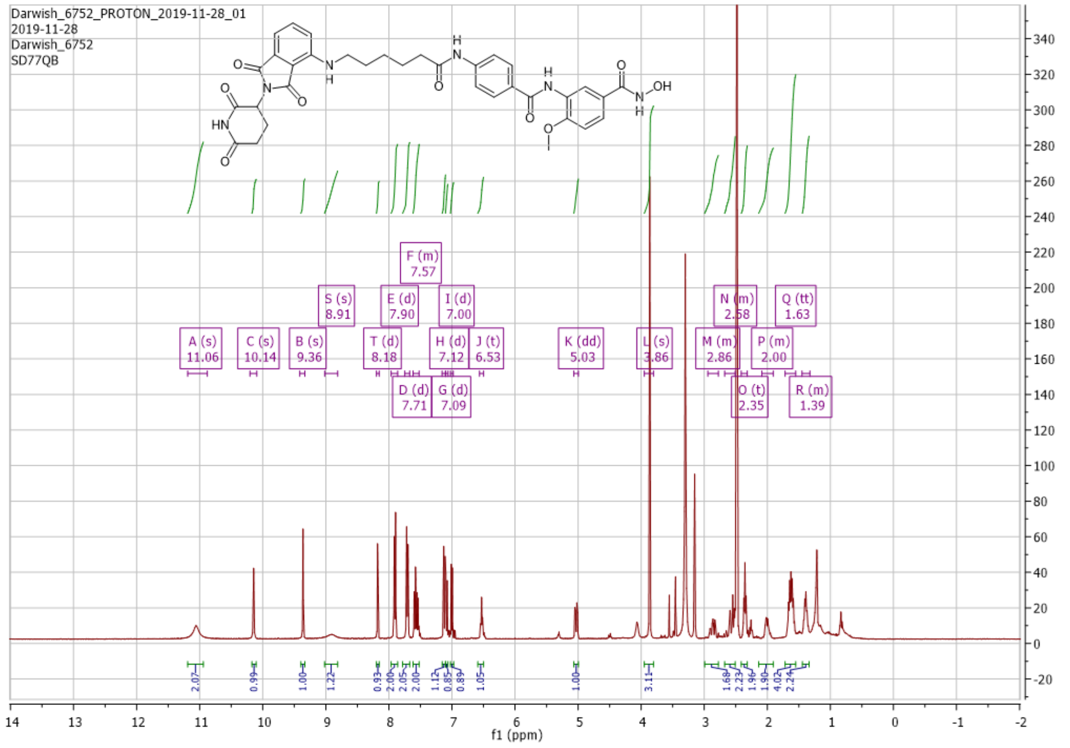

^{13}C NMR (101 MHz, DMSO-d6) δ 173.34, 172.28, 170.55, 169.38, 167.78, 165.05, 154.29, 146.87, 142.77, 136.79, 132.55, 128.86, 128.76, 127.04, 118.82, 118.53, 111.33, 110.89, 109.37, 56.47, 51.59, 49.03, 48.97, 36.81, 31.37, 28.91, 26.37, 25.17, 22.58.

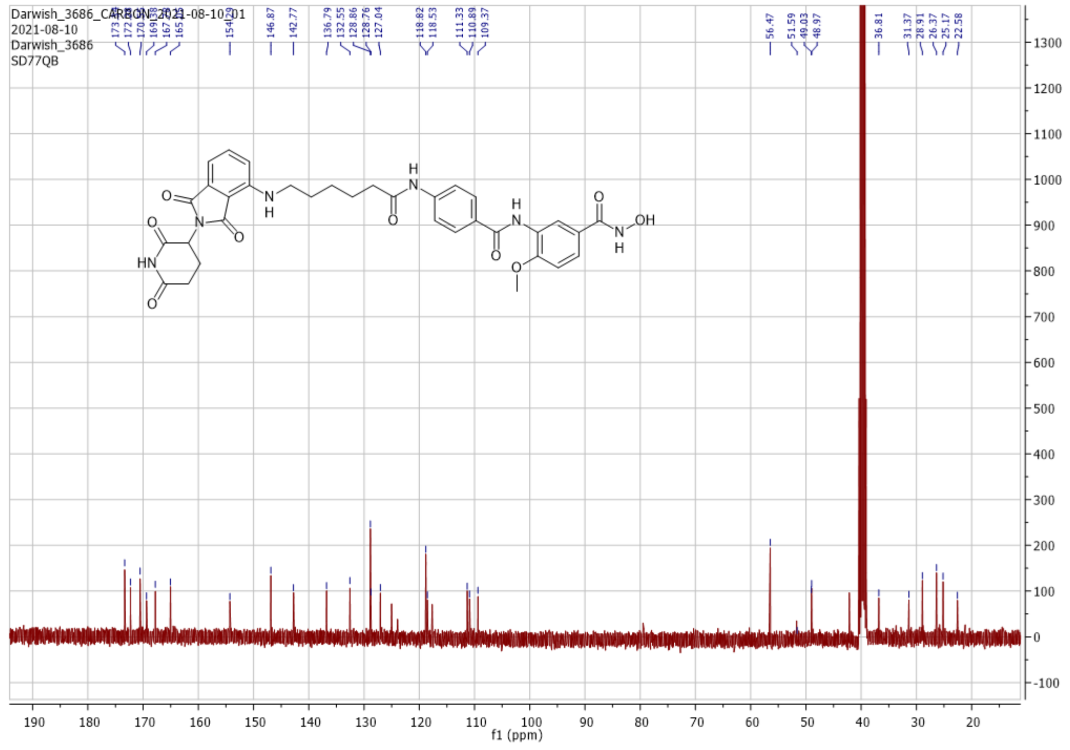

HRMS: 693.227 [M+Na]$^+$, calculated $C_{34}H_{34}N_6O_9Na^+$: 693.228

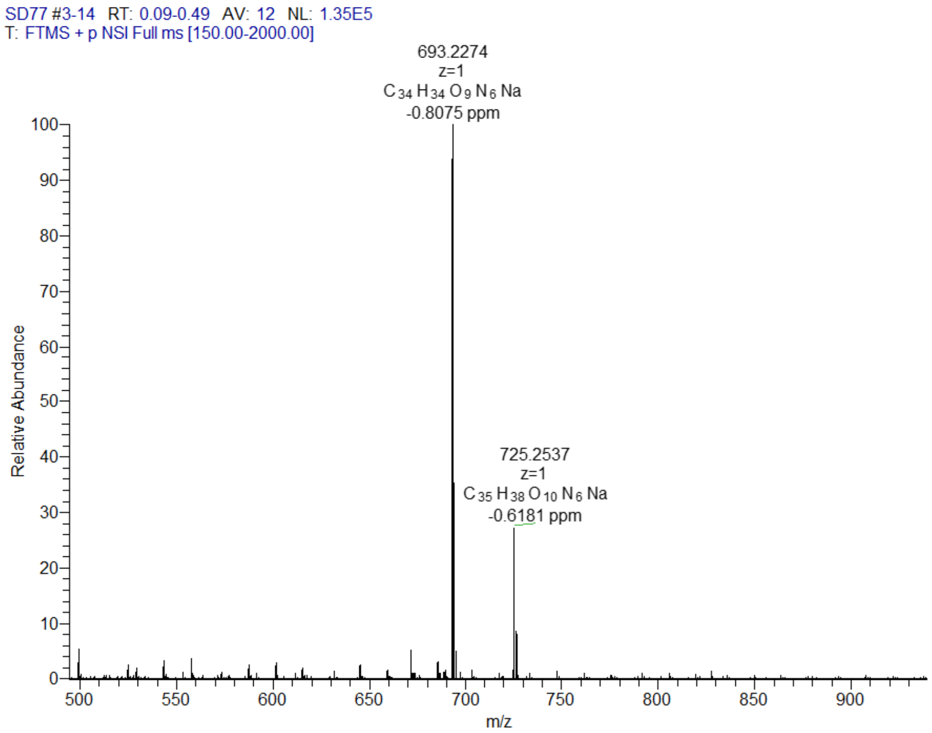

HPLC: (M2) rt. 9.7 min (purity >99%)

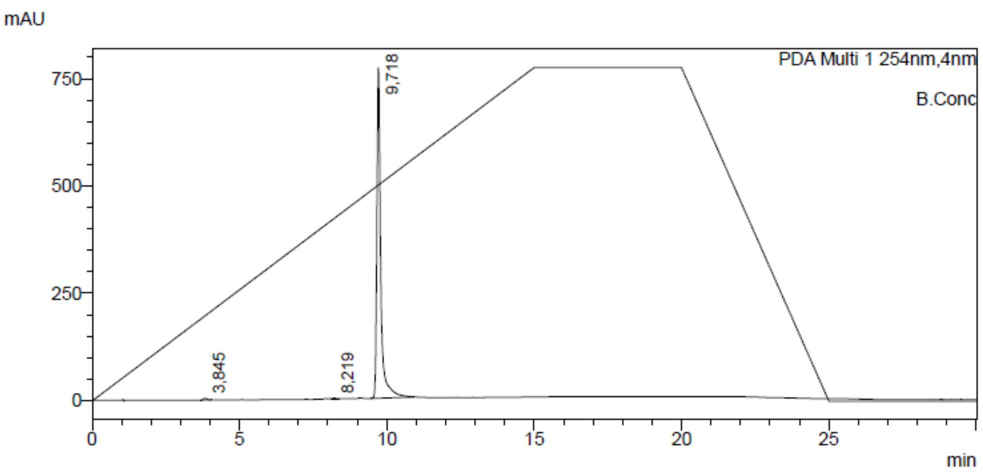

PDA Ch1 254nm

Peak#	Ret. Time	Area	Height	Area%
1	3,845	38161	4122	0,539
2	8,219	13298	1726	0,188
3	9,718	7023538	770661	99,273
Total		7074997	776509	100,000

3-(4-(8-((2-(2,6-Dioxopiperidin-3-yl)-1,3-dioxoisoindolin-4-yl)amino)octanamido)benzamido)-N-hydroxy-4-methoxybenzamide (CRBN_1b)

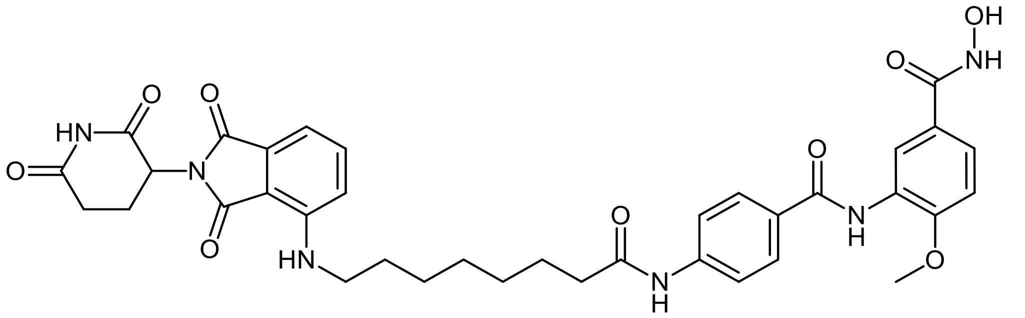

MS m/z: 697.62 [M-H]$^-$

^{1}H NMR (400 MHz, DMSO-d6) δ 11.05 (br s, 2H), 10.14 (s, 1H), 9.36 (s, 1H), 8.91 (br s, 1H), 8.17 (d, J = 1.7 Hz, 1H), 7.90 (d, J = 8.7 Hz, 2H), 7.71 (d, J = 8.5 Hz, 2H), 7.64 – 7.49 (m, 2H), 7.12 (d, J = 8.7 Hz, 1H), 7.07 (d, J = 8.6 Hz, 1H), 7.00 (d, J = 7.0 Hz, 1H), 6.51 (t, J = 5.5 Hz, 1H), 5.03 (dd, J = 12.9, 5.2 Hz, 1H), 3.86 (s, 3H), 2.95 – 2.75 (m, 2H), 2.67 – 2.52 (m, 2H), 2.39 – 2.27 (m, 2H), 2.09 – 1.92 (m, 2H), 1.68 – 1.47 (m, 4H), 1.40 – 1.23 (m, 6H).

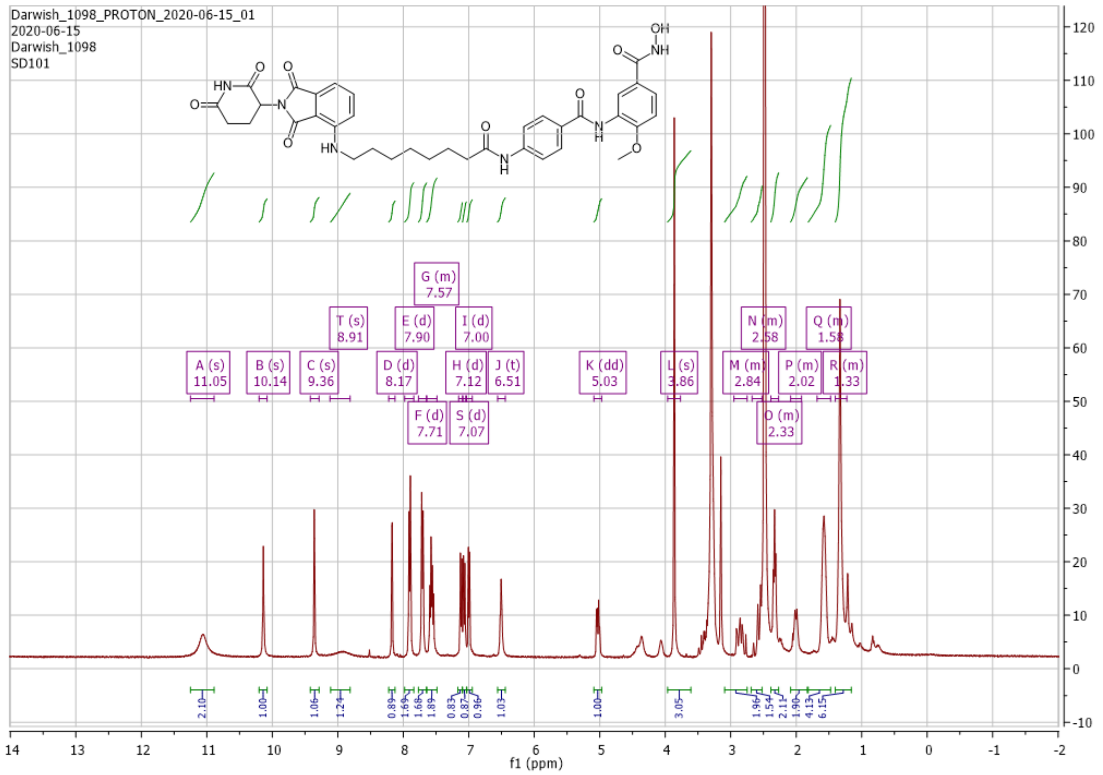

^{13}C NMR (101 MHz, DMSO-d6) δ 173.25, 172.24, 170.52, 167.75, 164.94, 146.88, 142.84, 136.74, 132.61, 128.87, 118.74, 110.83, 109.43, 72.91, 63.51, 56.48, 48.99, 36.78, 31.41, 29.09, 29.04, 28.94, 26.63, 25.37.

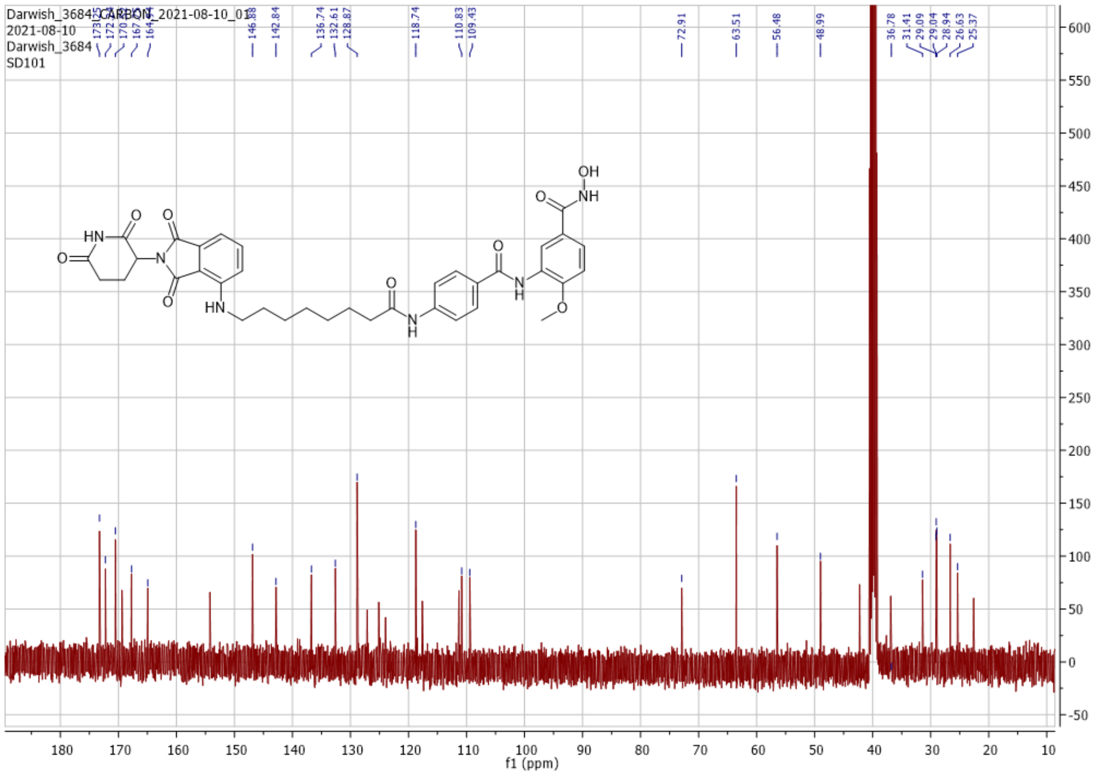

HRMS: 699.279 $[M+H]^+$, calculated $C_{36}H_{39}N_6O_9^+$: 699.278

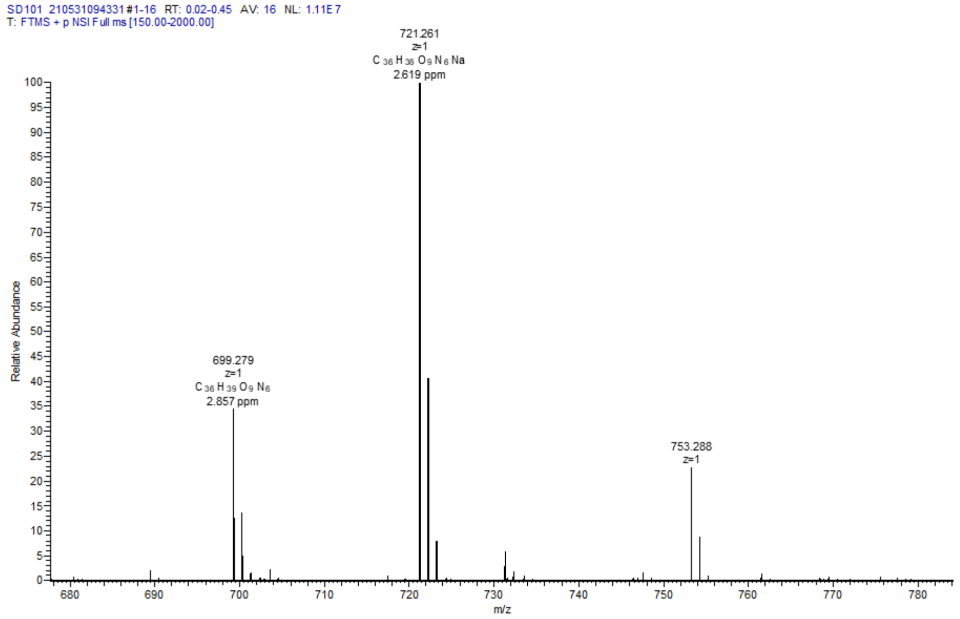

HPLC: (M2) rt. 10.3 min (purity 99%)

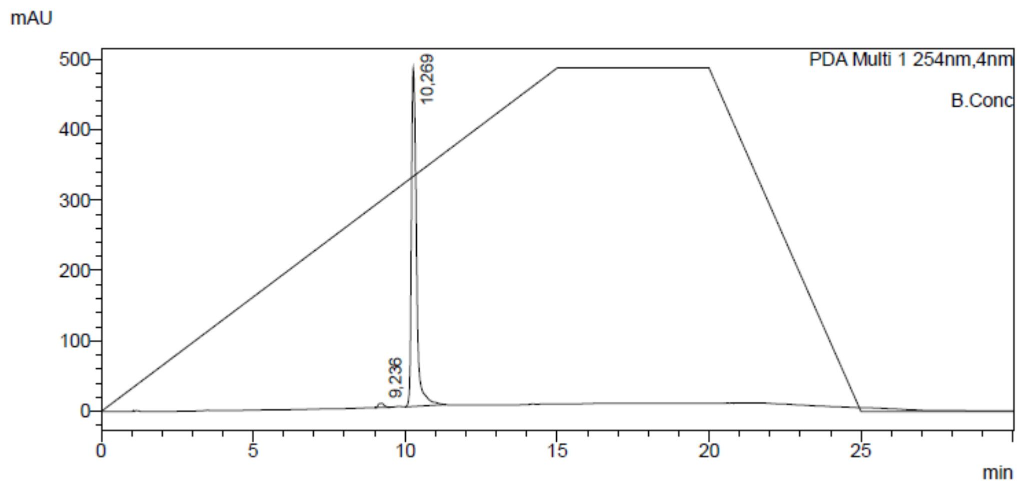

PDA Ch1 254nm

Peak#	Ret. Time	Area	Height	Area%
1	9,236	77842	5901	1,377
2	10,269	5574561	480254	98,623
Total		5652403	486156	100,000

3-(4-(6-(2-(Adamantan-1-yl)acetamido)hexanamido)benzamido)-N-hydroxy-4-methoxybenzamide (HyT_1m)

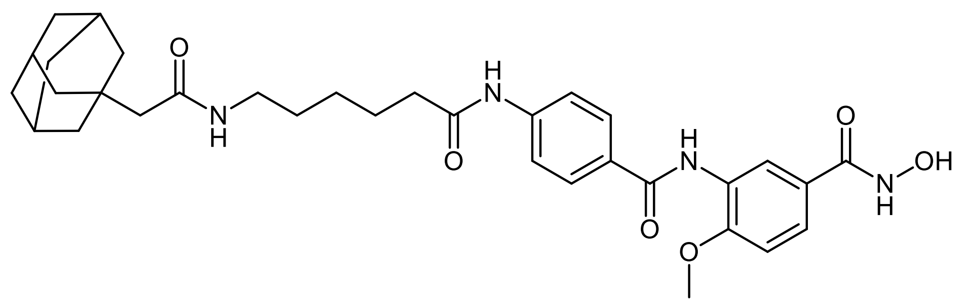

MS m/z: 589.45 [M-H]$^{-}$

^{1}H NMR (400 MHz, DMSO-d6) δ 11.05 (br s, 1H), 10.14 (s, 1H), 9.36 (s, 1H), 8.92 (br s, 1H), 8.18 (d, J = 2.1 Hz, 1H), 7.91 (d, J = 8.7 Hz, 2H), 7.71 (d, J = 8.7 Hz, 2H), 7.67 – 7.55 (m, 2H), 7.12 (d, J = 8.7 Hz, 1H), 3.87 (s, 3H), 3.00 (dd, J = 12.6, 6.6 Hz, 2H), 2.33 (t, J = 7.3 Hz, 2H), 1.94 – 1.84 (m, 3H), 1.79 (s, 2H), 1.69 – 1.47 (m, 14H), 1.46 – 1.35 (m, 2H), 1.35 – 1.24 (m, 2H).

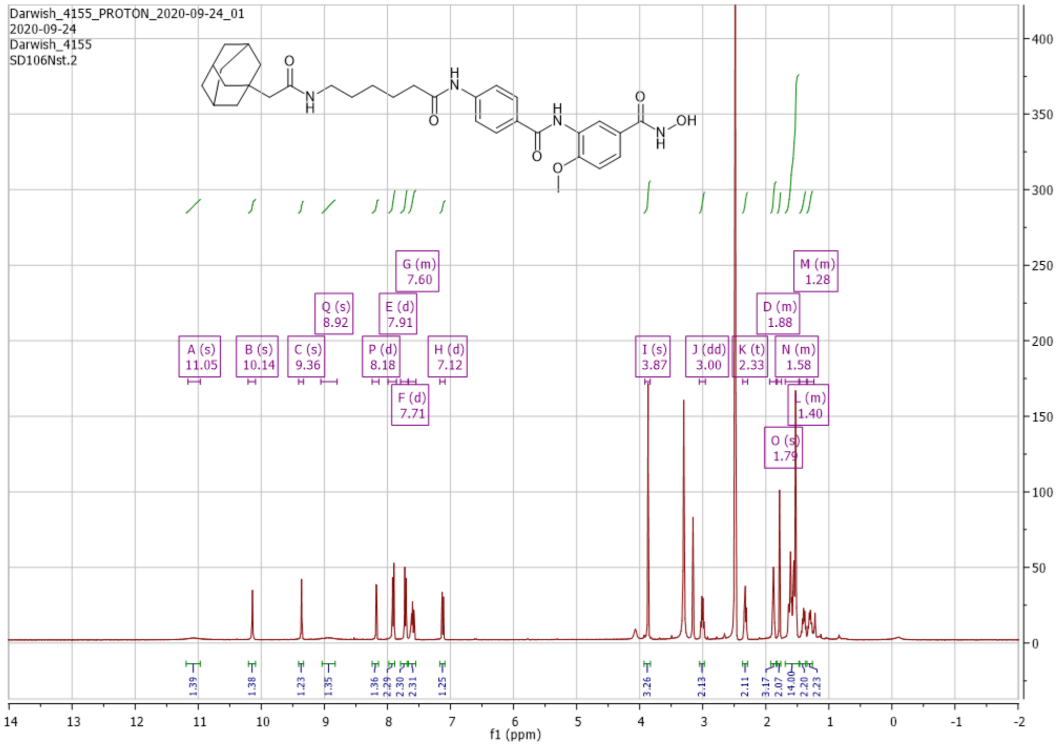

^{13}C NMR (101 MHz, DMSO-d6) δ 172.18, 170.29, 164.96, 154.26, 142.83, 128.84, 128.74, 127.10, 125.11, 124.86, 123.93, 118.76, 118.39, 111.31, 56.47, 50.54, 49.08, 39.23, 38.64, 36.90, 32.57, 29.42, 28.47, 26.58, 25.13.

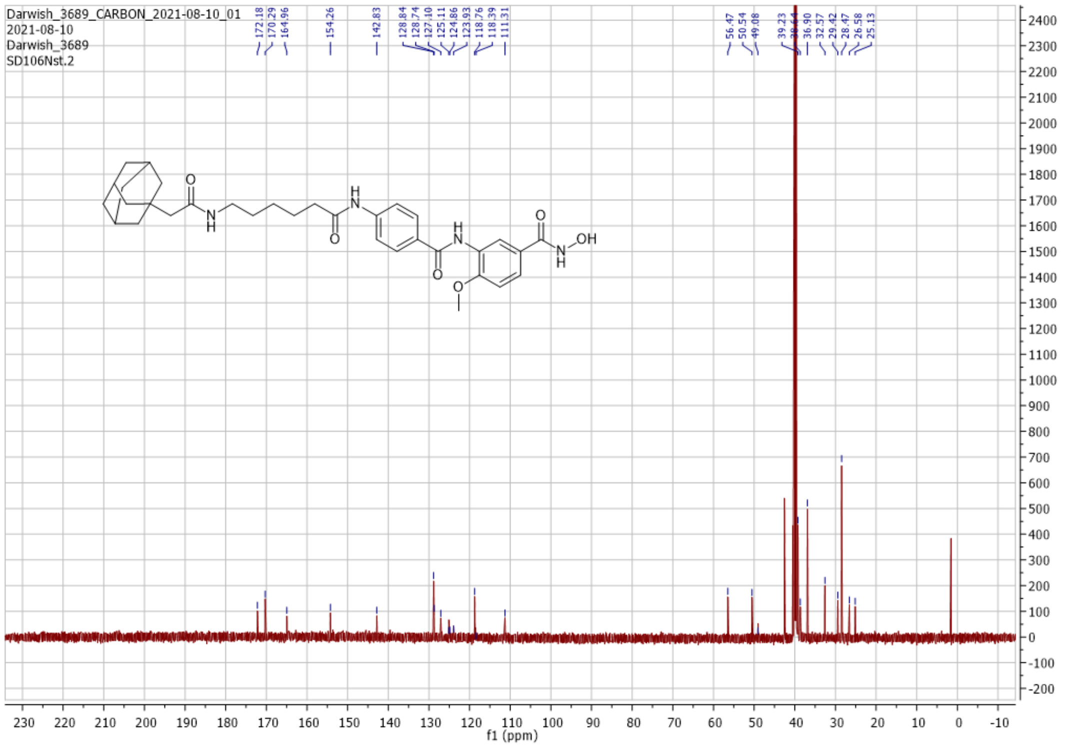

HRMS: 591.319 [M+H]$^+$, calculated $C_{33}H_{43}N_4O_6^+$: 591.318

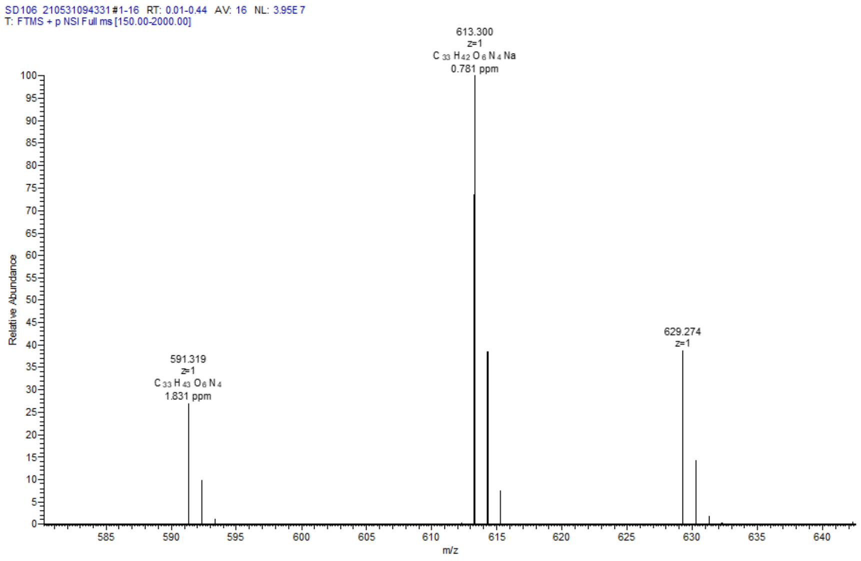

HPLC: (M2) rt. 10.5 min (purity 93 %)

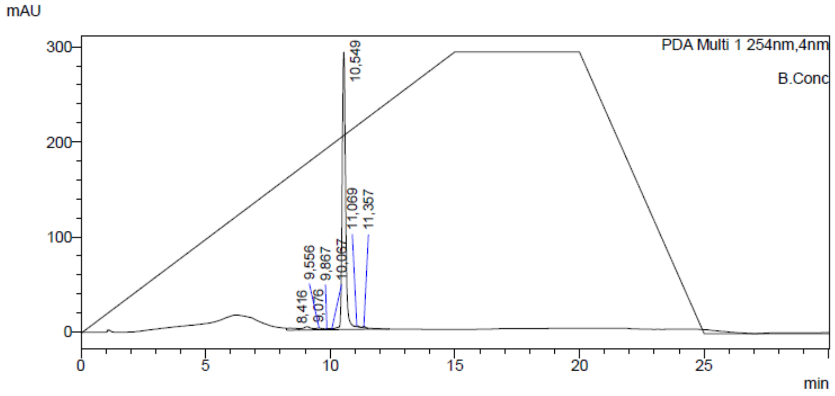

PDA Ch1 254nm

Peak#	Ret. Time	Area	Height	Area%
1	8,416	59092	2244	1,927
2	9,076	77227	3474	2,519
3	9,556	36910	2073	1,204
4	9,867	12519	1121	0,408
5	10,067	13289	1193	0,433
6	10,549	2848965	292058	92,925
7	11,069	7245	843	0,236
8	11,357	10635	1492	0,347
Total		3065882	304498	100,000

3-(4-(8-(2-(Adamantan-1-yl)acetamido)octanamido)benzamido)-N-hydroxy-4-methoxybenzamide (HyT_1n)

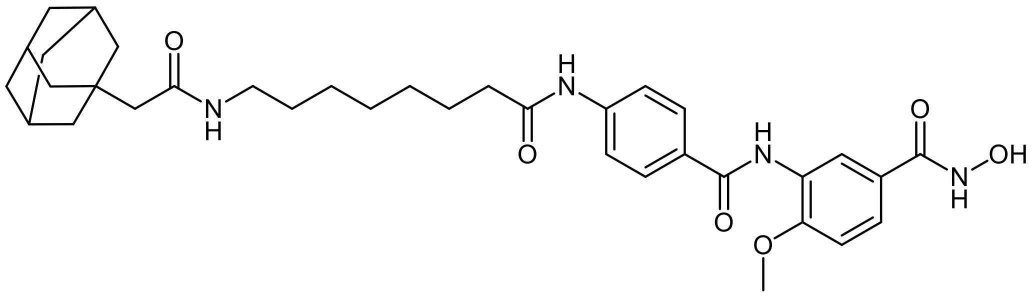

MS m/z: 617.60 [M-H]$^{-}$

^{1}H NMR (400 MHz, DMSO-d6) δ 11.02 (br s, 1H), 10.13 (s, 1H), 9.35 (s, 1H), 8.97 (br s, 1H), 8.17 (d, J = 2.1 Hz, 1H), 7.90 (d, J = 8.8 Hz, 2H), 7.71 (d, J = 8.8 Hz, 2H), 7.58 (dd, J = 8.5, 2.2 Hz, 2H), 7.11 (d, J = 8.7 Hz, 1H), 3.86 (s, 3H), 2.99 (dd, J = 12.6, 6.6 Hz, 2H), 2.32 (t, J = 7.4 Hz, 2H), 1.93 – 1.82 (m, 3H), 1.78 (s, 2H), 1.74 – 1.42 (m, 14H), 1.41 – 1.16 (m, 8H).

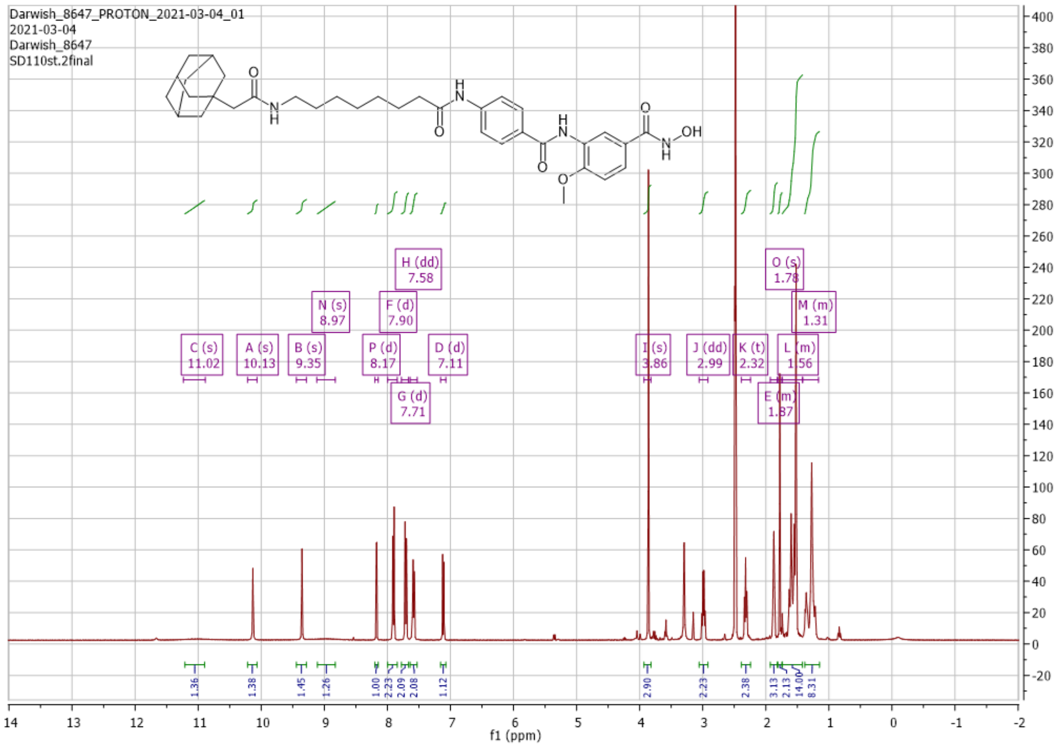

^{13}C NMR (101 MHz, DMSO-d6) δ 172.16, 170.09, 164.89, 154.19, 142.87, 128.85, 128.74, 127.14, 125.22, 124.88, 123.97, 118.71, 118.40, 111.28, 56.48, 50.56, 38.64, 36.92, 32.58, 29.61, 29.10, 28.95, 28.49, 26.77, 25.40.

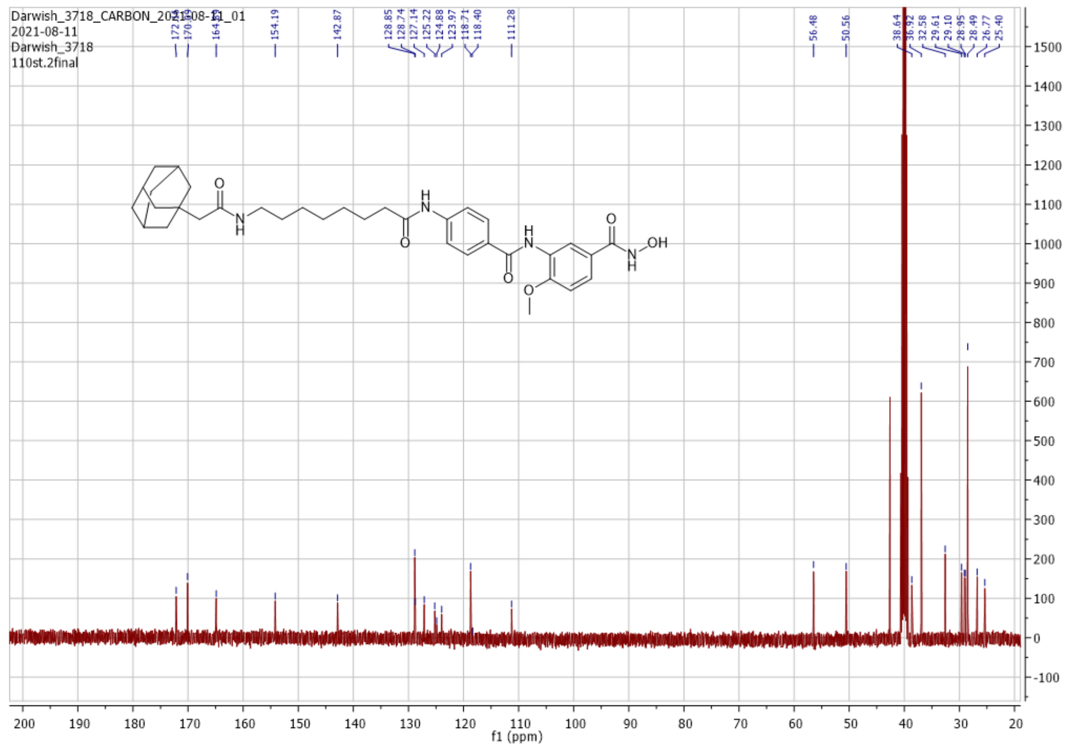

HRMS: 619.350 [M+H]$^+$, calculated $C_{35}H_{47}N_4O_6^+$: 619.350

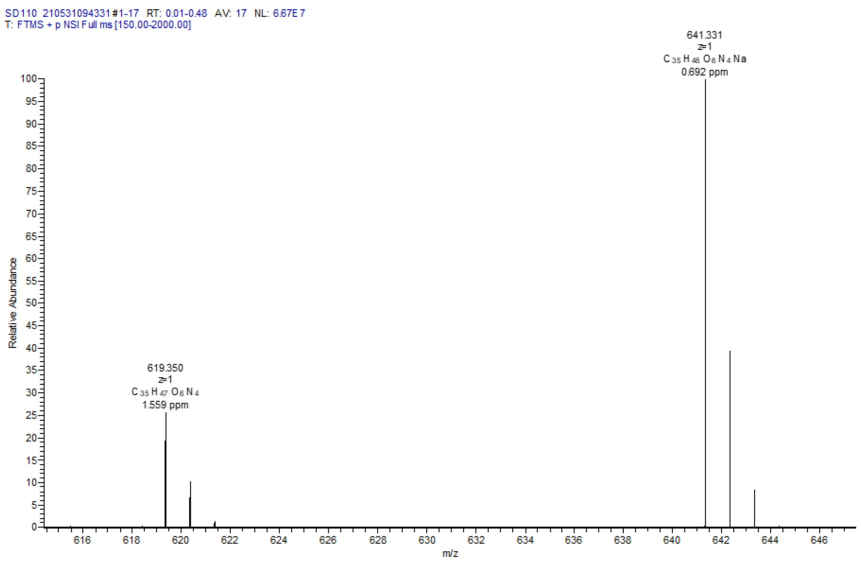

HPLC: (M2) rt. 14.2 min (purity > 99 %)

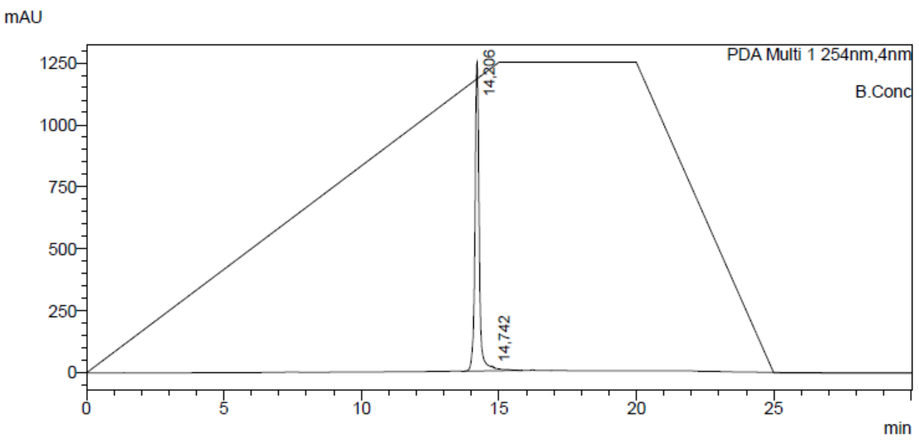

PDA Ch1 254nm

Peak#	Ret. Time	Area	Height	Area%
1	14,206	13493036	1244385	99,860
2	14,742	18864	3609	0,140
Total		13511900	1247993	100,000

N1-((S)-1-((2S,4R)-4-hydroxy-2-((4-(4-methylthiazol-5-yl)benzyl)carbamoyl)pyrrolidin-1-yl)-3,3-dimethyl-1-oxobutan-2-yl)-N8-(4-((5-(hydroxycarbamoyl)-2-methoxyphenyl)carbamoyl)phenyl) octanediamide (VHL_1k)

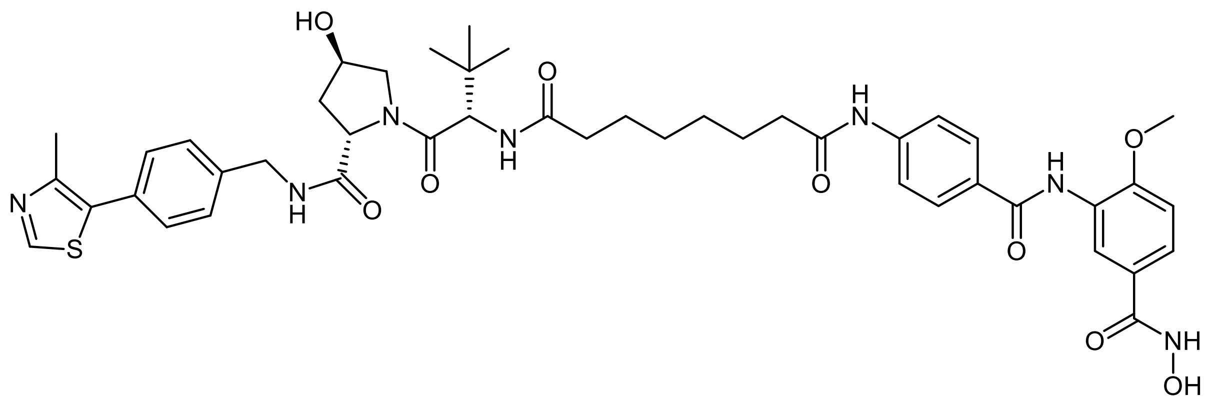

MS m/z: 868.41 $[M-H]^-$

^{1}H NMR (400 MHz, DMSO-d6) δ 11.08 (br s, 2H), 10.14 (s, 1H), 9.37 (s, 1H), 8.96 (s, 1H), 8.53 (t, J = 6.0 Hz, 1H), 8.17 (d, J = 2.1 Hz, 1H), 7.91 (d, J = 8.8 Hz, 2H), 7.82 (d, J = 9.3 Hz, 1H), 7.71 (d, J = 8.8 Hz, 2H), 7.59 (dd, J = 8.6, 2.2 Hz, 1H), 7.46 – 7.29 (m, 4H), 7.12 (d, J = 8.7 Hz, 1H), 5.11 (s, 1H), 4.53 (d, J = 9.4 Hz, 1H), 4.46 – 4.29 (m, 3H), 4.20 (dd, J = 15.9, 5.5 Hz, 1H), 3.86 (s, 3H), 3.70 – 3.52 (m, 2H), 2.43 (s, 3H), 2.29 – 1.94 (m, 4H), 1.64 – 1.39 (m, 4H), 1.34 – 1.20 (m, 7H), 0.92 (s, J = 7.9 Hz, 9H).

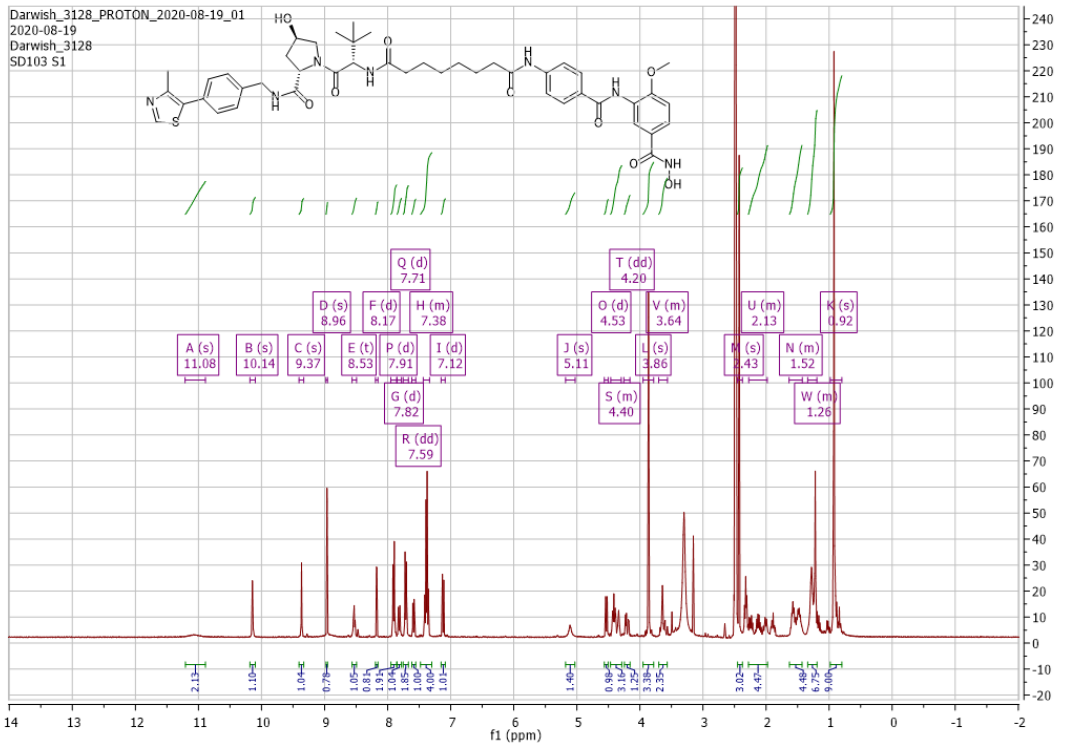

^{13}C NMR (101 MHz, DMSO-d6) δ 173.09, 172.69, 172.68, 172.43, 172.29, 170.17, 164.99, 154.27, 151.89, 148.13, 142.82, 139.88, 131.61, 130.05, 129.07, 128.86, 128.74, 127.86, 127.10, 127.08, 125.07, 118.78, 118.77, 118.52, 111.32, 69.31, 59.15, 56.80, 56.77, 56.47, 49.03, 38.33, 35.63, 35.32, 28.89, 28.84, 26.80, 25.77, 25.35, 16.33.

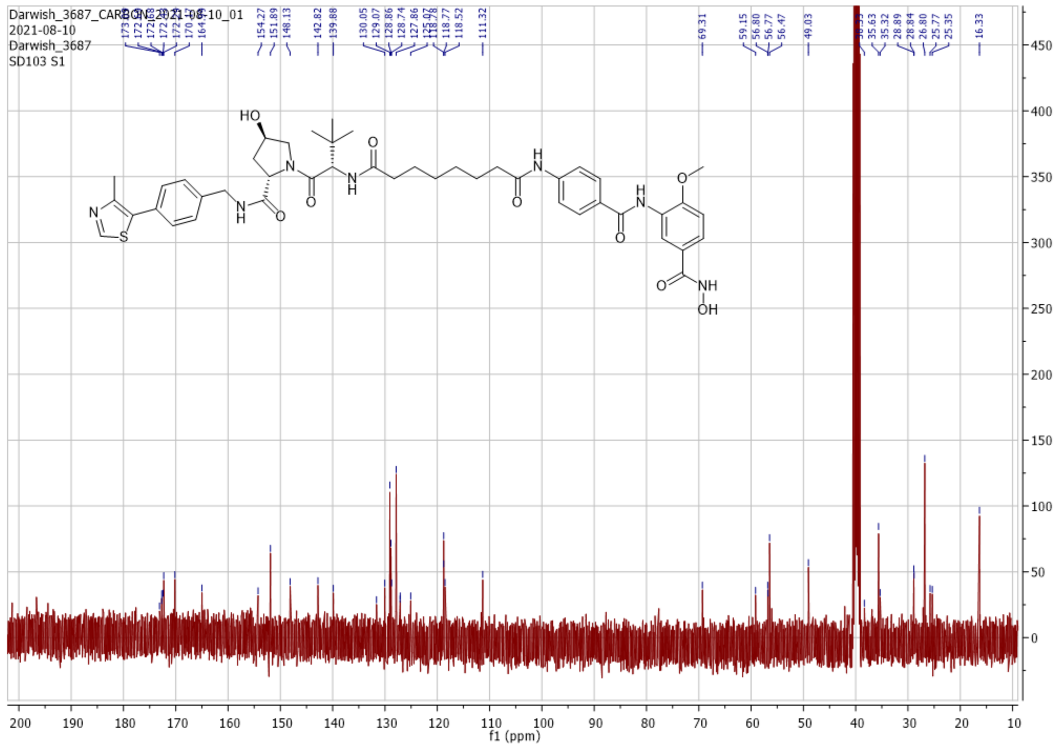

HRMS: 870.388 [M+H]$^+$, calculated $C_{45}H_{56}N_7O_9S^+$ 870.386

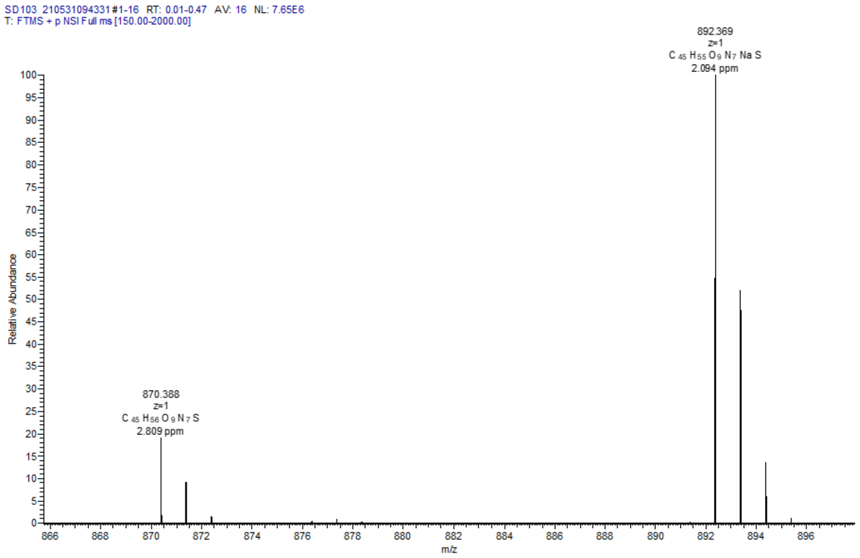

HPLC: (M2) rt. 9.9 min (purity 96%)

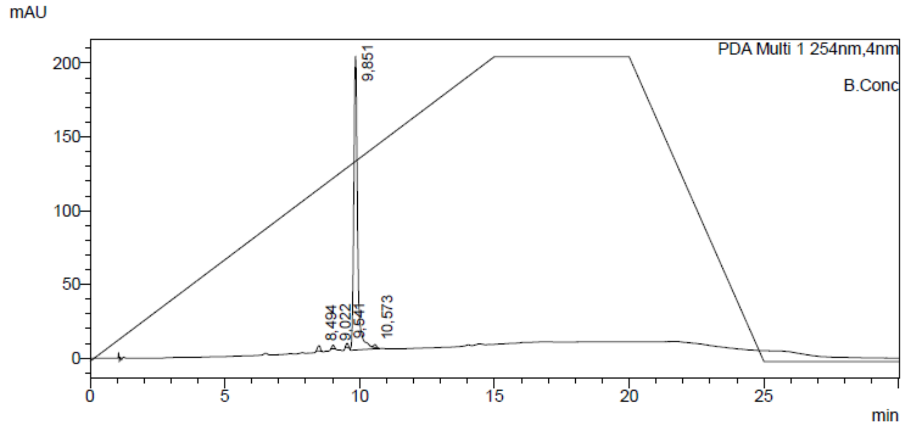

PDA Ch1 254nm

Peak#	Ret. Time	Area	Height	Area%
1	8,494	25574	3638	1,277
2	9,022	17635	2707	0,880
3	9,541	16248	2945	0,811
4	9,851	1929484	198726	96,319
5	10,573	14281	2073	0,713
Total		2003222	210090	100,000

3-(4-((8-((2-(2,6-Dioxopiperidin-3-yl)-1,3-dioxoisoindolin-4-yl)amino)octanamido)methyl)benzamido)-N-hydroxy-4-methoxybenzamide (CRBN_1c)

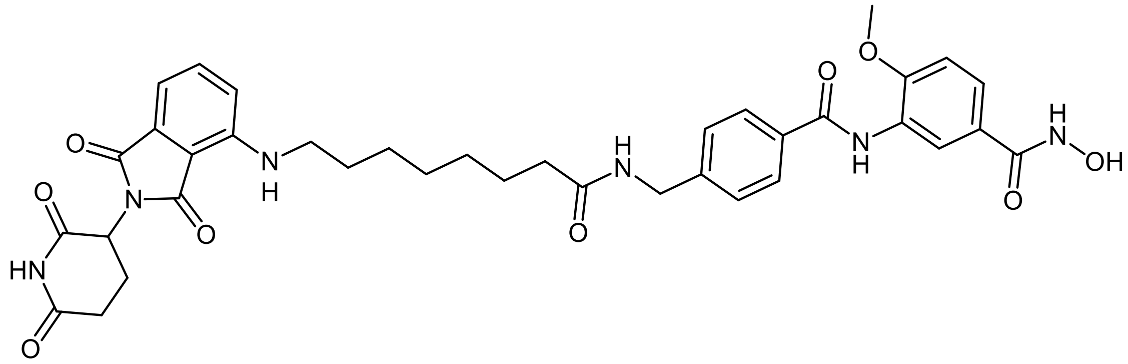

MS m/z: 711.36 [M-H]$^-$

^{1}H NMR (400 MHz, DMSO-d6) δ 11.04 (br s, 2H), 9.45 (s, 1H), 8.93 (br s, 1H), 8.35 (t, J = 5.9 Hz, 1H), 8.17 (d, J = 2.1 Hz, 1H), 7.90 (d, J = 8.2 Hz, 2H), 7.66 – 7.50 (m, 2H), 7.35 (d, J = 8.2 Hz, 2H), 7.12 (d, J = 8.7 Hz, 1H), 7.07 (d, J = 8.6 Hz, 1H), 6.99 (d, J = 7.0 Hz, 1H), 6.50 (t, J = 5.7 Hz, 1H), 5.02 (dd, J = 12.8, 5.4 Hz, 1H), 4.31 (d, J = 5.8 Hz, 2H), 3.85 (s, 3H), 3.00 – 2.73 (m, 2H), 2.69 – 2.51 (m, 2H), 2.14 (t, J = 7.4 Hz, 2H), 2.07 – 1.86 (m, 2H), 1.67 – 1.40 (m, 4H), 1.40 – 1.14 (m, 6H).

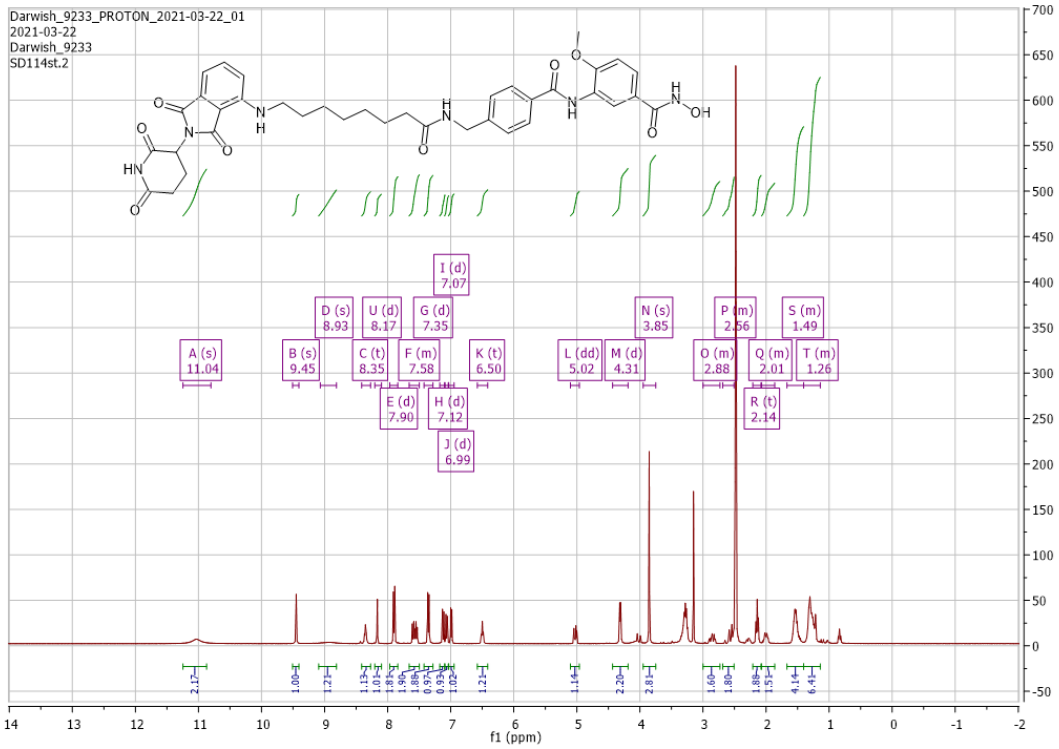

^{13}C NMR (101 MHz, DMSO-d6) δ 173.22, 172.71, 170.52, 169.39, 167.73, 165.30, 154.32, 146.87, 144.20, 136.71, 133.15, 132.63, 127.98, 127.54, 127.01, 125.17, 125.06, 124.14, 117.61, 111.34, 110.81, 109.46, 56.48, 49.04, 48.99, 42.27, 35.76, 31.42, 29.08, 28.92, 26.67, 25.68, 22.59.

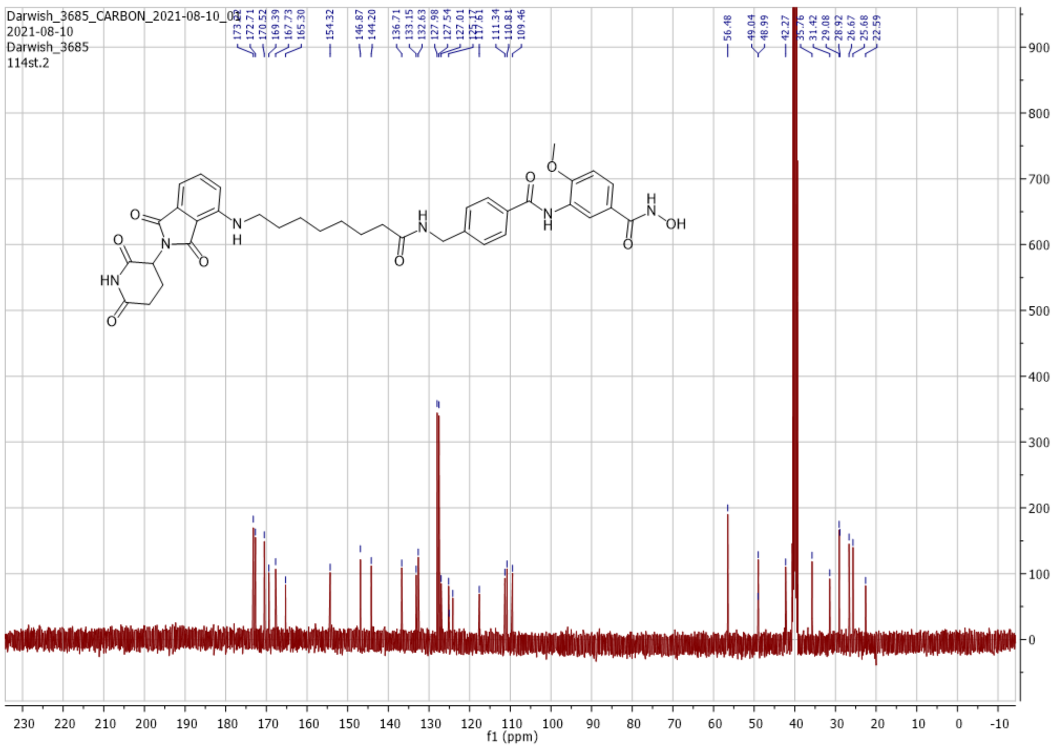

HRMS: 713.295 [M+H]+, calculated $C_{37}H_{41}N_6O_9^+$: 713.294

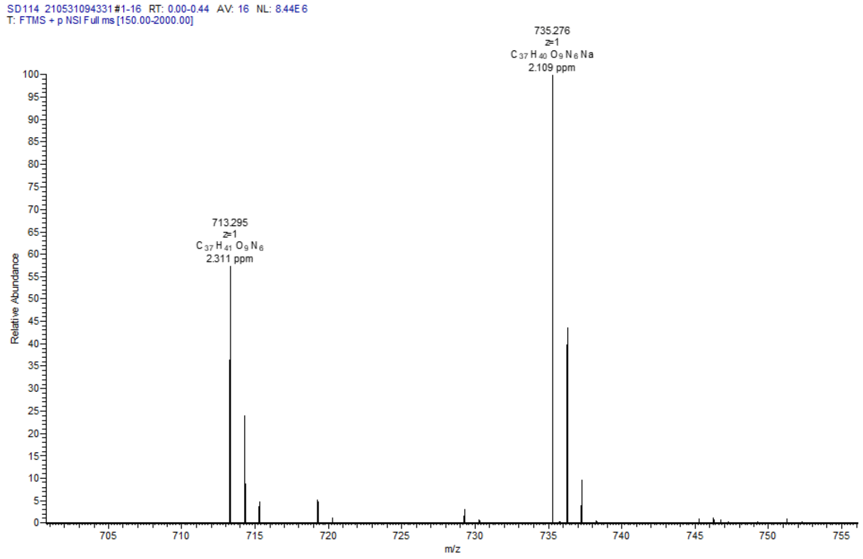

HPLC: (M2) rt. 12.2 min (purity >99 %)

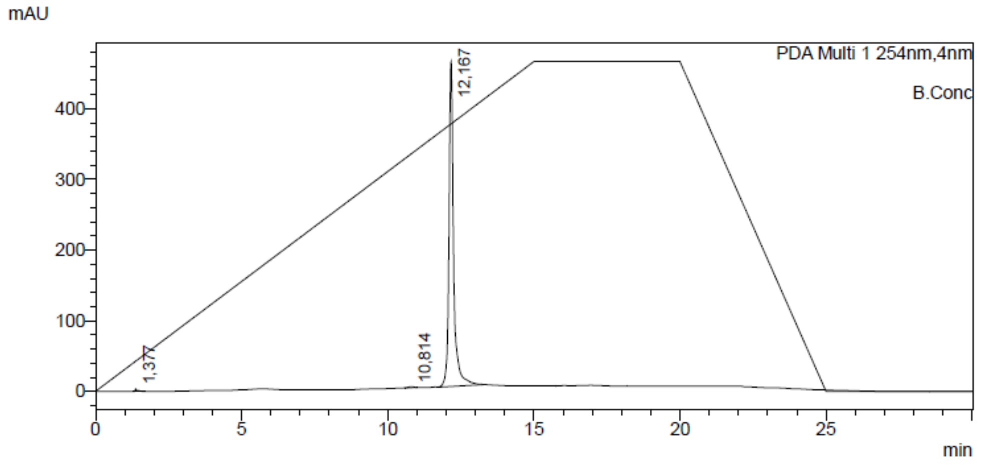

PDA Ch1 254nm

Peak#	Ret. Time	Area	Height	Area%
1	1,377	16626	3596	0,324
2	10,814	19273	1551	0,376
3	12,167	5092210	459689	99,300
Total		5128109	464836	100,000

3-(4-((8-(2-(Adamantan-1-yl)acetamido)octanamido)methyl)benzamido)-N-hydroxy-4-methoxybenzamide (HyT_1o)

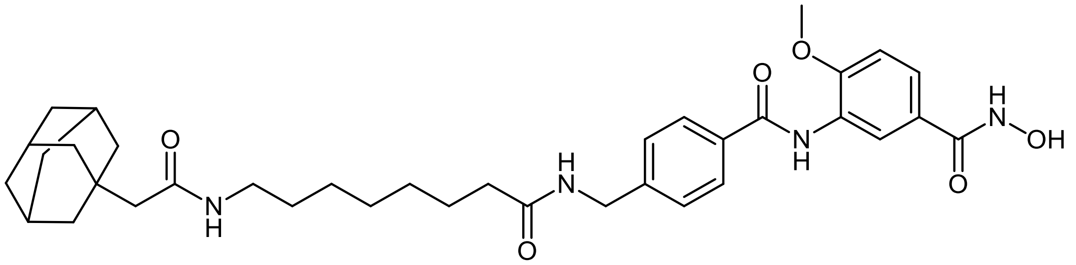

MS m/z: 631.54 [M-H]$^-$

^{1}H NMR (400 MHz, DMSO-d6) δ 10.82 (br s, 1H), 9.45 (s, 1H), 9.09 (br s, 1H), 8.36 (t, J = 5.9 Hz, 1H), 8.16 (d, J = 2.1 Hz, 1H), 7.90 (d, J = 8.3 Hz, 2H), 7.70 – 7.51 (m, 2H), 7.35 (d, J = 8.3 Hz, 2H), 7.12 (d, J = 8.7 Hz, 1H), 4.31 (d, J = 5.9 Hz, 2H), 3.85 (s, 3H), 2.99 (dd, J = 12.6, 6.7 Hz, 2H), 2.13 (t, J = 7.4 Hz, 2H), 1.94 – 1.83 (m, 3H), 1.78 (s, 2H), 1.73 – 1.42 (m, 12H), 1.43 – 1.30 (m, 2H), 1.29 – 1.12 (m,8H).

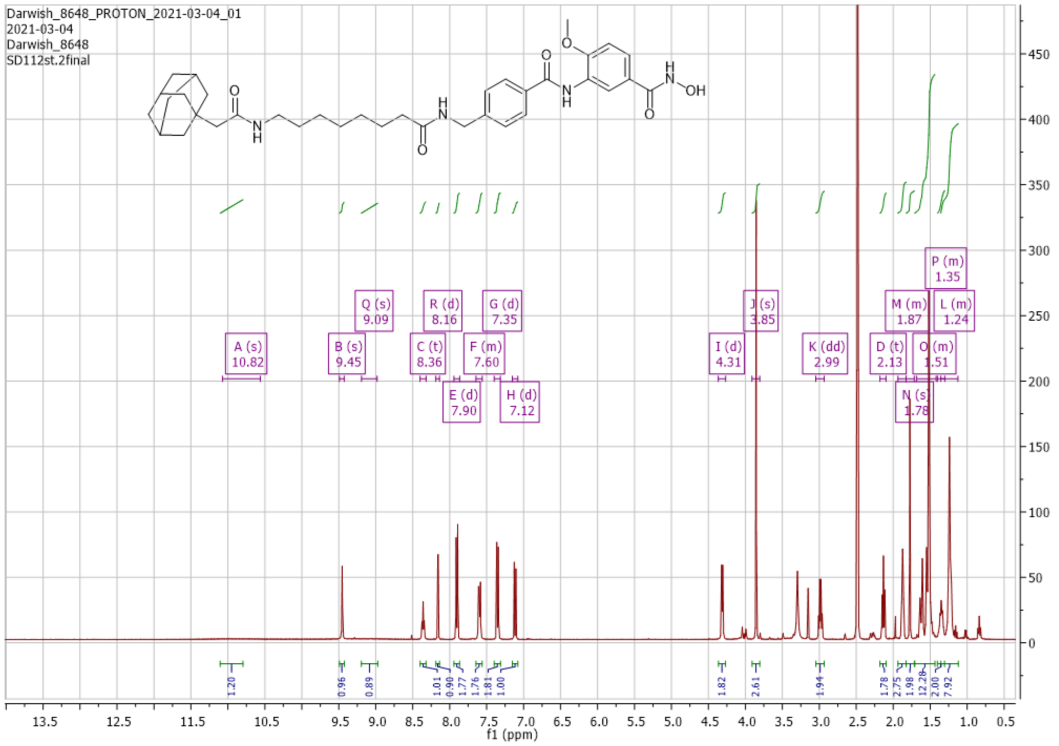

^{13}C NMR (101 MHz, DMSO-d6) δ 172.68, 170.10, 165.29, 154.31, 144.19, 133.16, 127.98, 127.76, 127.55, 127.51, 127.00, 125.27, 124.18, 111.33, 56.48, 50.54, 42.59, 36.92, 35.78, 32.58, 29.62, 29.13, 28.90, 28.49, 26.80, 25.68.

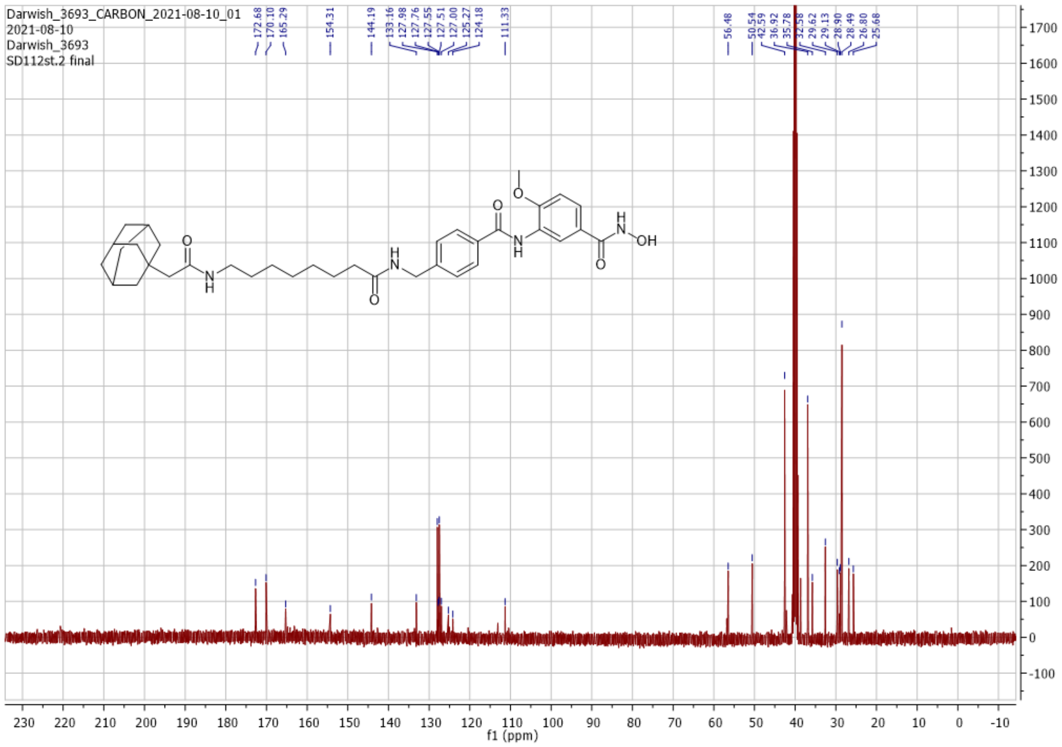

HRMS: 633.366 [M+H]+, calculated $C_{36}H_{49}N_4O_6^+$: 633.365

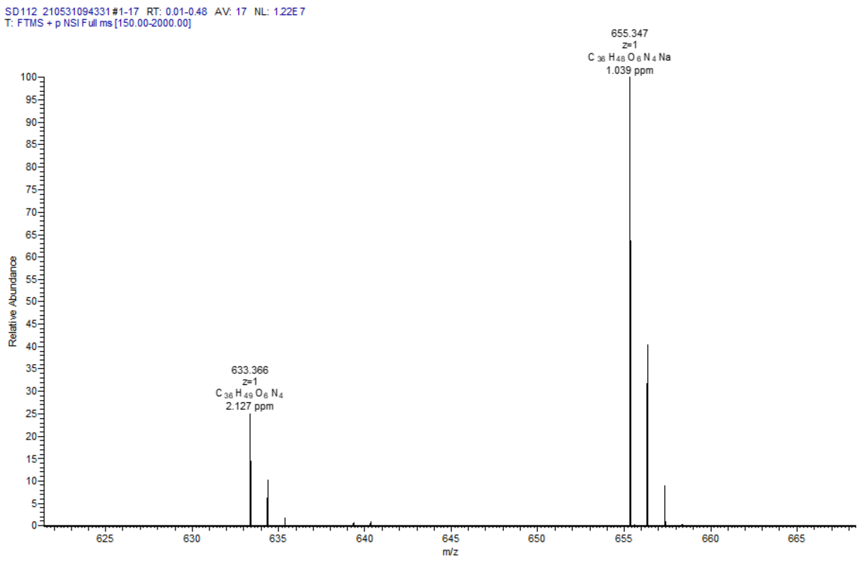

HPLC: (M2) rt. 13.8 min (purity 99%)

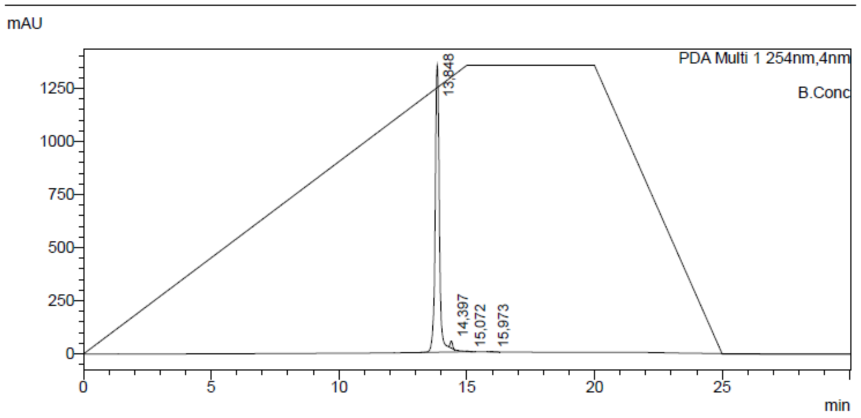

PDA Ch1 254nm

Peak#	Ret. Time	Area	Height	Area%
1	13,848	15804193	1349191	98,526
2	14,397	209370	33151	1,305
3	15,072	2588	517	0,016
4	15,973	24530	2480	0,153
Total		16040681	1385339	100,000

3-((4-((1-(6-((2-(2,6-Dioxopiperidin-3-yl)-1,3-dioxoisoindolin-4-yl)amino)-6-oxohexyl)-1H-1,2,3-triazol-4-yl)methoxy)benzyl)amino)-N-hydroxy-4-methylbenzamide (CRBN_1d)

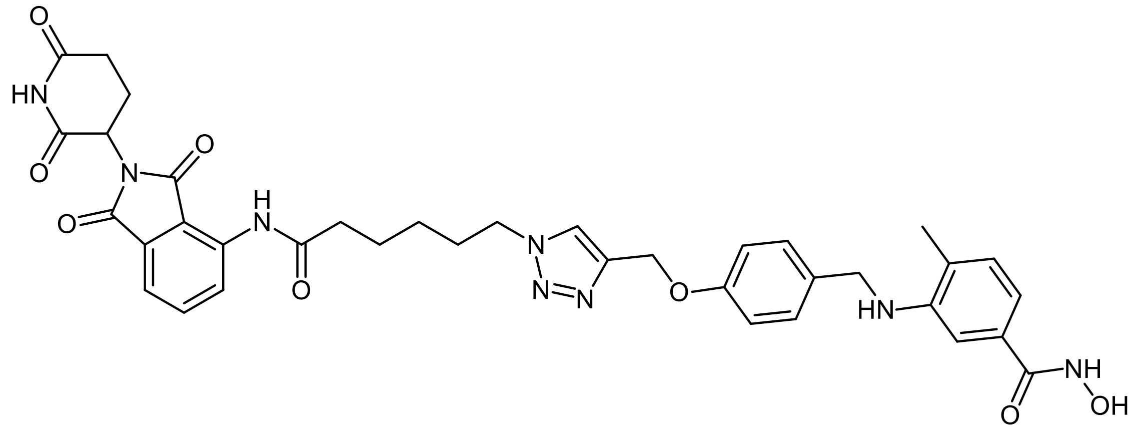

MS m/z: 721.67 [M-H]$^-$

^{1}H NMR (400 MHz, DMSO-d6) δ 11.11 (s, 1H), 10.88 (s, 1H), 9.67 (s, 1H), 8.75 (d, J = 1.7 Hz, 1H), 8.44 (d, J = 8.5 Hz, 1H), 8.18 (s, 1H), 7.83 – 7.77 (m, 1H), 7.59 (d, J = 7.2 Hz, 1H), 7.26 (d, J = 8.5 Hz, 2H), 7.00 – 6.92 (m, 3H), 6.86 – 6.80 (m, 2H), 5.61 (t, J = 5.9 Hz, 1H), 5.12 (dd, J = 12.7, 5.3 Hz, 1H), 5.06 (s, 2H), 4.38 – 4.27 (m, 4H), 2.92 – 2.81 (m, 1H), 2.63 – 2.51 (m, 2H), 2.45 – 2.41 (m, 1H), 2.14 (s, 3H), 1.89 – 1.78 (m, 2H), 1.69 – 1.58 (m, 2H), 1.57 – 1.46 (m, 2H), 1.33 – 1.26 (m, 2H).

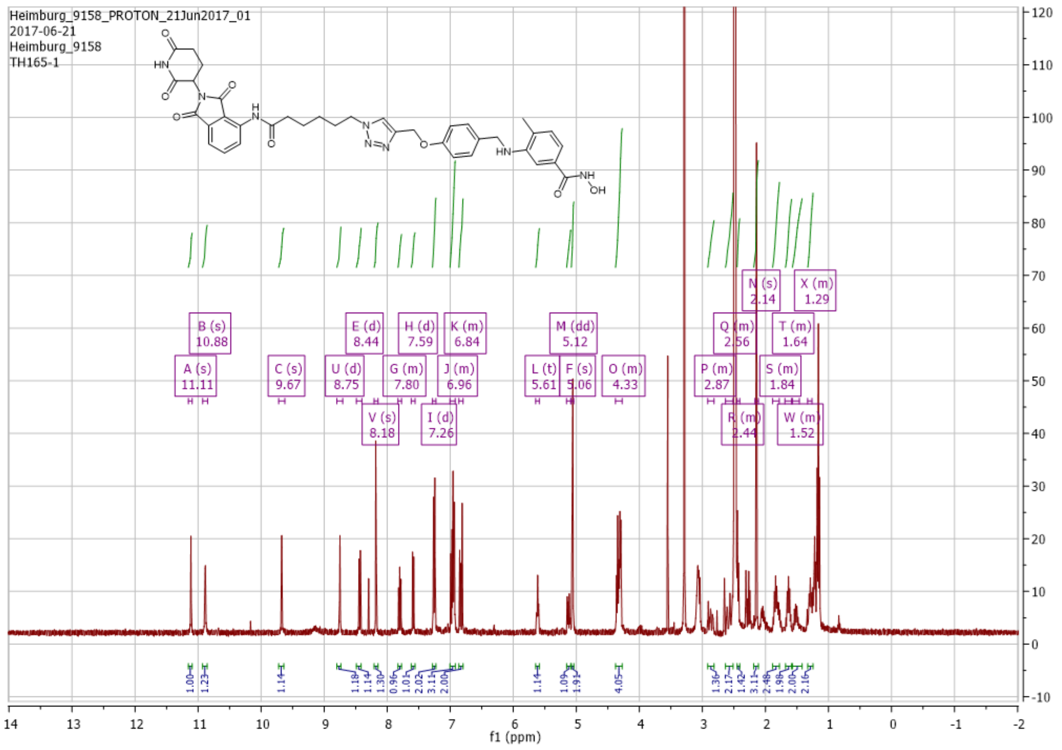

^{13}C NMR (101 MHz, cd$_3$od) δ 172.46, 172.32, 169.06, 168.96, 166.88, 157.41, 146.20, 143.85, 137.12, 136.23, 131.91, 131.21, 130.44, 129.87, 128.76, 126.17, 125.23, 123.31, 118.50, 115.28, 114.91, 108.24, 77.50, 61.64, 50.09, 49.28, 37.12, 31.18, 29.72, 25.71, 24.29, 22.49, 17.23.

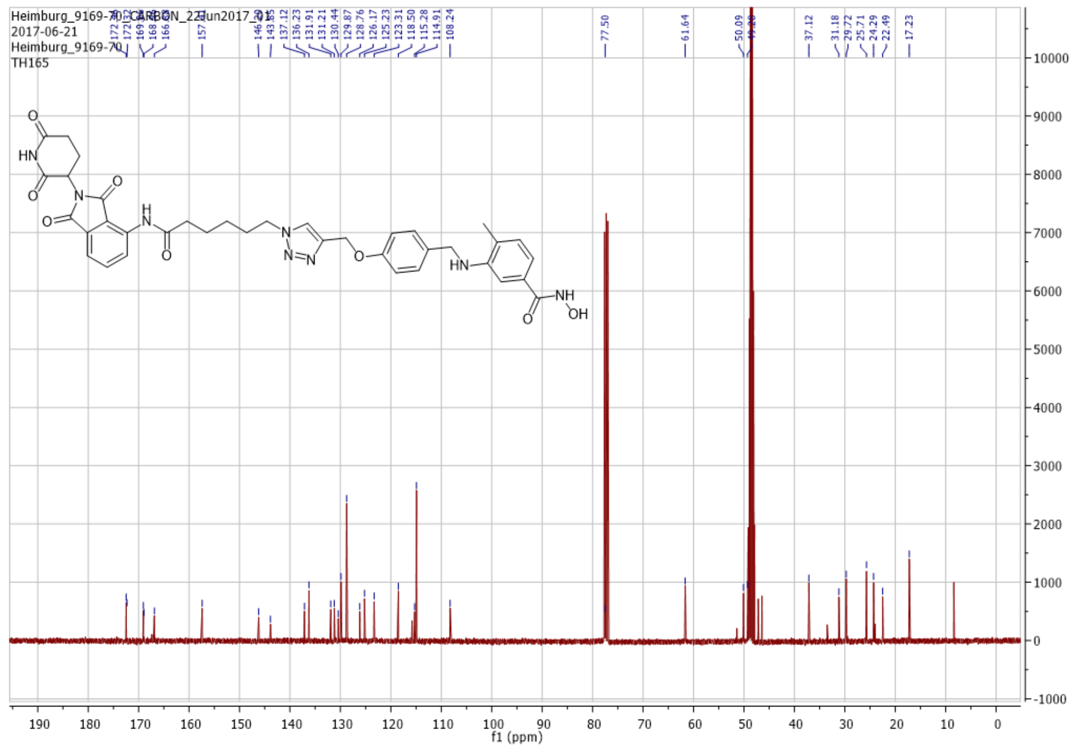

HRMS: 723.290 [M+H]+, calculated $C_{37}H_{39}N_8O_8^+$ 723.289

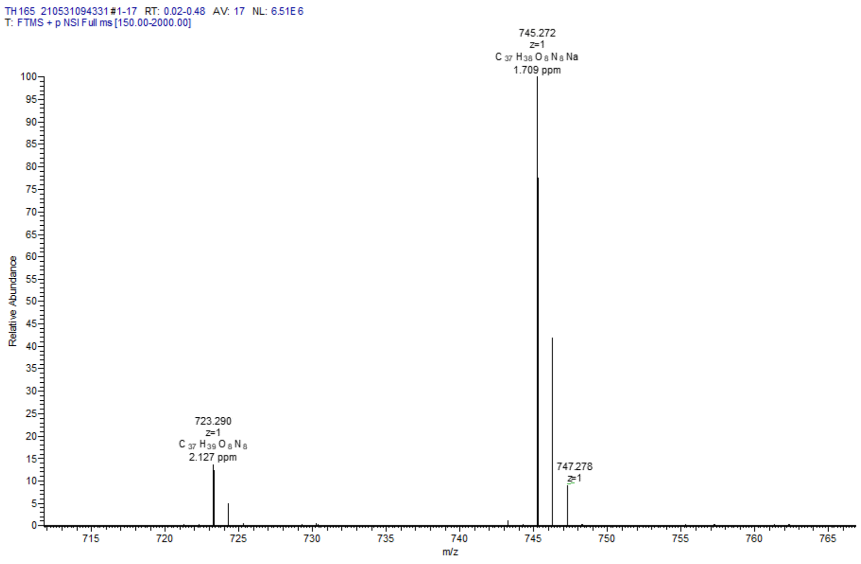

HPLC: (M2) rt: 7.3 min (purity 96 %)

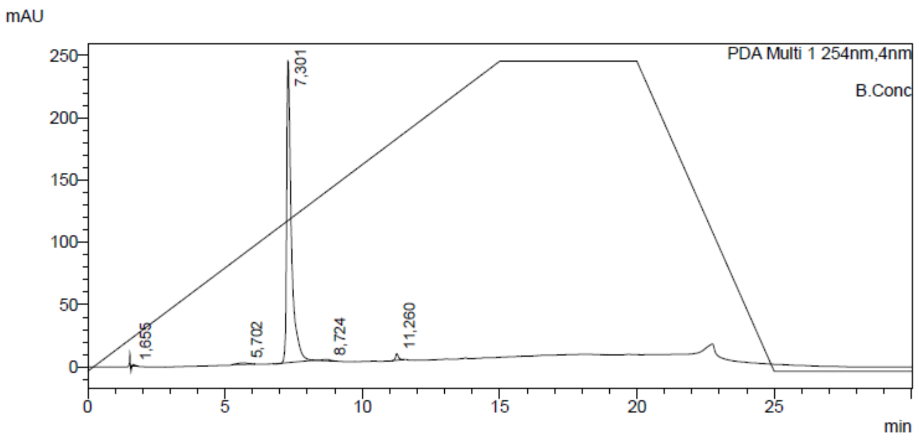

PDA Ch1 254nm

Peak#	Ret. Time	Area	Height	Area%
1	1,655	10831	1434	0,360
2	5,702	34375	1358	1,143
3	7,301	2900018	241784	96,400
4	8,724	22581	1188	0,751
5	11,260	40496	5279	1,346
Total		3008302	251042	100,000

3-((4-((1-(6-((2-(2,6-Dioxopiperidin-3-yl)-1,3-dioxoisoindolin-4-yl)amino)-6-oxohexyl)-1H-1,2,3-triazol-4-yl)methoxy)benzyl)amino)-N-hydroxy-4-methoxybenzamide (CRBN_1e)

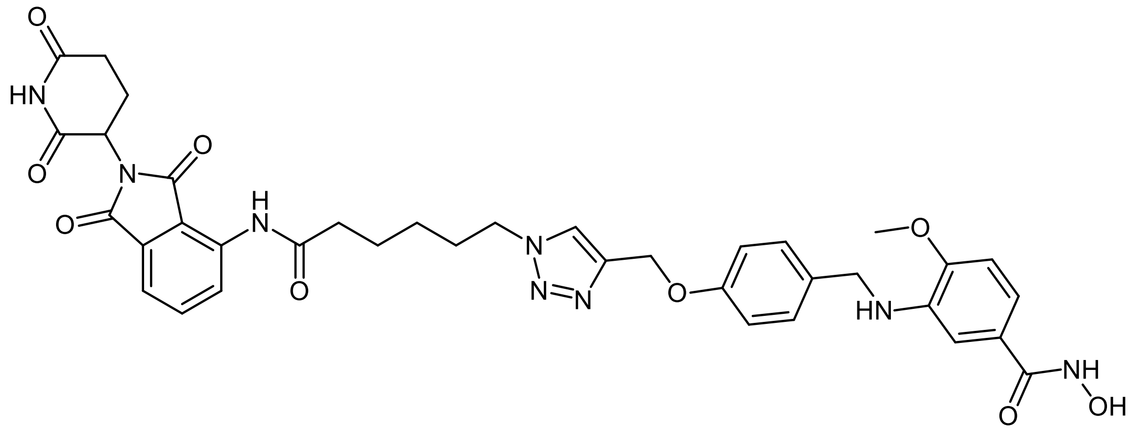

MS m/z: 737.62 $[M-H]^-$

^{1}H NMR (400 MHz, DMSO-d6) δ 11.10 (br s, 1H), 10.87 (br s, 1H), 9.67 (s, 1H), 8.72 (s, 1H), 8.44 (d, J = 8.4 Hz, 1H), 8.18 (s, 1H), 7.80 (t, J = 7.9 Hz, 1H), 7.59 (d, J = 7.3 Hz, 1H), 7.24 (d, J = 8.5 Hz, 2H), 6.98 (d, J = 1.6 Hz, 1H), 6.94 (d, J = 8.8 Hz, 2H), 6.86 (d, J = 1.3 Hz, 1H), 6.81 (d, J = 8.3 Hz, 1H), 5.48 (t, J = 6.0 Hz, 1H), 5.12 (dd, J = 12.7, 5.4 Hz, 1H), 5.06 (s, 2H), 4.34 (t, J = 7.1 Hz, 2H), 4.26 (d, J = 6.1 Hz, 2H), 3.81 (s, 3H), 2.67 – 2.51 (m, 2H), 2.46 – 2.34 (m, 2H), 2.13 – 2.00 (m, 1H), 1.91 – 1.78 (m, 2H), 1.70 – 1.58 (m, 2H), 1.53 – 1.40 (m, 1H), 1.37 – 1.25 (m, 2H).

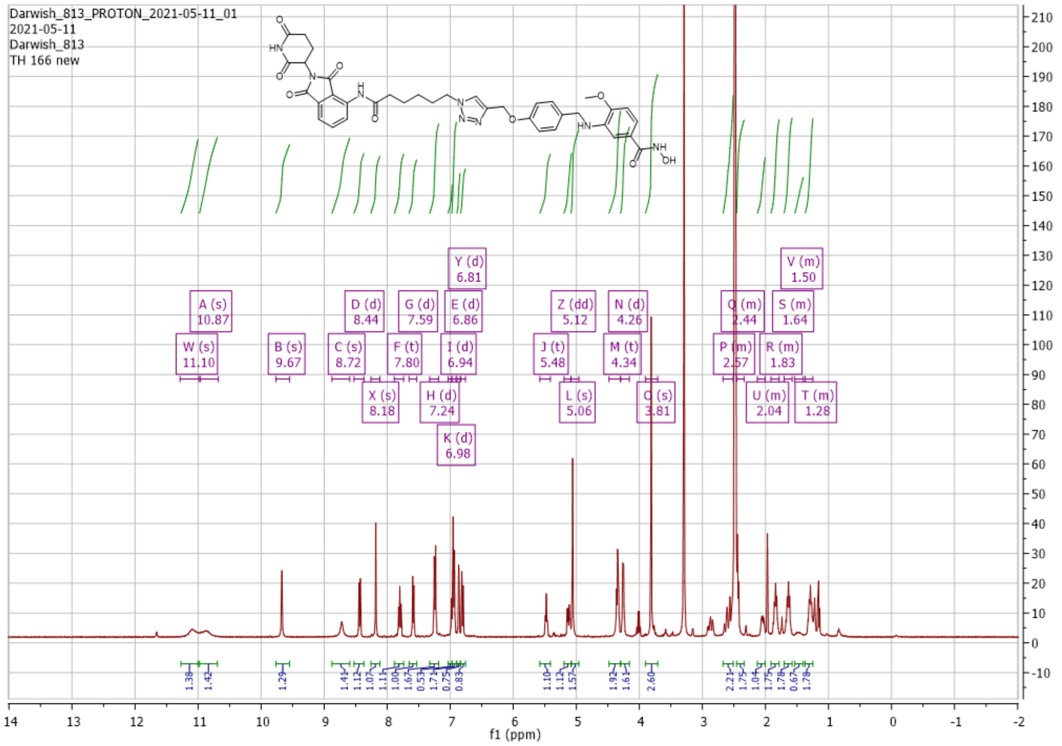

^{13}C NMR (101 MHz, DMSO-d6) δ 173.17, 172.30, 170.20, 168.08, 167.09, 157.38, 149.04, 143.13, 137.88, 136.94, 136.51, 132.64, 131.90, 128.72, 126.78, 125.74, 124.73, 118.75, 117.48, 114.97, 109.33, 61.61, 55.99, 49.66, 49.36, 45.93, 36.62, 31.37, 29.85, 25.78, 24.55, 22.43.

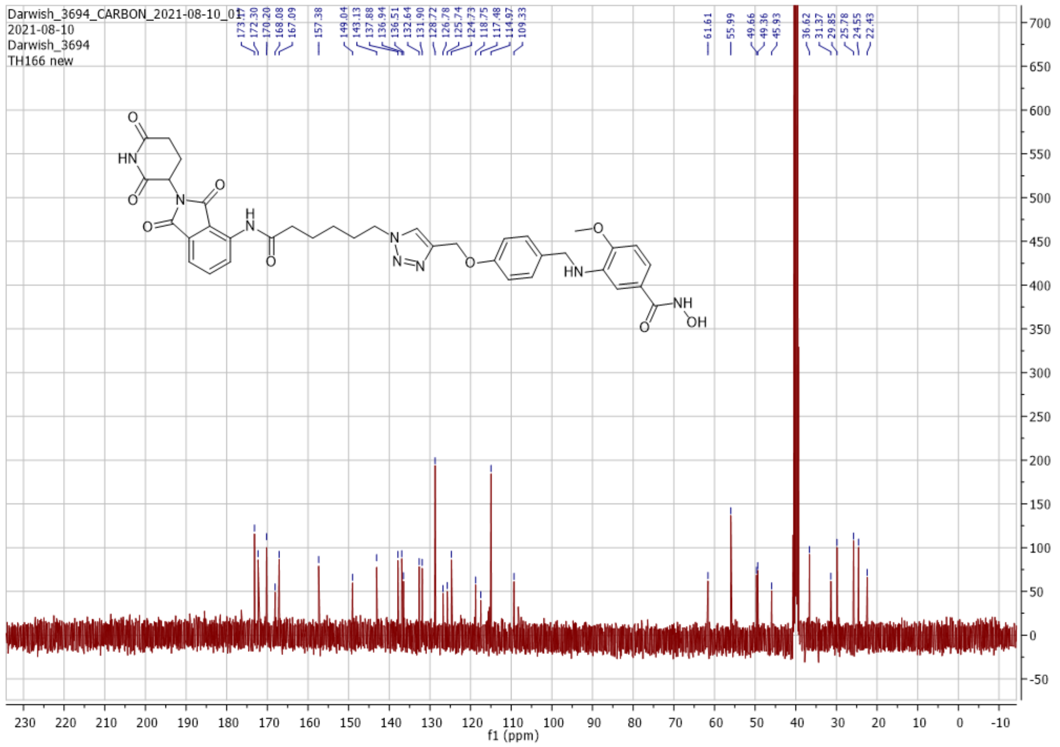

HRMS: 739.284 [M+H]+, calculated $C_{37}H_{39}N_8O_9^+$ 739.284

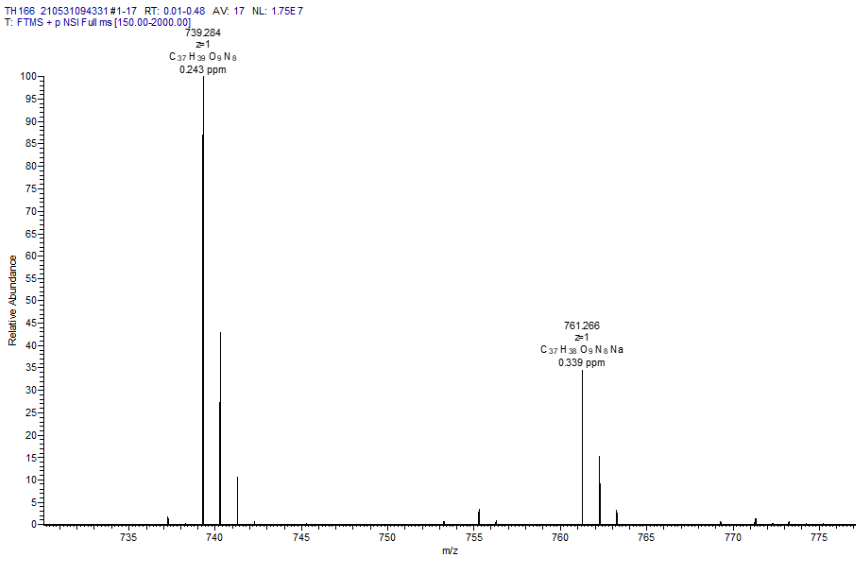

HPLC: (M2) rt: 10.9 min (purity 99 %)

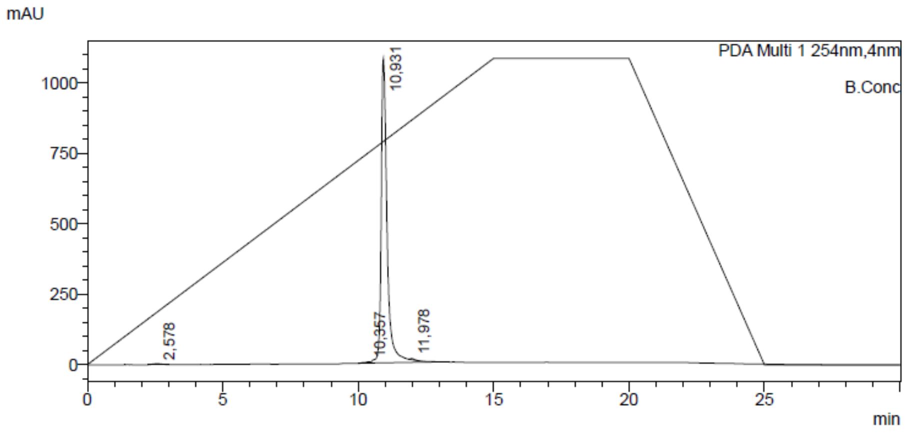

PDA Ch1 254nm

Peak#	Ret. Time	Area	Height	Area%
1	2,578	69305	3515	0,417
2	10,357	65504	4334	0,394
3	10,931	16468557	1080715	99,000
4	11,978	31616	3759	0,190
Total		16634982	1092323	100,000

4-Chloro-3-((4-((1-(6-((2-(2,6-dioxopiperidin-3-yl)-1,3-dioxoisoindolin-4-yl)amino)-6-oxohexyl)-1H-1,2,3-triazol-4-yl)methoxy)benzyl)amino)-N-hydroxybenzamide (CRBN_1f)

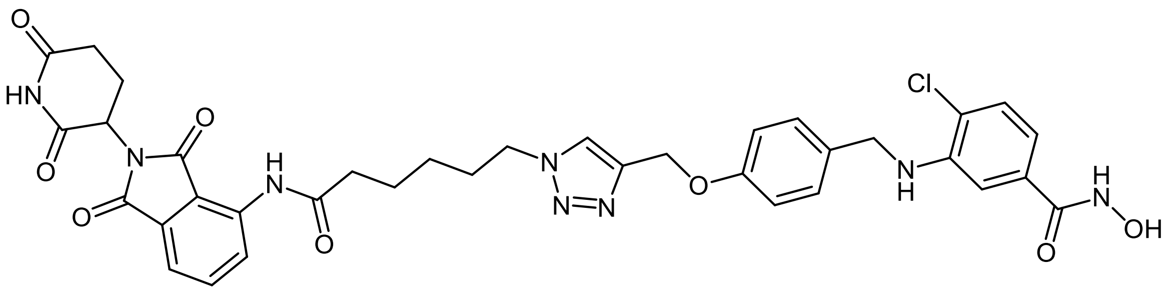

MS m/z: 741.59 $[M-H]^-$

^{1}H NMR (400 MHz, DMSO-d6) δ 11.11 (br s, 2H), 9.67 (s, 1H), 8.94 (br s, 1H), 8.44 (d, J = 8.4 Hz, 1H), 8.18 (s, 1H), 7.84 – 7.75 (m, 1H), 7.58 (d, J = 7.2 Hz, 1H), 7.32 – 7.21 (m, 3H), 7.00 – 6.93 (m, 3H), 6.90 (dd, J = 8.2, 1.8 Hz, 1H), 6.13 (t, J = 5.9 Hz, 1H), 5.12 (dd, J = 12.8, 5.4 Hz, 1H), 5.06 (s, 2H), 4.41 – 4.29 (m, 4H), 2.65 – 2.50 (m, 2H), 2.47 – 2.37 (m, 2H), 2.30 – 2.20 (m, 1H), 1.90 – 1.75 (m, 2H), 1.70 – 1.59 (m, 2H), 1.56 – 1.46 (m, 1H), 1.36 – 1.24 (m, 2H).

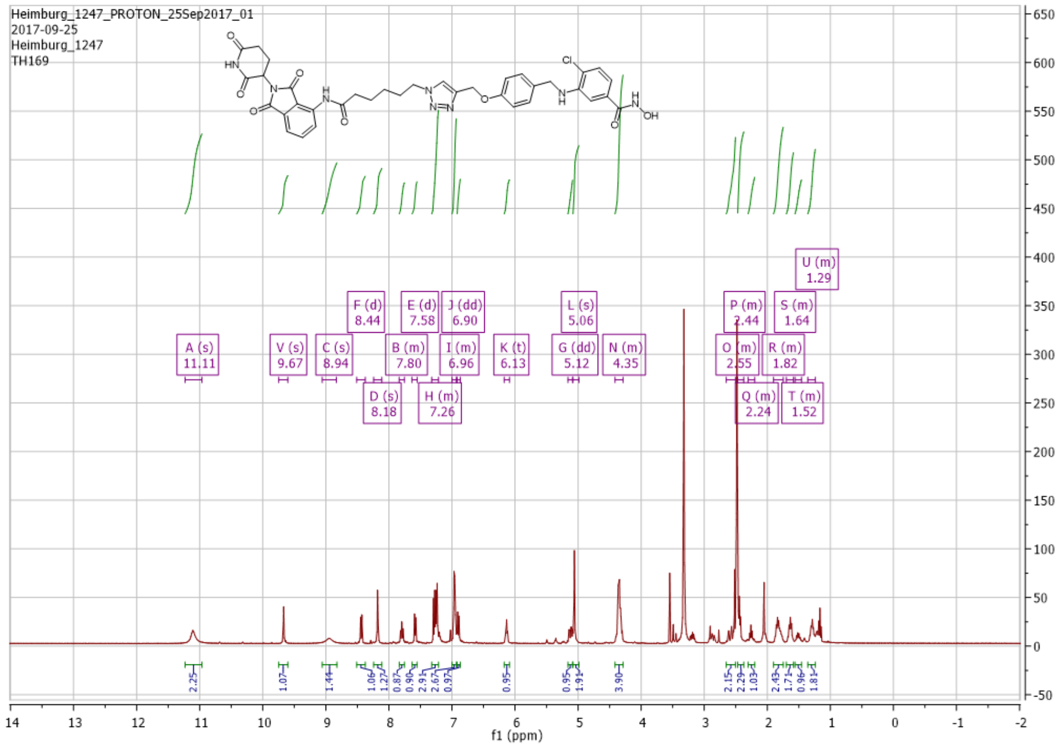

^{13}C NMR (101 MHz, DMSO-d6) δ 173.17, 172.30, 170.20, 168.09, 167.09, 157.44, 144.23, 143.10, 136.94, 136.51, 132.82, 132.00, 131.90, 129.25, 128.49, 126.79, 124.74, 120.84, 118.75, 117.48, 115.05, 110.31, 61.60, 49.66, 49.36, 45.70, 36.63, 31.37, 29.85, 25.79, 24.55, 22.44.

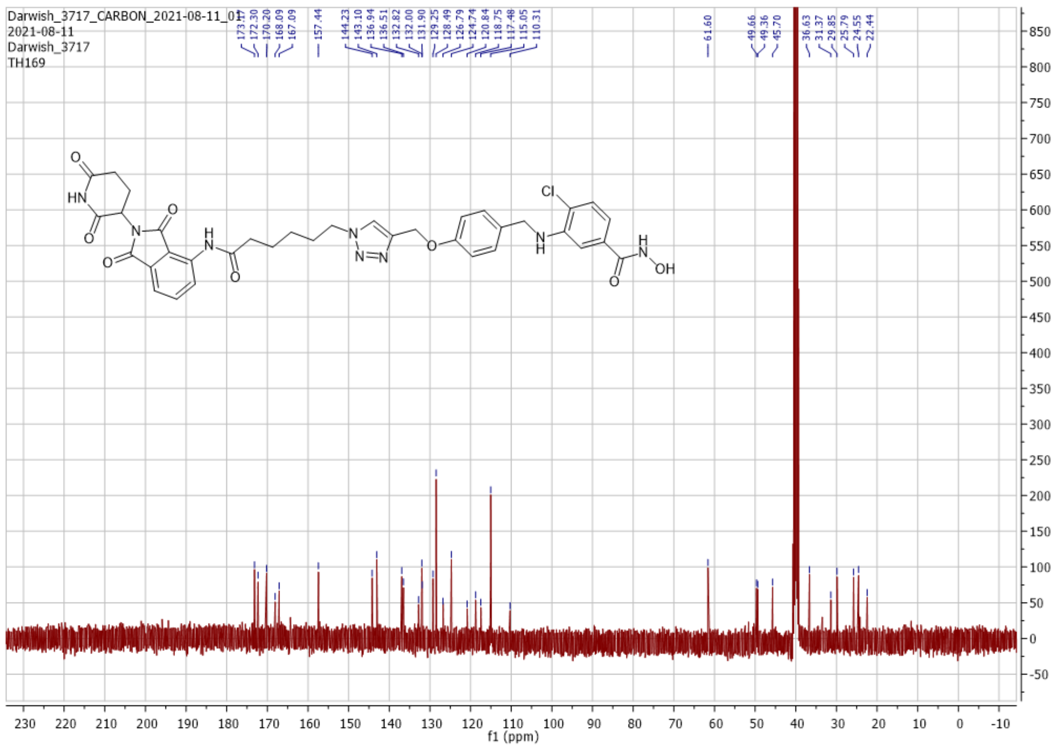

HRMS: 743.235 [M+H]$^+$, calculated $C_{36}H_{36}ClN_8O_8^+$ 743.234

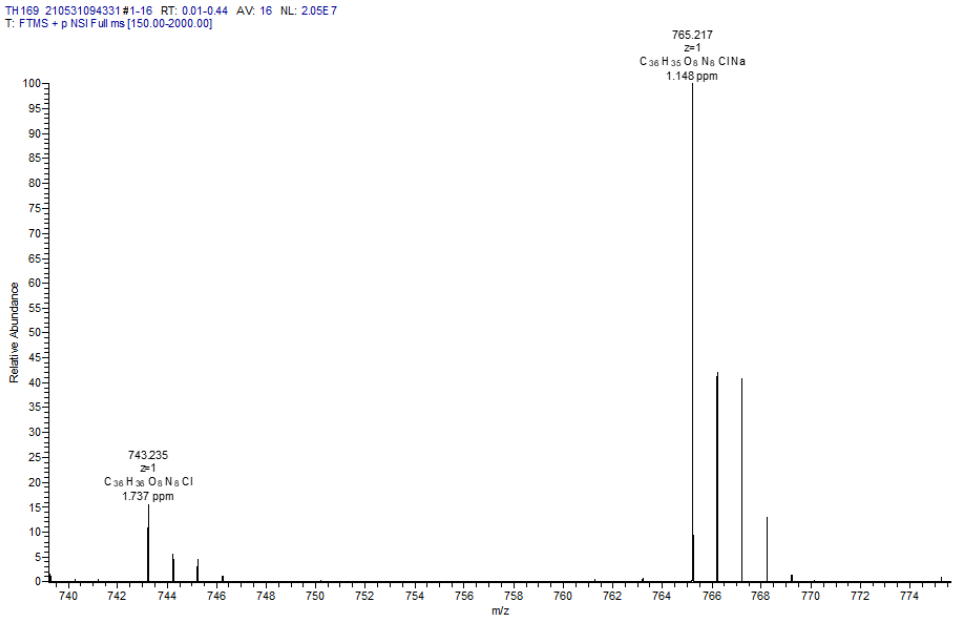

HPLC: (M1) rt: 12.9 min (purity 97 %)

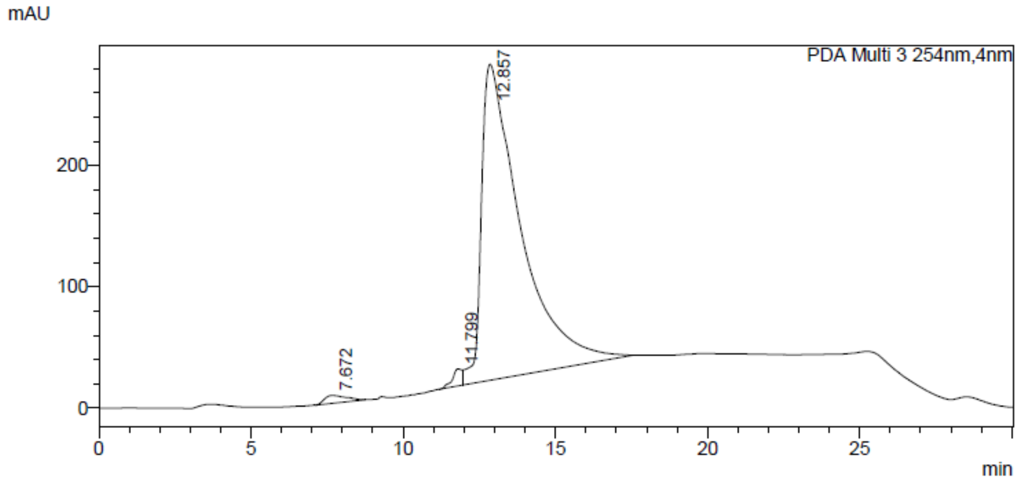

<Peak Table>

PDA Ch3 254nm

Peak#	Ret. Time	Area	Height	Area%
1	7.672	320528	6554	1.326
2	11.799	301440	14131	1.247
3	12.857	23542389	260774	97.426
Total		24164357	281458	100.000

4-Chloro-3-[(4-{2-[(5-{[2-(2,6-dioxopiperidin-3-yl)-1,3-dioxoisoindolin-4-yl]amino}pentyl)amino]-2-oxoethoxy}benzyl)amino]-N-hydroxybenzamide (CRBN_1g)

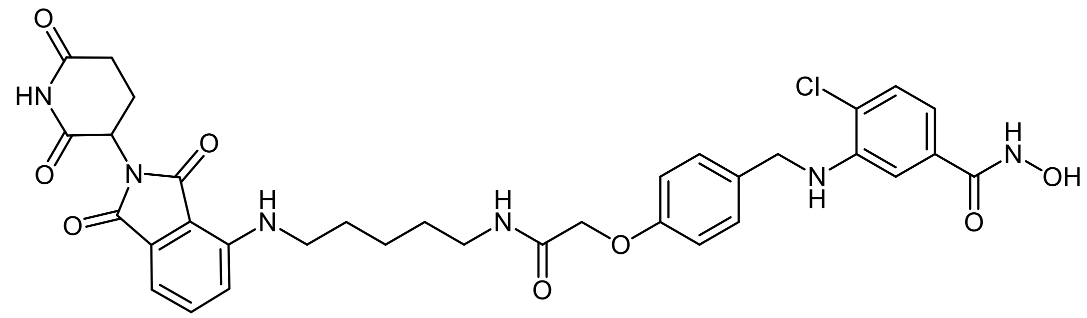

MS m/z: 689.24 $[M-H]^-$

^{1}H NMR (400 MHz, DMSO-d6) δ 11.06 (s, 2H), 8.93 (s, 1H), 8.01 (t, J = 5.7 Hz, 1H), 7.59 – 7.51 (m, 1H), 7.31 – 7.19 (m, 3H), 7.06 (d, J = 8.6 Hz, 1H), 7.00 (d, J = 7.0 Hz, 1H), 6.96 – 6.83 (m, 4H), 6.49 (t, J = 5.7 Hz, 1H), 6.14 (t, J = 6.1 Hz, 1H), 5.03 (dd, J = 12.9, 5.3 Hz, 1H), 4.40 (s, 2H), 4.35 (d, J = 6.0 Hz, 2H), 3.25 (dd, J = 13.5, 6.8 Hz, 2H), 3.11 (dd, J = 13.0, 6.6 Hz, 2H), 2.92 – 2.79 (m, 1H), 2.60 – 2.52 (m, 2H), 2.04 – 1.96 (m, 1H), 1.58 – 1.40 (m, 4H), 1.34 – 1.24 (m, 2H).

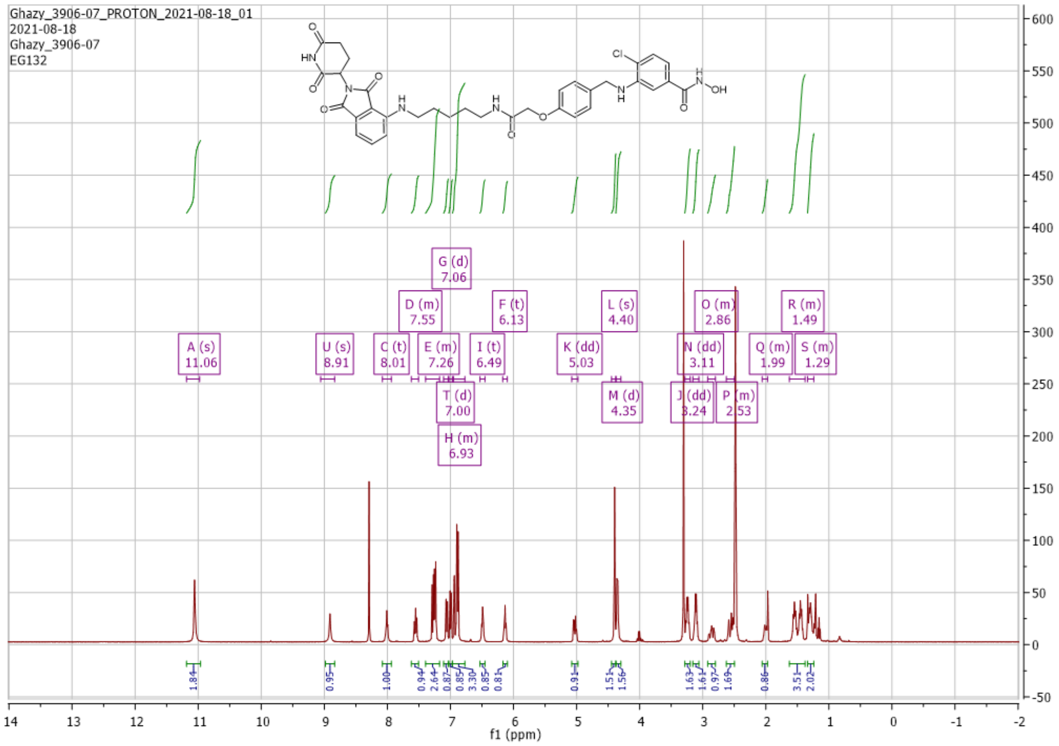

^{13}C NMR (101 MHz, DMSO-d6) δ 173.21, 170.51, 169.38, 167.91, 167.73, 164.34, 157.10, 146.85, 144.21, 136.71, 132.81, 132.63, 132.37, 129.25, 128.42, 120.85, 117.61, 115.21, 115.11, 110.83, 110.29, 109.48, 79.61, 67.53, 49.00, 45.70, 42.24, 38.56, 31.43, 30.83, 29.21, 28.78, 24.06, 22.61.

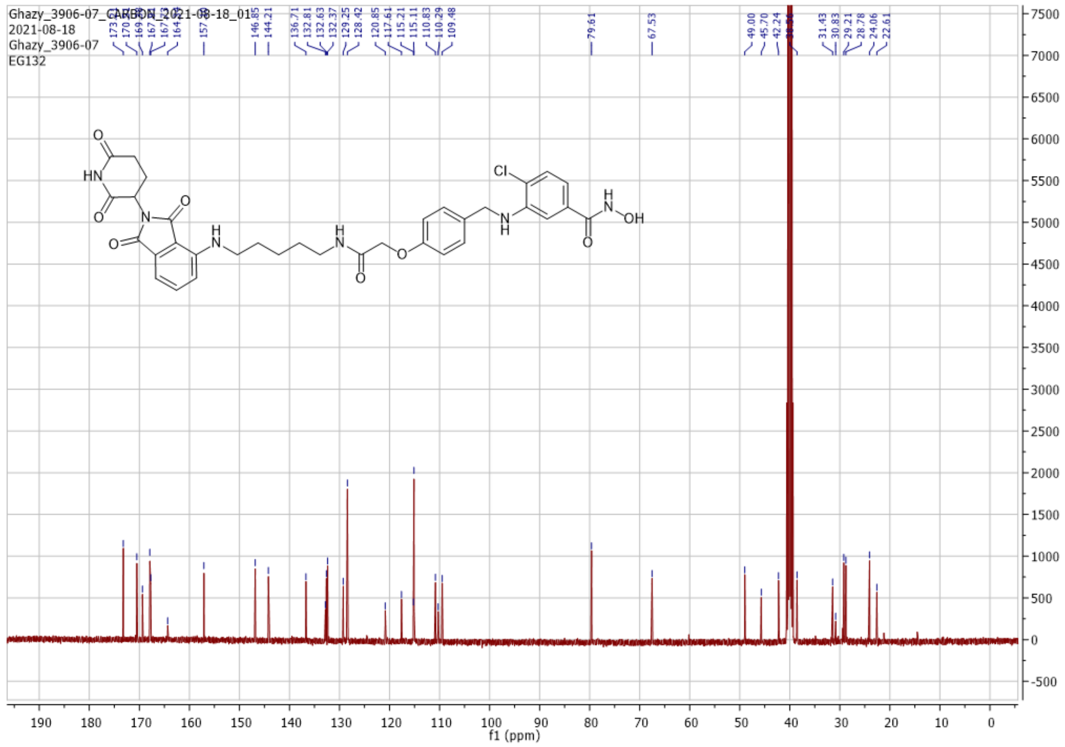

HRMS m/z: 691.229 [M+H]$^+$, calculated $C_{34}H_{36}ClN_6O_8^+$: 691.228

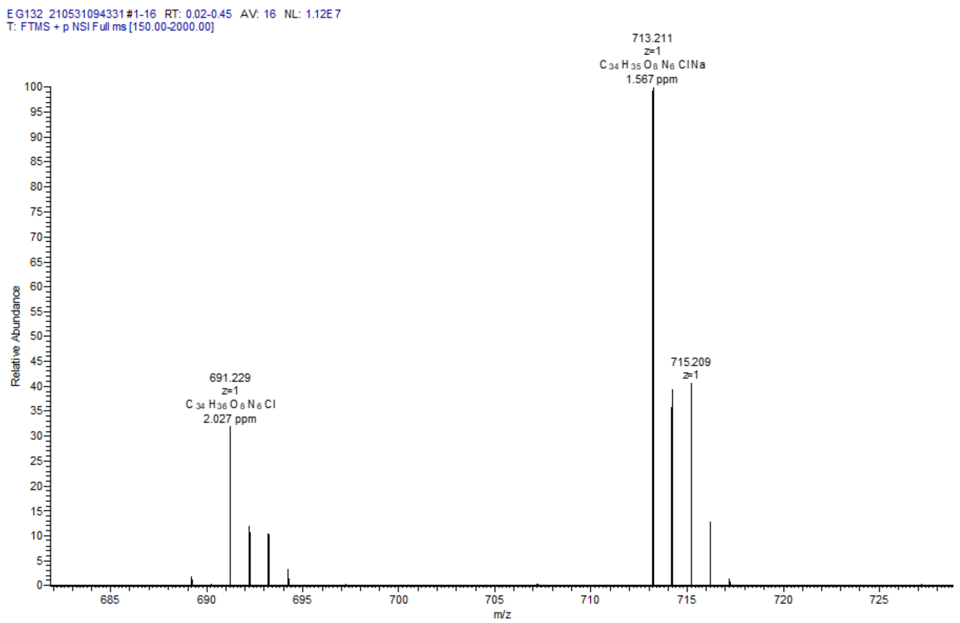

HPLC: (M2) rt 9.5 min (purity 98 %)

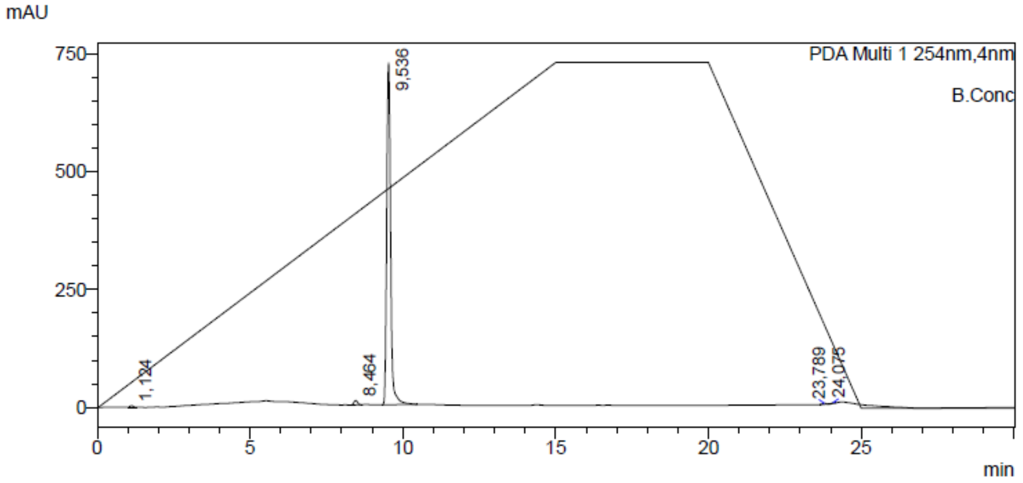

PDA Ch1 254nm

Peak#	Ret. Time	Area	Height	Area%
1	1,124	27377	3528	0,413
2	8,464	74474	9129	1,125
3	9,536	6491020	725418	98,031
4	23,789	18534	2364	0,280
5	24,075	9999	1735	0,151
Total		6621405	742174	100,000

4-Chloro-3-({4-[(16-{[2-(2,6-dioxopiperidin-3-yl)-1,3-dioxoisoindolin-4-yl]amino}-2-oxo-7,10,13-trioxa-3-azahexadecyl)oxy]benzyl}amino)-N-hydroxybenzamide (CRBN_1h)

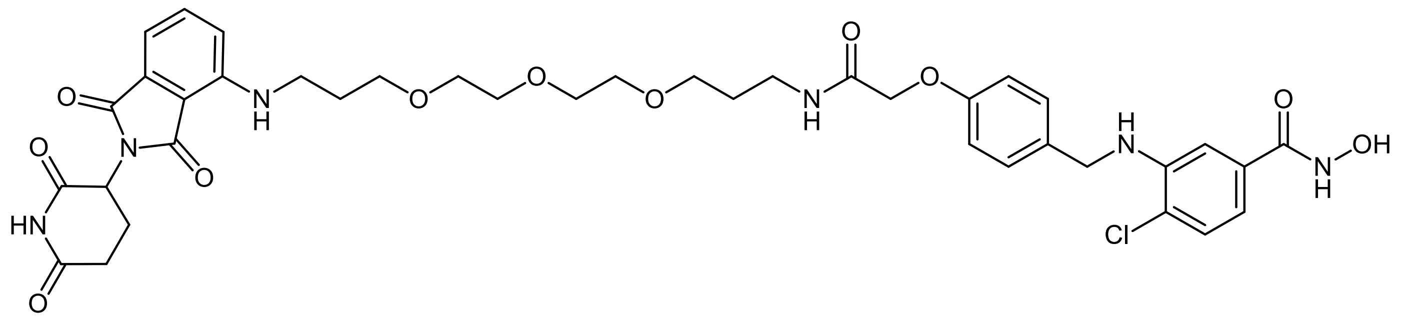

MS m/z: 807.53 [M-H]$^-$

^{1}H NMR (400 MHz, DMSO-d6) δ 11.05 (br s, 2H), 8.92 (s, 1H), 7.97 (t, J = 5.7 Hz, 1H), 7.55 (dd, J = 8.4, 7.2 Hz, 1H), 7.30 – 7.20 (m, 3H), 7.07 (d, J = 8.6 Hz, 1H), 6.99 (d, J = 6.9 Hz, 1H), 6.94 (d, J = 1.9 Hz, 1H), 6.91 – 6.85 (m, 3H), 6.63 (t, J = 5.9 Hz, 1H), 6.14 (t, J = 6.1 Hz, 1H), 5.02 (dd, J = 12.8, 5.4 Hz, 1H), 4.39 (s, 2H), 4.35 (d, J = 6.1 Hz, 2H), 3.55 – 3.39 (m, 10H), 3.40 – 3.29 (m, 4H), 3.14 (dd, J = 12.9, 6.7 Hz, 2H), 2.92 – 2.79 (m, 1H), 2.66 – 2.52 (m, 2H), 2.04 – 1.95 (m, 1H), 1.82 – 1.73 (m, 2H), 1.67 – 1.56 (m, 2H).

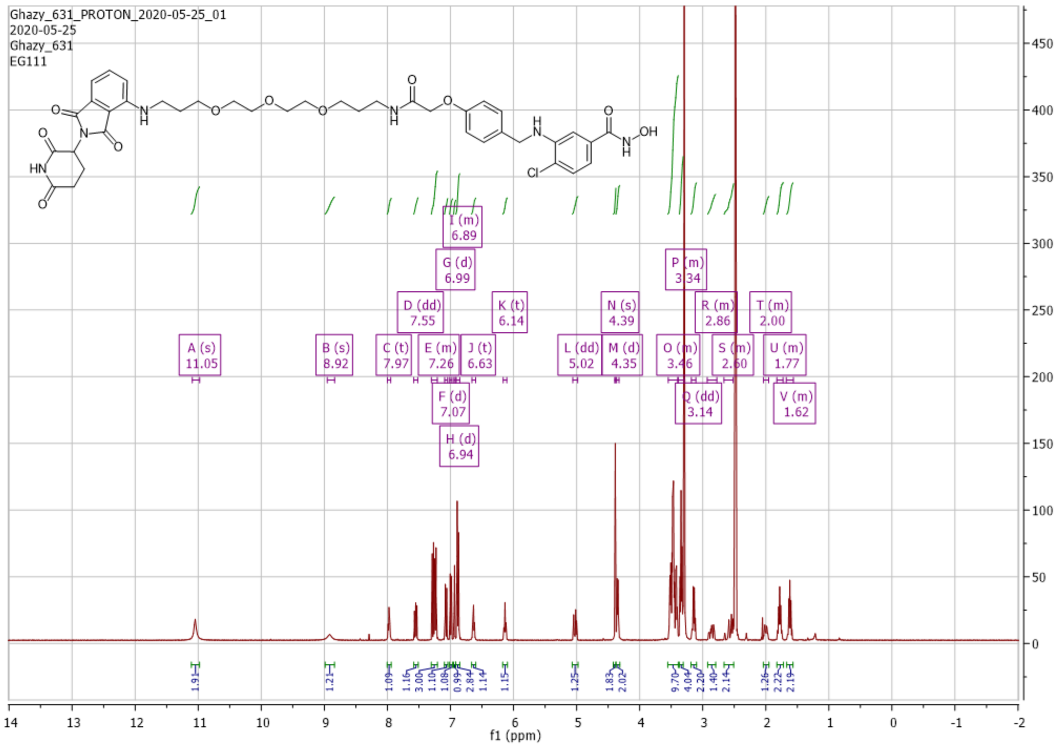

^{13}C NMR (101 MHz, DMSO-d6) δ 173.22, 170.50, 169.27, 167.94, 167.75, 157.06, 146.88, 144.21, 136.70, 132.82, 132.63, 132.40, 129.25, 128.42, 117.52, 115.20, 115.10, 110.81, 110.28, 109.51, 70.22, 70.16, 70.12, 69.98, 68.65, 67.52, 48.98, 45.69, 36.30, 31.42, 29.69, 29.32, 22.61.

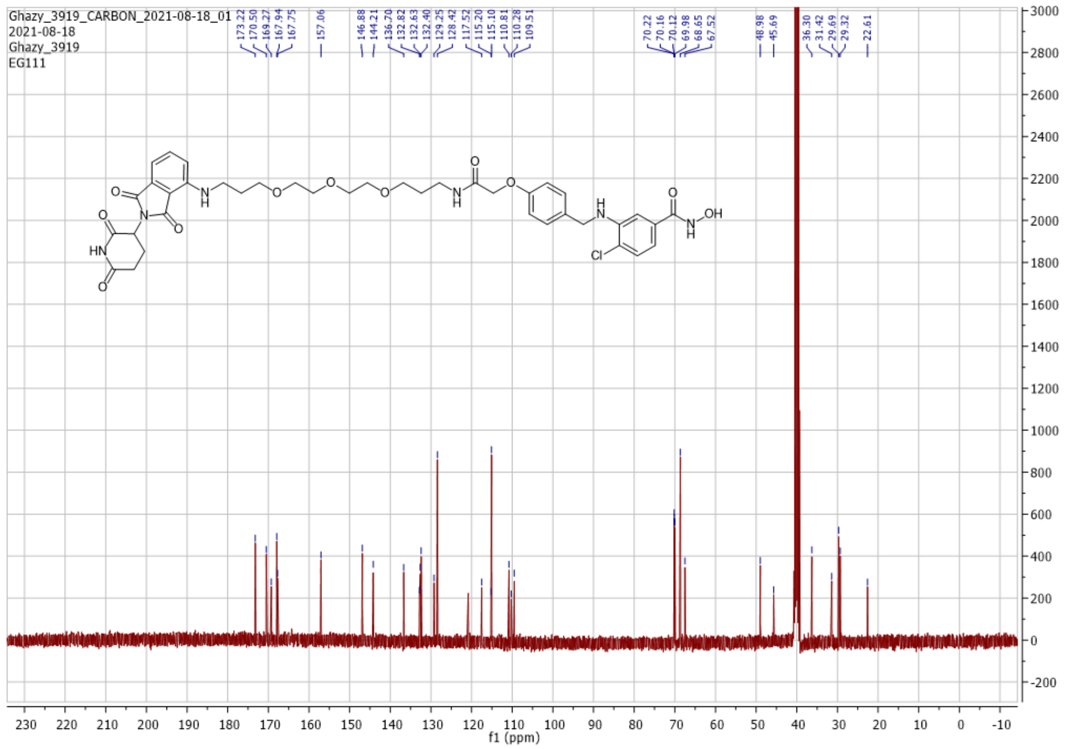

HRMS m/z: 809.292 [M+H]+, calculated $C_{39}H_{46}ClN_6O_{11}^+$ 809.291

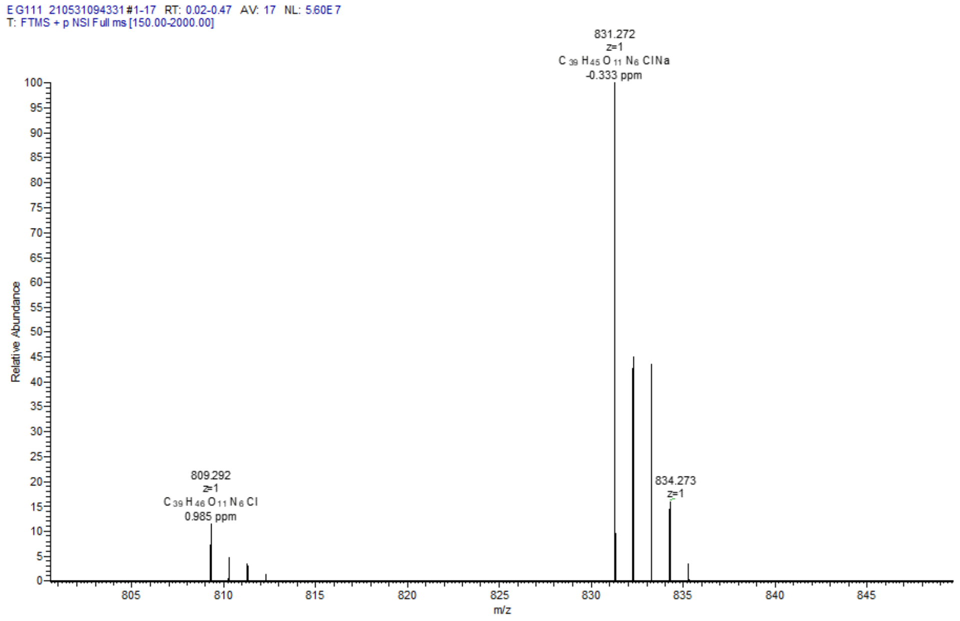

HPLC: (M2) rt 9.8 min (purity >99 %)

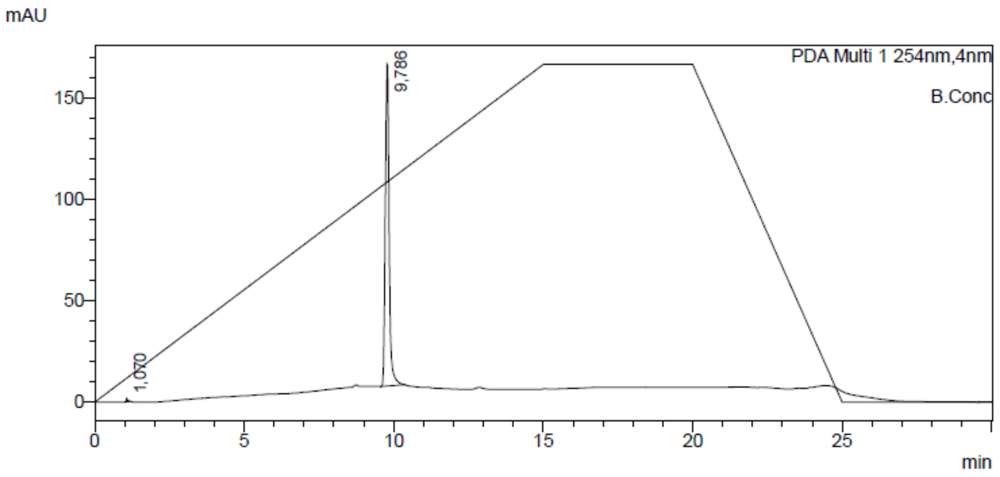

PDA Ch1 254nm

Peak#	Ret. Time	Area	Height	Area%
1	1,070	5115	1731	0,362
2	9,786	1408076	158499	99,638
Total		1413191	160230	100,000

(2S,4R)-1-[(S)-2-(6-{2-[4-({[2-chloro-5-(hydroxycarbamoyl)phenyl]amino}methyl) phenoxy]acetamido}hexanamido)-3,3-dimethylbutanoyl]-4-hydroxy-N-[4-(4-methylthiazol-5-yl)benzyl]pyrrolidine-2-carboxamide (VHL_1l)

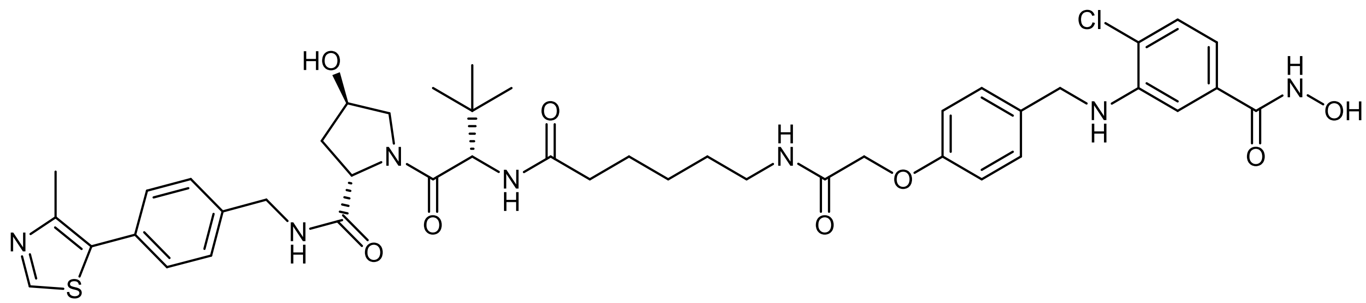

MS m/z: 874.38 [M-H]$^{-}$

^{1}H NMR (400 MHz, DMSO-d6) δ 11.06 (br s, 1H), 9.00 (s, 1H), 8.54 (t, J = 6.0 Hz, 1H), 7.99 (t, J = 5.8 Hz, 1H), 7.81 (d, J = 9.4 Hz, 1H), 7.43 – 7.33 (m, 4H), 7.30 – 7.20 (m, 3H), 6.96 – 6.85 (m, 4H), 5.16 (br s, 2H), 4.52 (d, J = 9.3 Hz, 1H), 4.43 – 4.29 (m, 6H), 3.69 – 3.52 (m, 3H), 3.07 (dd, J = 13.2, 6.7 Hz, 2H), 2.43 (s, 3H), 2.26 – 2.16 (m, 1H), 2.11 – 1.98 (m, 2H), 1.93 – 1.83 (m, 1H), 1.77 – 1.71 (m, 1H), 1.52 – 1.32 (m, 4H), 1.25 – 1.15 (m, 3H), 0.91 (s, 9H).

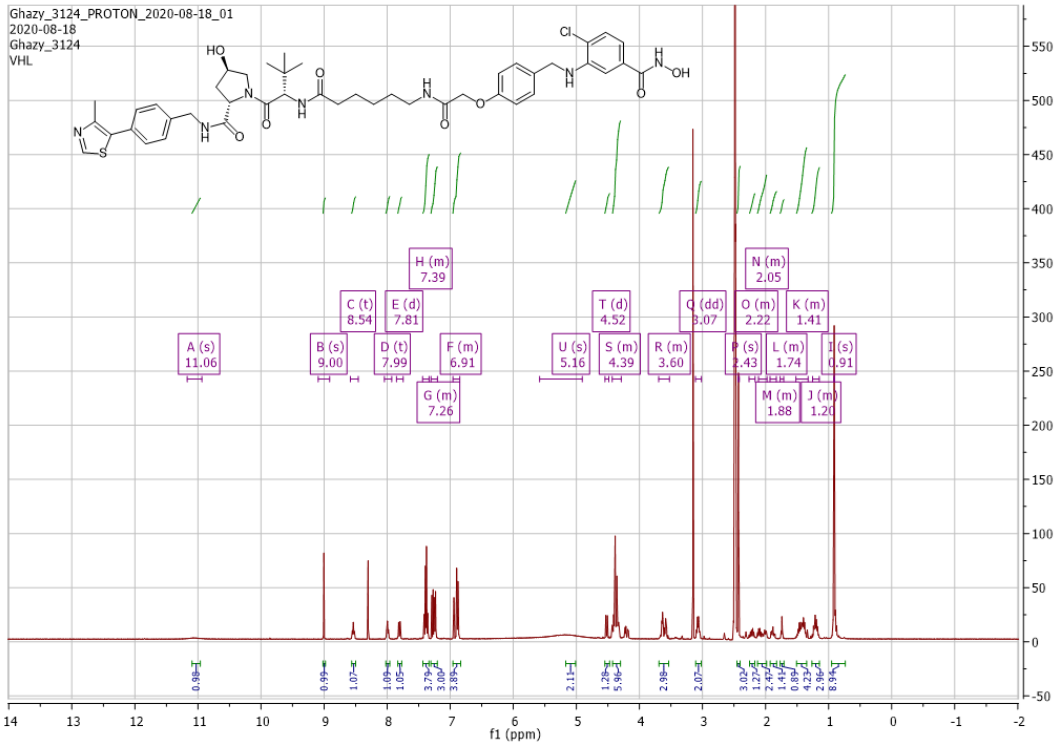

^{13}C NMR (101 MHz, DMSO-d6) δ 172.48, 172.37, 170.14, 167.83, 164.28, 157.10, 152.05, 147.87, 144.20, 140.02, 132.36, 129.94, 129.25, 129.08, 128.41, 127.87, 120.84, 115.23, 115.10, 110.30, 79.63, 69.30, 67.51, 59.13, 56.74, 38.63, 38.38, 35.64, 35.29, 29.29, 26.83, 26.50, 25.61, 16.26.

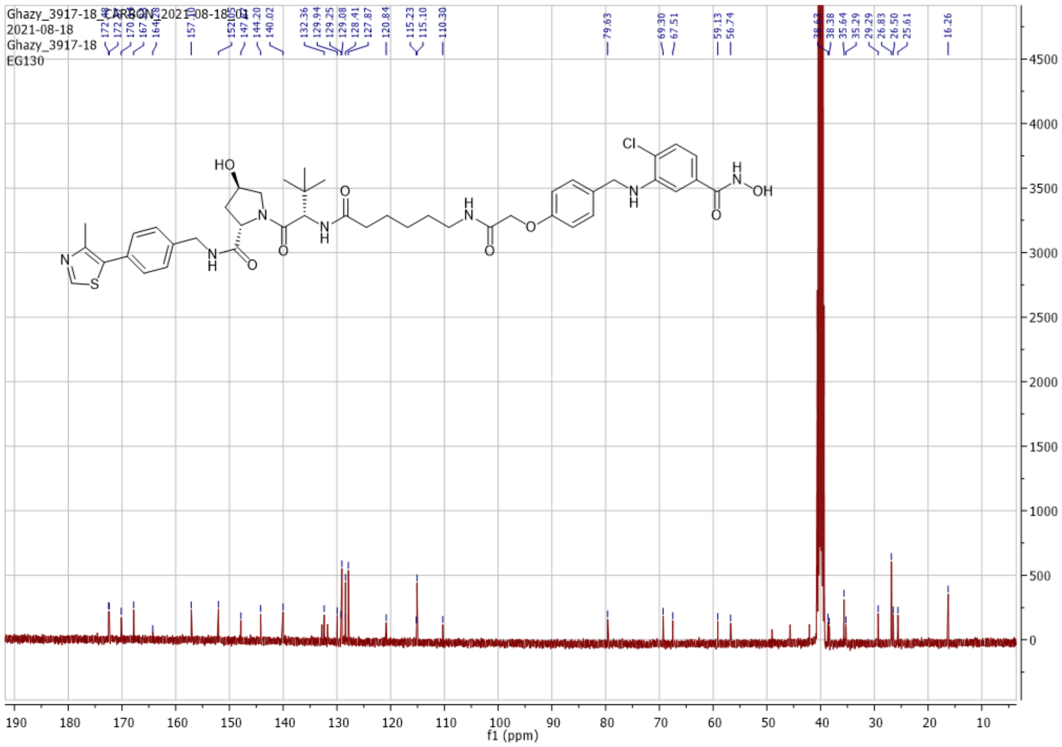

HRMS m/z: 876.353 $[M+H]^+$, calculated $C_{44}H_{55}ClN_7O_8S^+$: 876.352

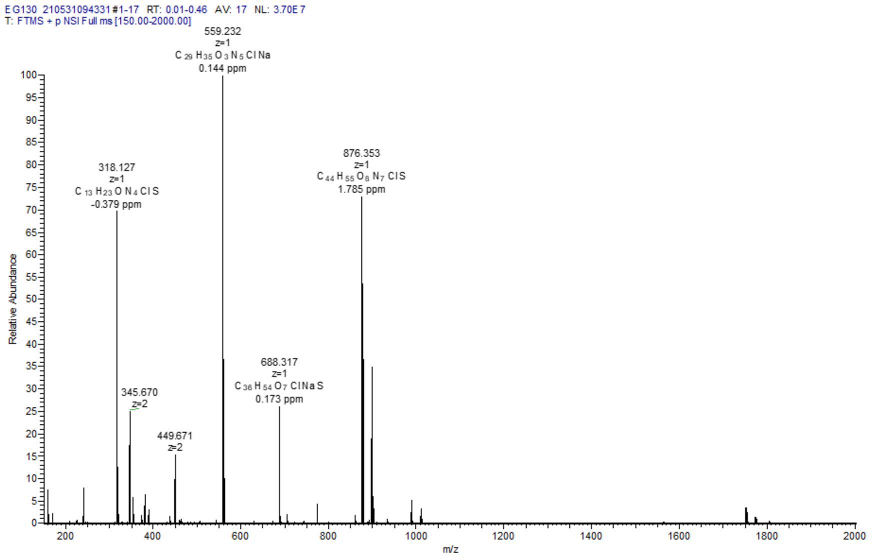

HPLC: (M2) rt. 10.2 min (purity 96%)

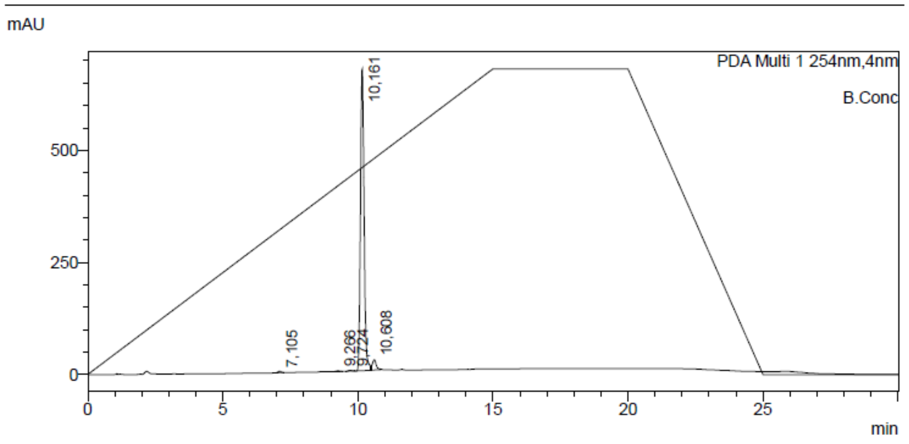

PDA Ch1 254nm

Peak#	Ret. Time	Area	Height	Area%
1	7,105	26877	3247	0,412
2	9,266	14903	1794	0,228
3	9,724	15567	1848	0,239
4	10,161	6258762	673035	95,921
5	10,608	208778	22776	3,200
Total		6524887	702700	100,000

3-({4-[2-({5-[2-(Adamantan-1-yl)acetamido]pentyl}amino)-2-oxoethoxy]benzyl}amino)-4-chloro-N-hydroxybenzamide (HyT_1p)

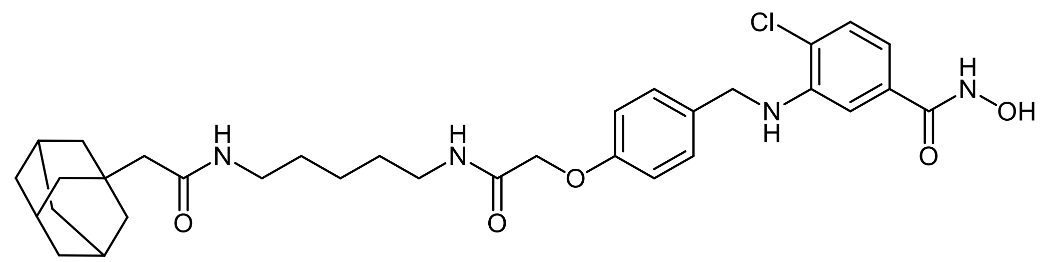

MS m/z: 609.65 [M-H]$^{-}$

^{1}H NMR (400 MHz, DMSO-d6) δ 11.05 (s, 1H), 8.91 (s, 1H), 7.98 (t, J = 5.8 Hz, 1H), 7.59 (s, 1H), 7.30 – 7.21 (m, 3H), 6.96 – 6.84 (m, 4H), 6.15 (t, J = 6.0 Hz, 1H), 4.39 (s, 2H), 4.36 (d, J = 6.1 Hz, 2H), 3.15 (d, J = 5.3 Hz, 1H), 3.08 (dd, J = 13.1, 6.6 Hz, 2H), 2.96 (dd, J = 12.8, 6.7 Hz, 2H), 1.88 (s, 3H), 1.78 (s, 2H), 1.66 – 1.49 (m, 11H), 1.42 – 1.30 (m, 4H), 1.25 – 1.15 (m, 2H).

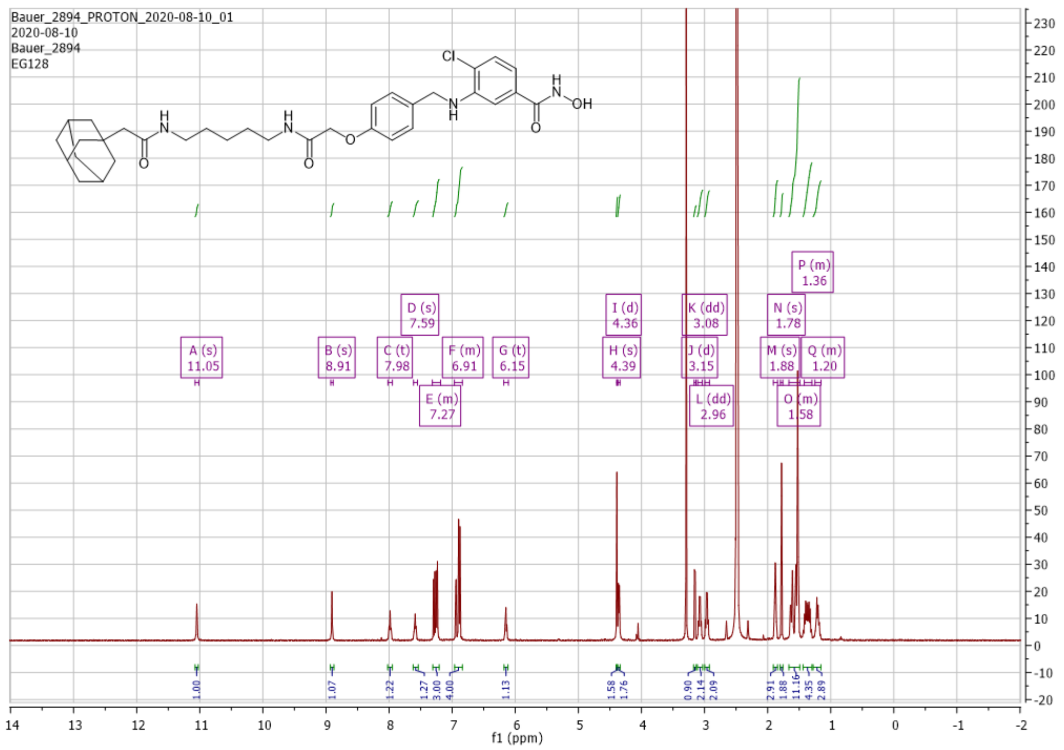

^{13}C NMR (101 MHz, DMSO-d6) δ 170.13, 167.85, 164.33, 157.10, 144.21, 132.81, 132.37, 129.25, 128.40, 115.21, 115.10, 110.29, 67.58, 50.51, 45.69, 42.59, 39.36, 38.65, 36.92, 32.58, 29.30, 29.19, 28.49, 24.22.

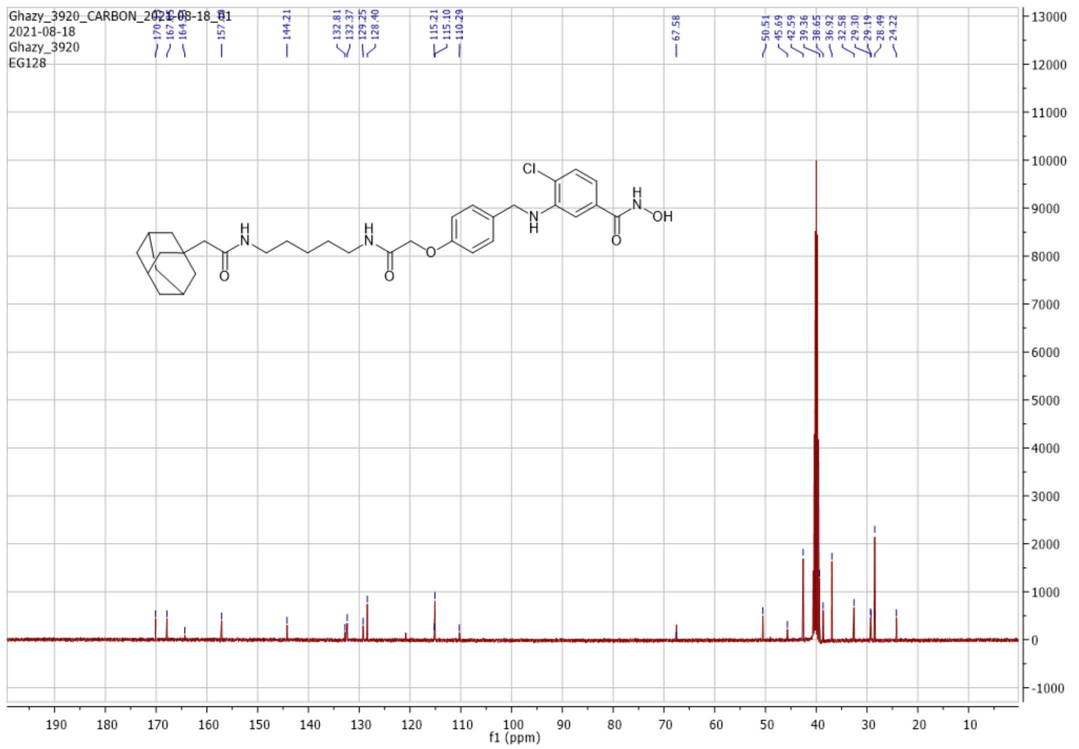

HRMS m/z: 611.300 $[M+H]^+$, calculated $C_{33}H_{44}ClN_4O_5^+$: 611.300

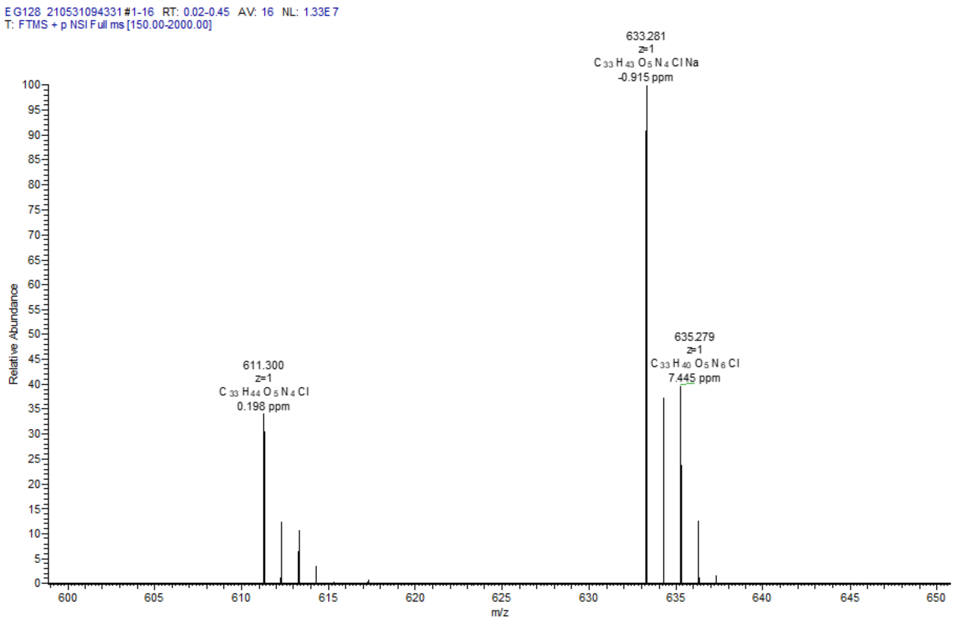

HPLC: (M2) rt. 11 min (purity 98 %)

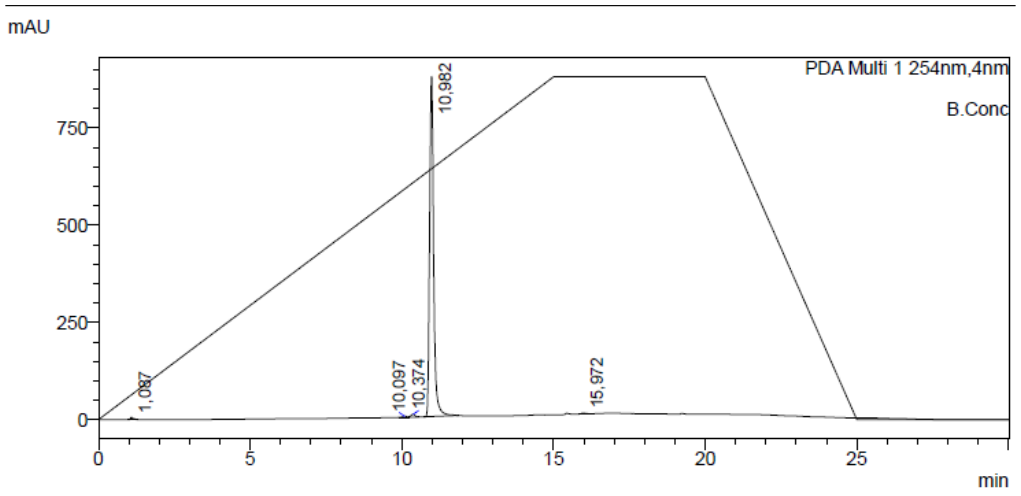

PDA Ch1 254nm

Peak#	Ret. Time	Area	Height	Area%
1	1,087	36818	6186	0,447
2	10,097	23990	2639	0,291
3	10,374	63967	7443	0,777
4	10,982	8094761	873529	98,283
5	15,972	16627	2236	0,202
Total		8236163	892034	100,000

4-Chloro-3-(4-[(16-{[2-(2,6-dioxopiperidin-3-yl)-1,3-dioxoisoindolin-4-yl]amino}-2-oxo-7,10,13-trioxa-3-azahexadecyl)oxy]benzamido}-N-hydroxybenzamide (CRBN_1i)

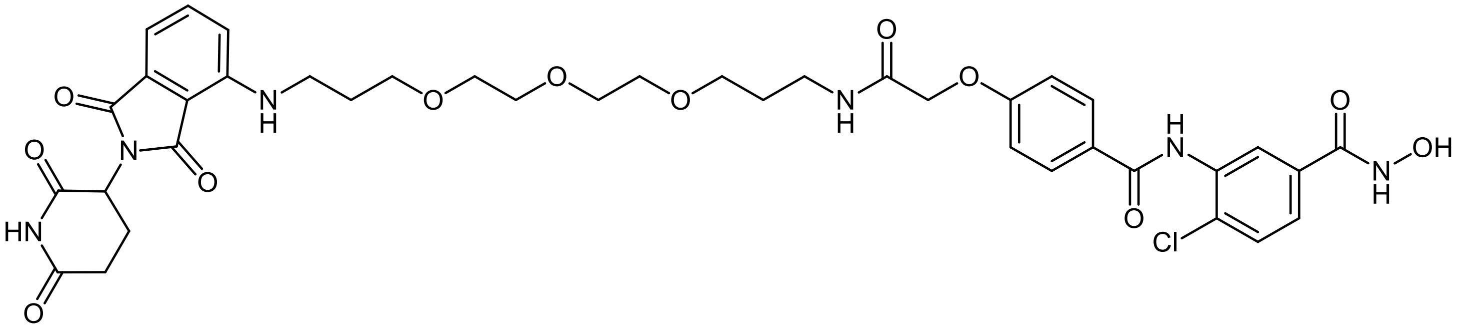

MS m/z: 821.47 $[M-H]^-$

^{1}H NMR (400 MHz, DMSO-d6) δ 11.28 (s, 1H), 11.05 (s, 1H), 9.99 (s, 1H), 9.11 (s, 1H), 8.09 (t, J = 5.6 Hz, 1H), 7.99 – 7.93 (m, 3H), 7.66 – 7.49 (m, 3H), 7.13 – 6.96 (m, 4H), 6.64 (t, J = 5.8 Hz, 1H), 5.02 (dd, J = 12.8, 5.4 Hz, 1H), 4.55 (s, 2H), 3.57 – 3.29 (m, 14H), 3.17 (dd, J = 12.8, 6.7 Hz, 2H), 2.92 – 2.79 (m, 1H), 2.61 – 2.49 (m, 2H), 2.05 – 1.95 (m, 1H), 1.83 – 1.73 (m, 2H), 1.69 – 1.59 (m, 2H).

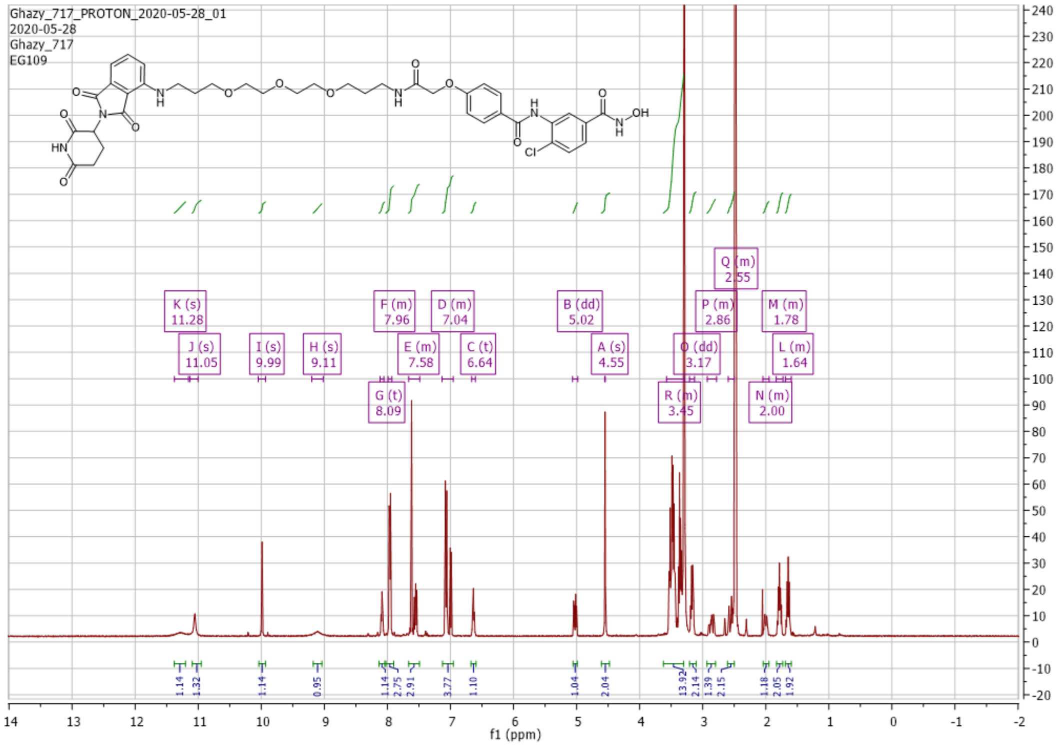

^{13}C NMR (101 MHz, DMSO-d6) δ 173.22, 170.50, 169.27, 167.75, 167.49, 165.20, 161.03, 146.88, 136.70, 135.81, 132.63, 132.39, 130.04, 127.37, 126.90, 125.79, 117.52, 114.95, 110.81, 109.51, 70.23, 70.18, 70.13, 70.00, 68.65, 67.46, 48.98, 36.33, 31.42, 29.71, 29.33, 22.61.

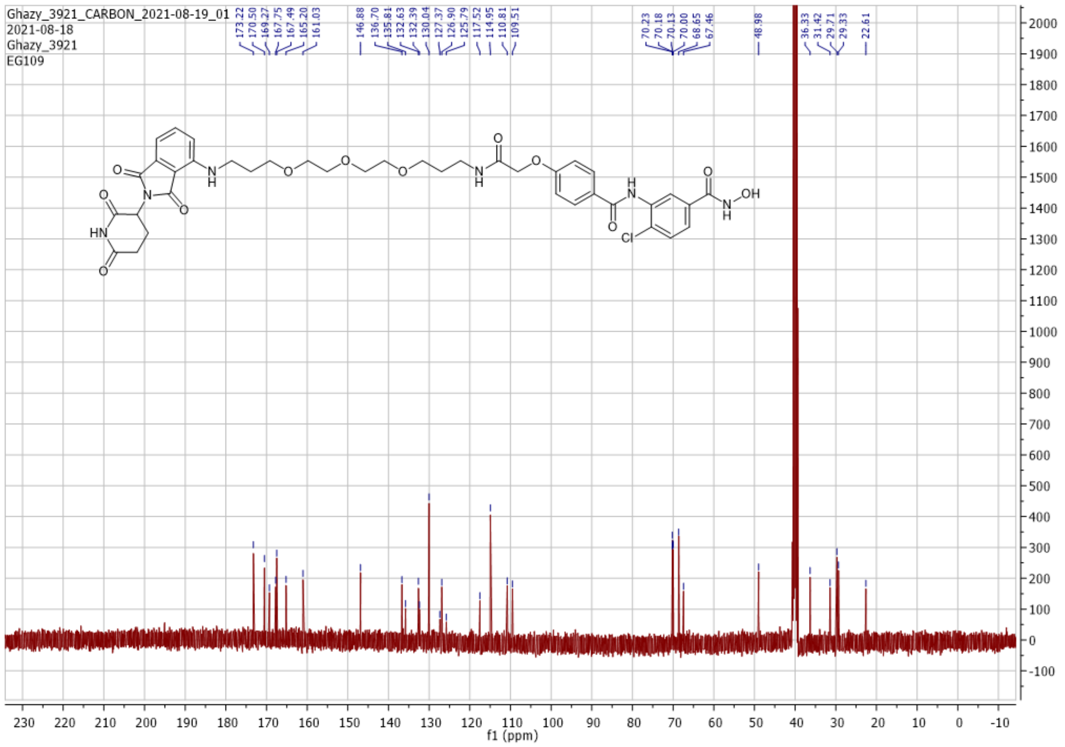

HRMS m/z: 823.271 [M+H]$^+$, calculated $C_{39}H_{44}ClN_6O_{12}^+$: 823.271

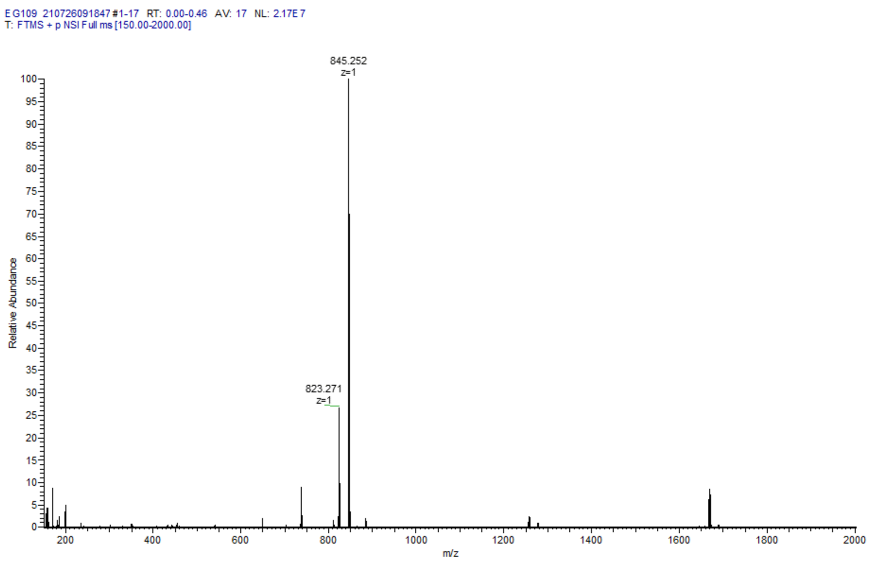

HPLC: (M2) rt. 9.1 min (purity 99%)

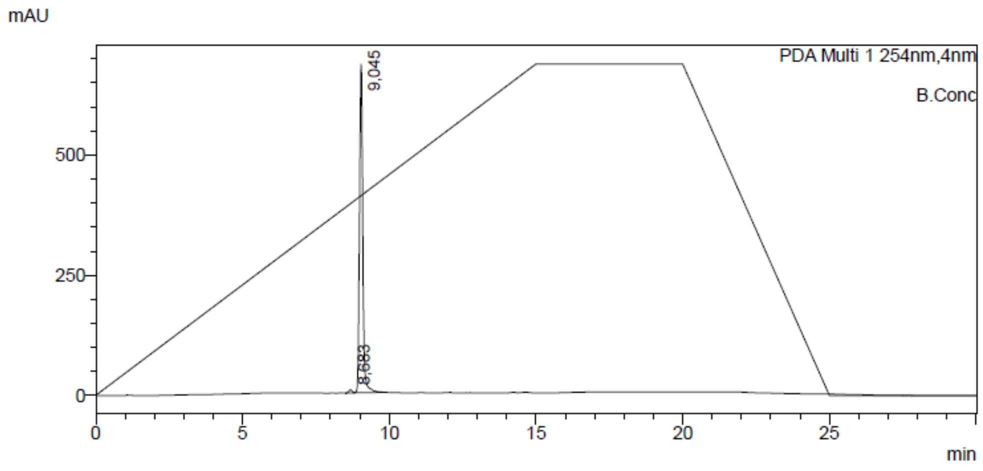

PDA Ch1 254nm

Peak#	Ret. Time	Area	Height	Area%
1	8,683	52531	6708	0,975
2	9,045	5332790	683992	99,025
Total		5385321	690700	100,000

3-{4-[(16-{[2-(2,6-Dioxopiperidin-3-yl)-1,3-dioxoisoindolin-4-yl]amino}-2-oxo-7,10,13-trioxa-3-azahexadecyl)oxy]benzamido}-N-hydroxybenzamide (CRBN_1j)

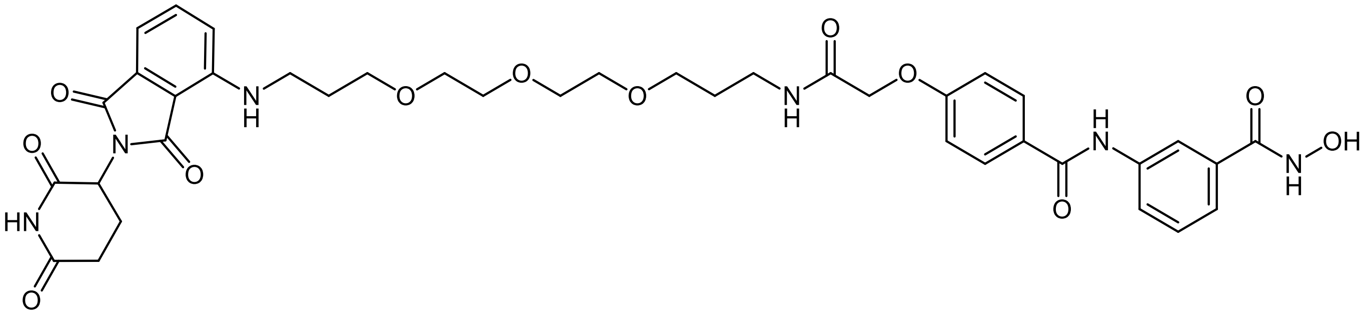

MS m/z: 787.37 $[M-H]^-$

^{1}H NMR (400 MHz, DMSO-d6) δ 11.16 (s, 1H), 11.06 (s, 1H), 10.21 (s, 1H), 8.99 (s, 1H), 8.16 (s, 1H), 8.09 (t, J = 5.5 Hz, 1H), 7.99 – 7.89 (m, 3H), 7.59 – 7.51 (m, 1H), 7.44 – 7.33 (m, 2H), 7.10 – 7.04 (m, 3H), 7.00 (d, J = 7.1 Hz, 1H), 6.64 (t, J = 5.7 Hz, 1H), 5.02 (dd, J = 12.8, 5.5 Hz, 1H), 4.55 (s, 2H), 3.56 – 3.29 (m, 14H), 3.19 – 3.13 (m, 2H), 2.86 – 2.78 (m, 1H), 2.67 – 2.50 (m, 2H), 2.05 – 1.93 (m, 1H), 1.83 – 1.74 (m, 2H), 1.68 – 1.59 (m, 2H).

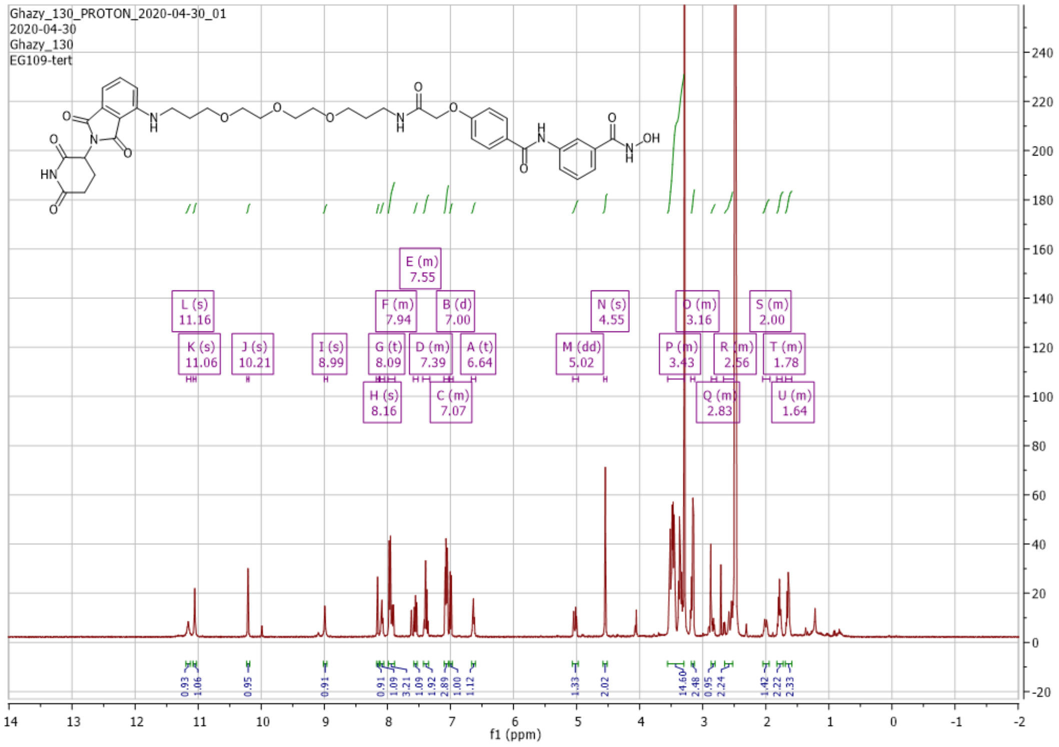

HRMS m/z: 789.310 $[M+H]^+$, calculated $C_{39}H_{45}N_6O_{12}^+$: 789.310

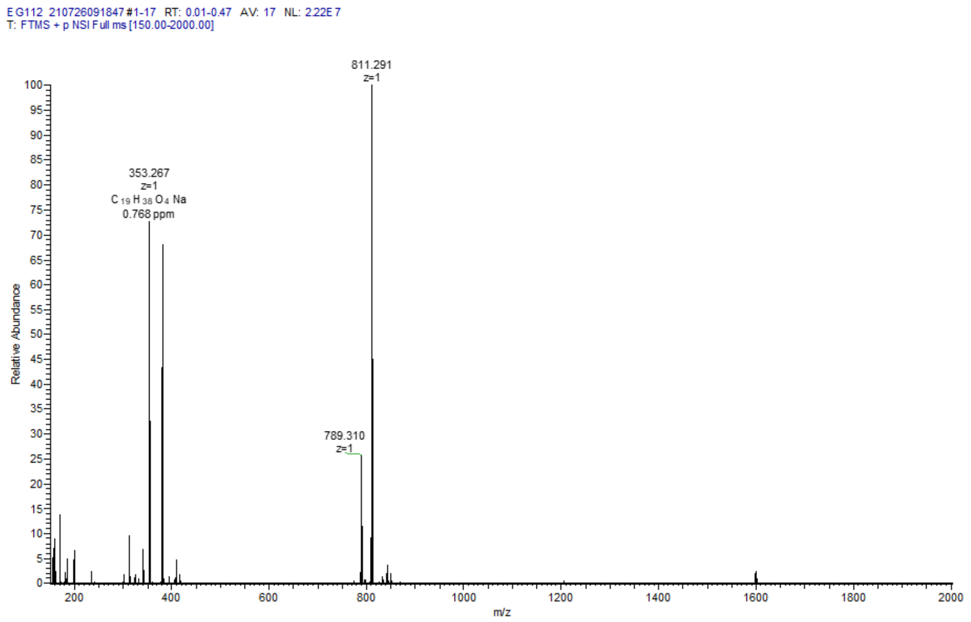

HPLC: (M2) rt 8.7 min (purity 100%)

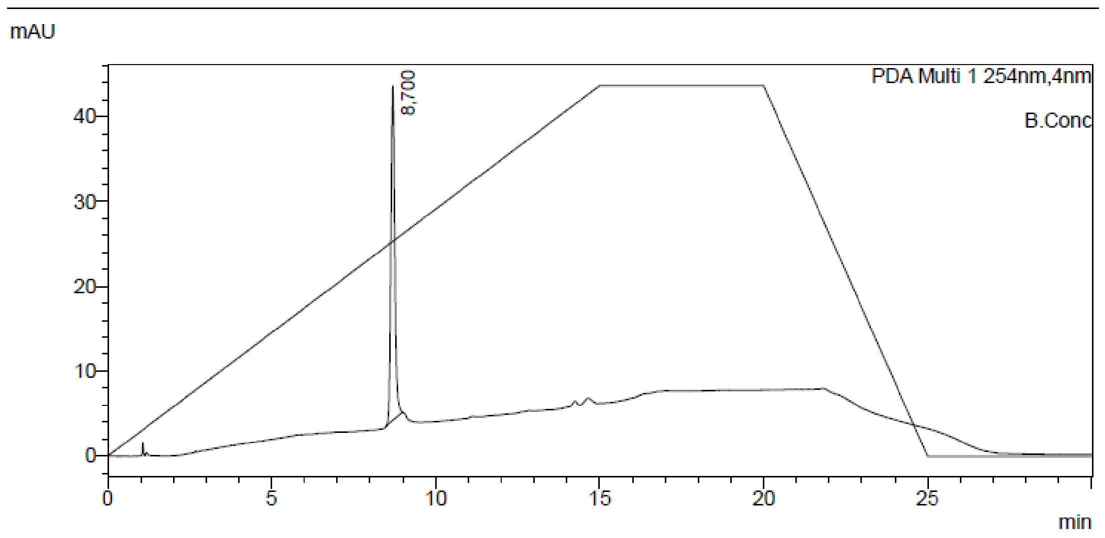

PDA Ch1 254nm

Peak#	Ret. Time	Area	Height	Area%
1	8,700	318889	39425	100,000
Total		318889	39425	100,000

Methyl 3-((4-((1-(6-((2-(2,6-dioxopiperidin-3-yl)-1,3-dioxoisoindolin-4-yl)amino)-6-oxohexyl)-1H-1,2,3-triazol-4-yl)methoxy)benzyl)amino)-4-methylbenzoate (33a)

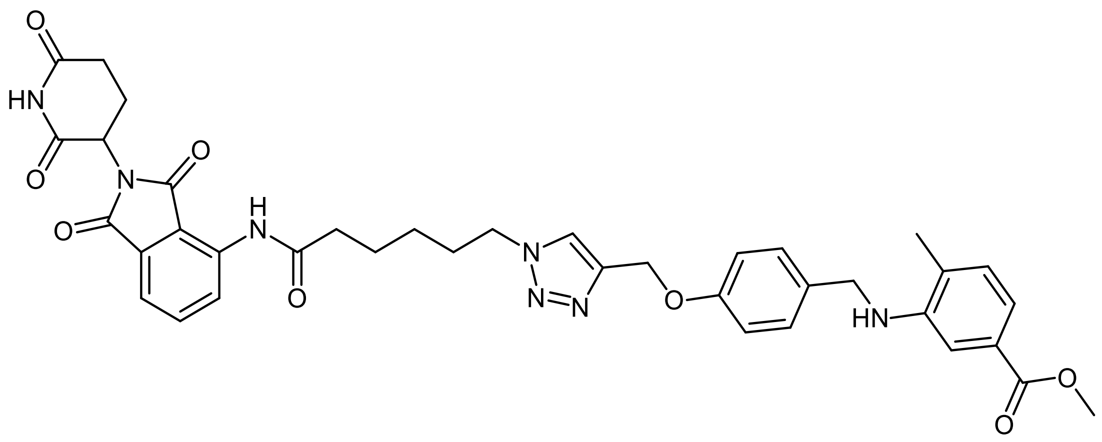

MS m/z: 721.38

^{1}H NMR (400 MHz, DMSO-d6) δ 11.12 (s, 1H), 9.67 (s, 1H), 8.44 (d, J = 8.2 Hz, 1H), 8.18 (s, 1H), 7.84 – 7.75 (m, 1H), 7.58 (dd, J = 7.2, 0.5 Hz, 1H), 7.26 (d, J = 8.7 Hz, 2H), 7.11 – 7.02 (m, 2H), 6.99 – 6.88 (m, 3H), 5.78 (t, J = 5.7 Hz, 1H), 5.12 (dd, J = 12.8, 5.4 Hz, 1H), 5.06 (s, 2H), 4.34 (t, J = 7.1 Hz, 2H), 4.30 (d, J = 5.3 Hz, 2H), 3.72 (s, J = 17.8 Hz, 3H), 2.63 – 2.49 (m, 2H), 2.44 (t, J = 7.4 Hz, 2H), 2.19 (s, 3H), 2.11 – 2.00 (m, 1H), 1.84 (dt, J = 14.7, 7.3 Hz, 2H), 1.63 (dt, J = 15.1, 7.6 Hz, 2H), 1.54 – 1.38 (m, 1H), 1.35 – 1.22 (m, 2H).

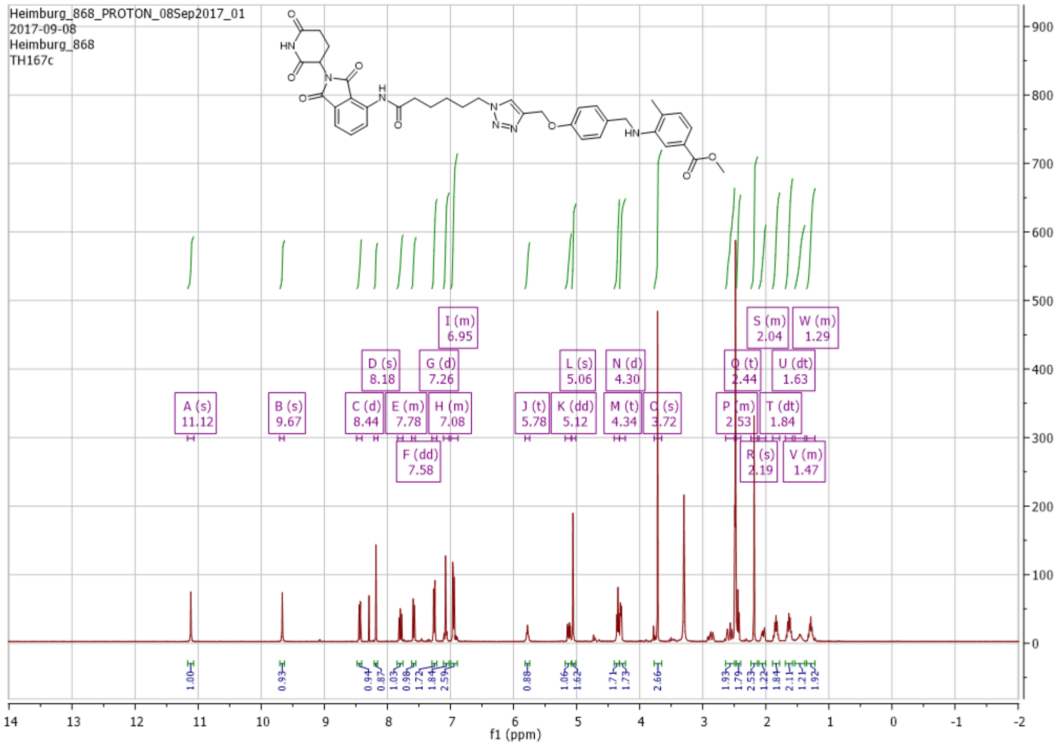

HRMS: 722.294 [M+H]+ , calculated $C_{38}H_{40}N_7O_8$ 722.294

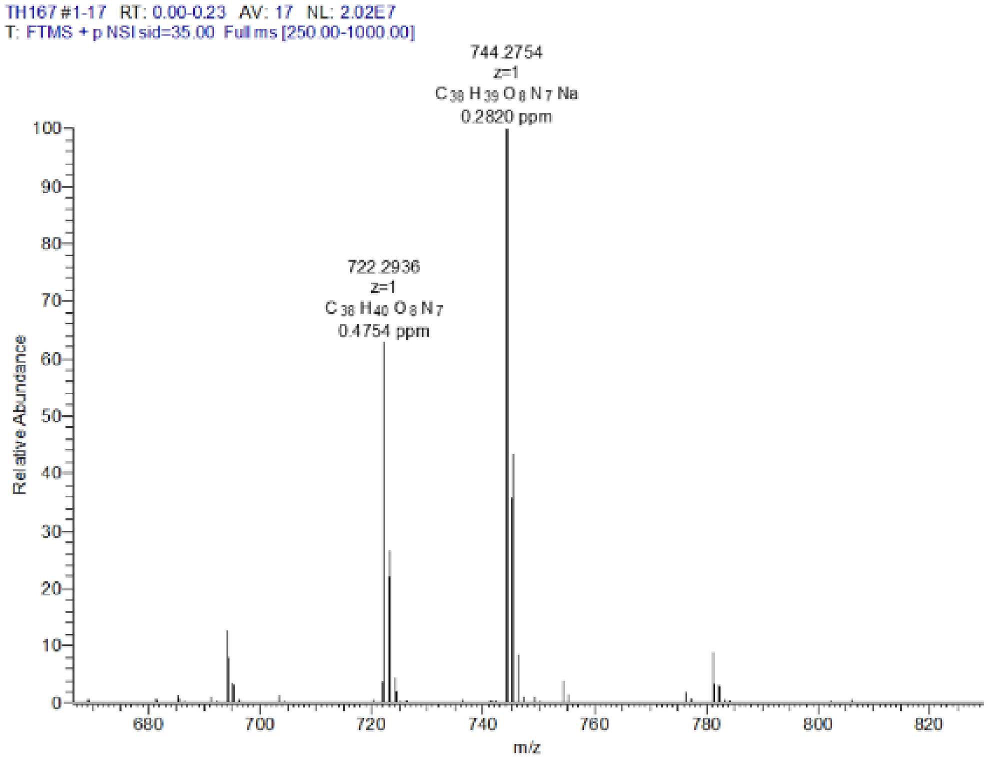

HPLC: (M1) rt: 10.9 min (purity 99 %)

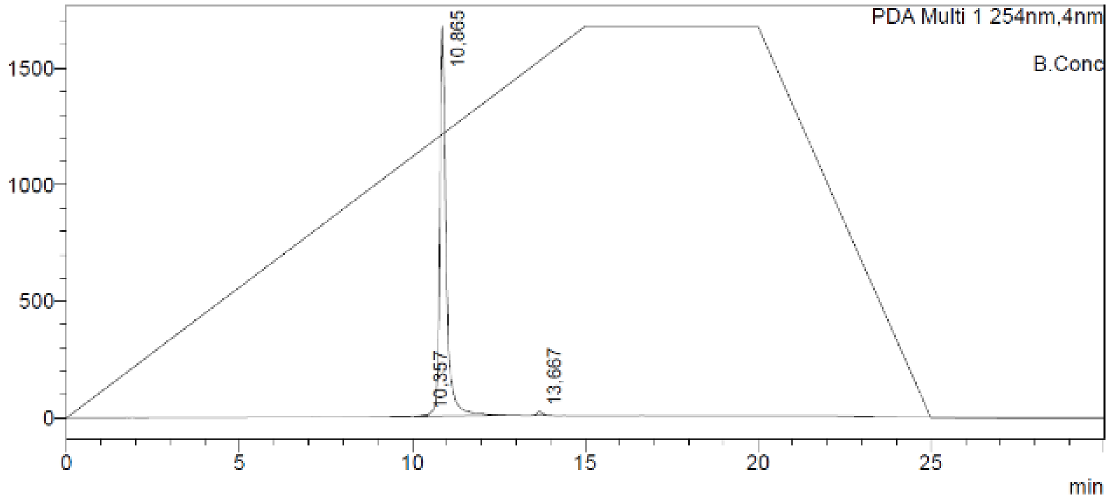

PDA Ch1 254nm

Peak#	Ret. Time	Area	Height	Area%
1	10,357	81788	7460	0,359
2	10,865	22465483	1673083	98,659
3	13,667	223631	19016	0,982
Total		22770902	1699560	100,000

3-((4-((1-benzyl-1H-1,2,3-triazol-4-yl)methoxy)benzyl)amino)-N-hydroxy-4-methylbenzamide (33b)

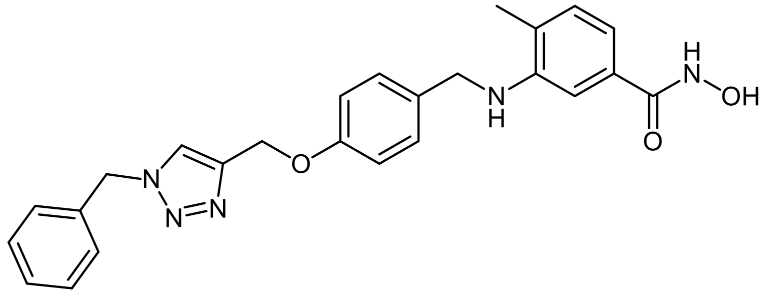

^{1}H NMR (400 MHz, DMSO-d6) δ 10.89 (s, 1H), 8.75 (s, 1H), 8.23 (s, 1H), 7.42 – 7.11 (m, 7H), 6.98 (d, J = 7.7 Hz, 1H), 6.94 (d, J = 8.7 Hz, 2H), 6.85 (d, J = 7.9 Hz, 1H), 6.81 (s, 1H), 5.59 (s, 1H), 5.58 (s, 2H), 5.07 (s, 2H), 4.30 (s, 2H), 2.14 (s, 3H).

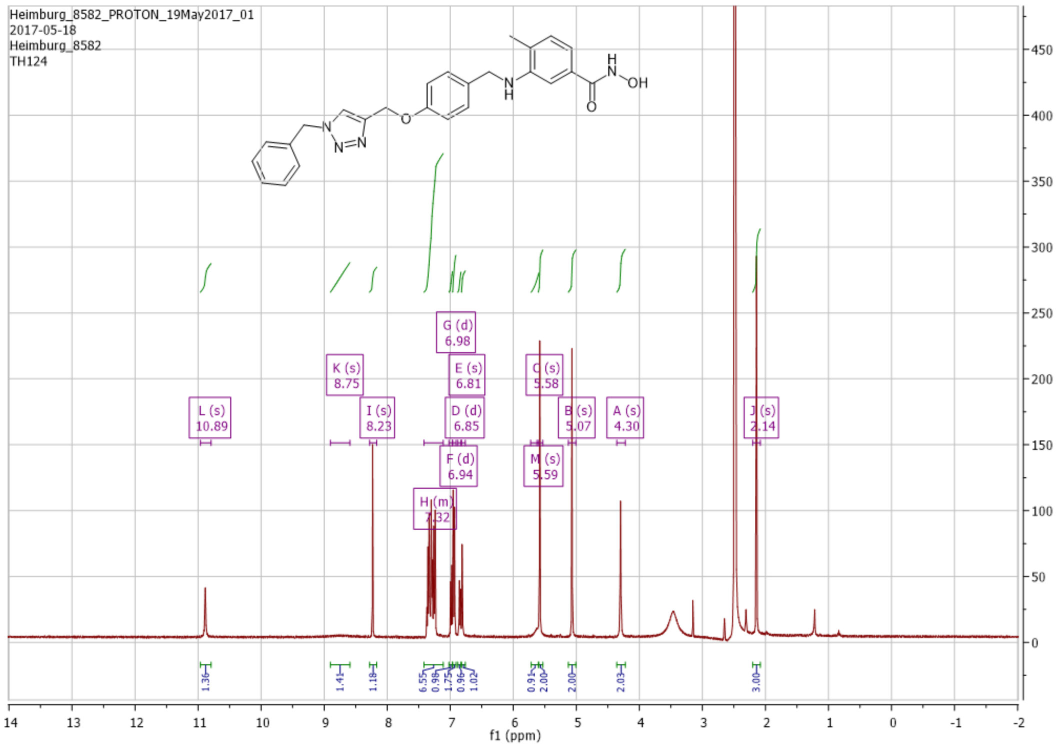

Pomalidomide (38)

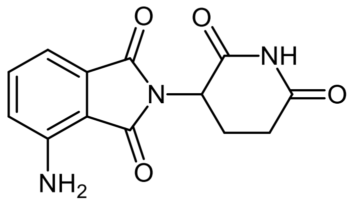

^{1}H NMR (400 MHz, DMSO-d6) δ 11.05 (s, 1H), 7.45 (dd, J = 8.4, 7.1 Hz, 1H), 6.99 (t, J = 7.3 Hz, 2H), 6.49 (s, 2H), 5.03 (dd, J = 12.9, 5.4 Hz, 1H), 2.87 (ddd, J = 17.4, 14.1, 5.4 Hz,1H), 2.66 – 2.50 (m, 2H), 2.06 – 1.90 (m, 1H).

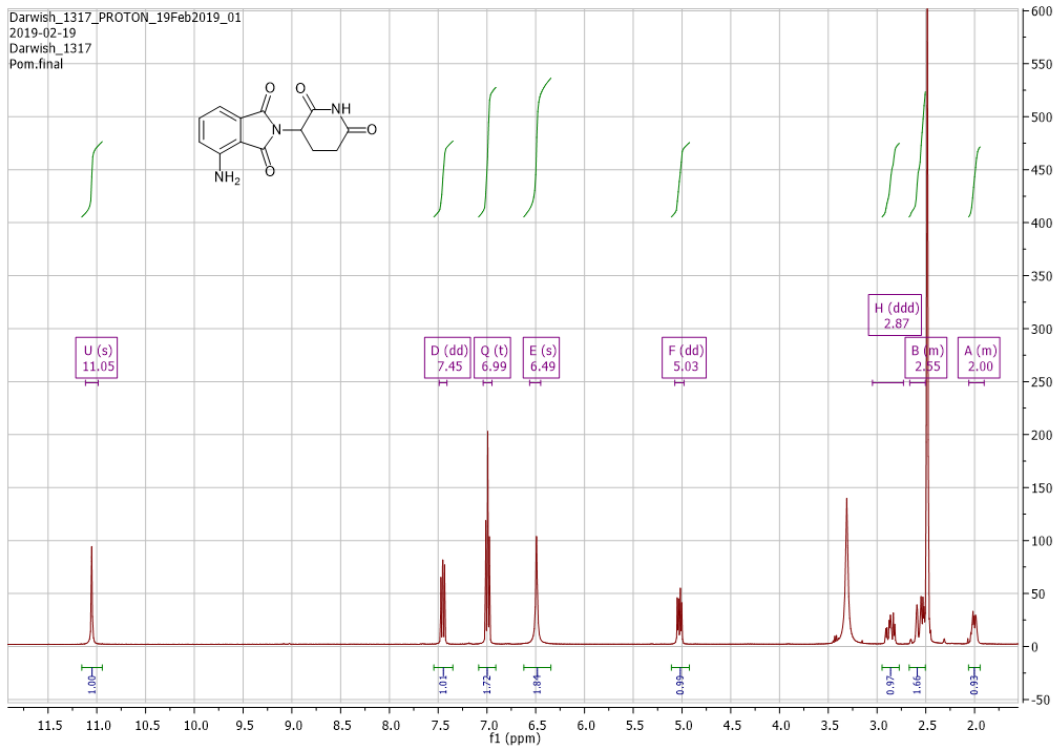

^{13}C NMR (126 MHz, DMSO-d6) δ 173.24, 170.53, 169.01, 167.81, 147.17, 135.91, 132.44, 122.15, 111.43, 108.99, 49.03, 31.42, 22.60.

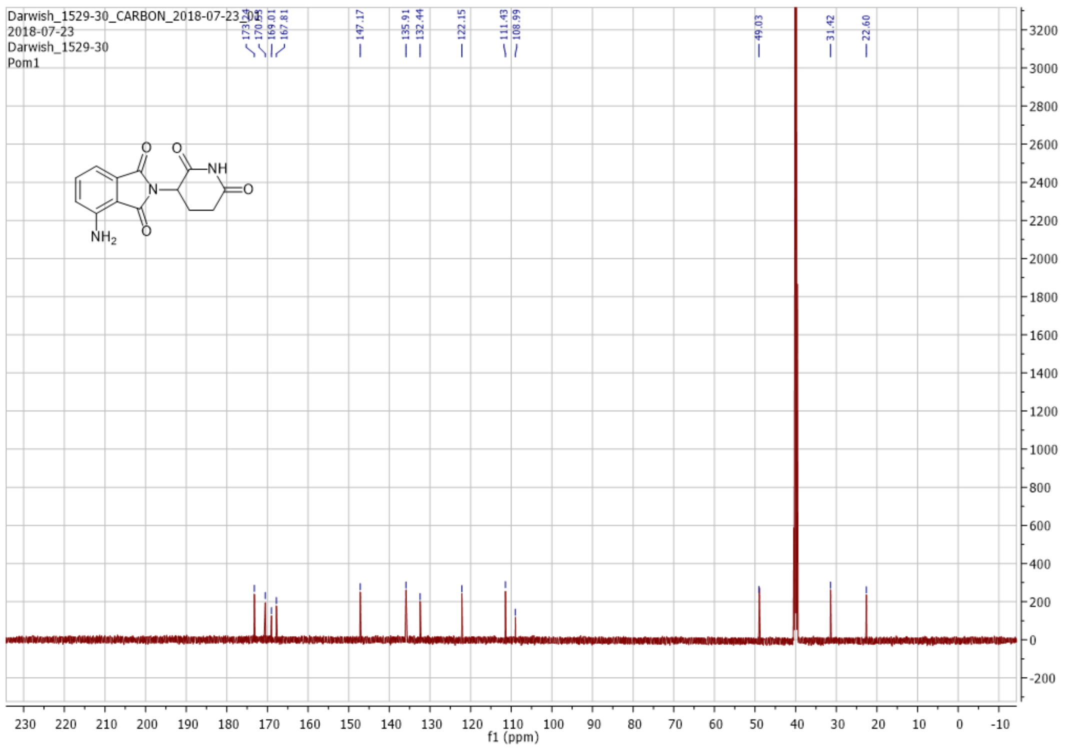

4-Fluorothalidomide (39)

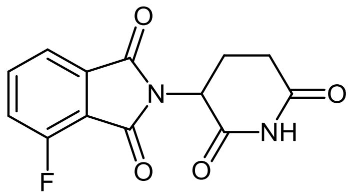

MS m/z: 275.42 [M-H]⁻

^{1}H NMR (400 MHz, DMSO-d6) δ 11.12 (s, J = 43.7 Hz, 1H), 7.93 (ddd, J = 8.4, 7.4, 4.6 Hz, 1H), 7.79 – 7.68 (m, 2H), 5.14 (dd, J = 12.8, 5.4 Hz, 1H), 2.88 (ddd, J = 17.1, 13.9, 5.5 Hz, 1H), 2.64 – 2.49 (m, 2H), 2.10 – 2.00 (m, 1H).

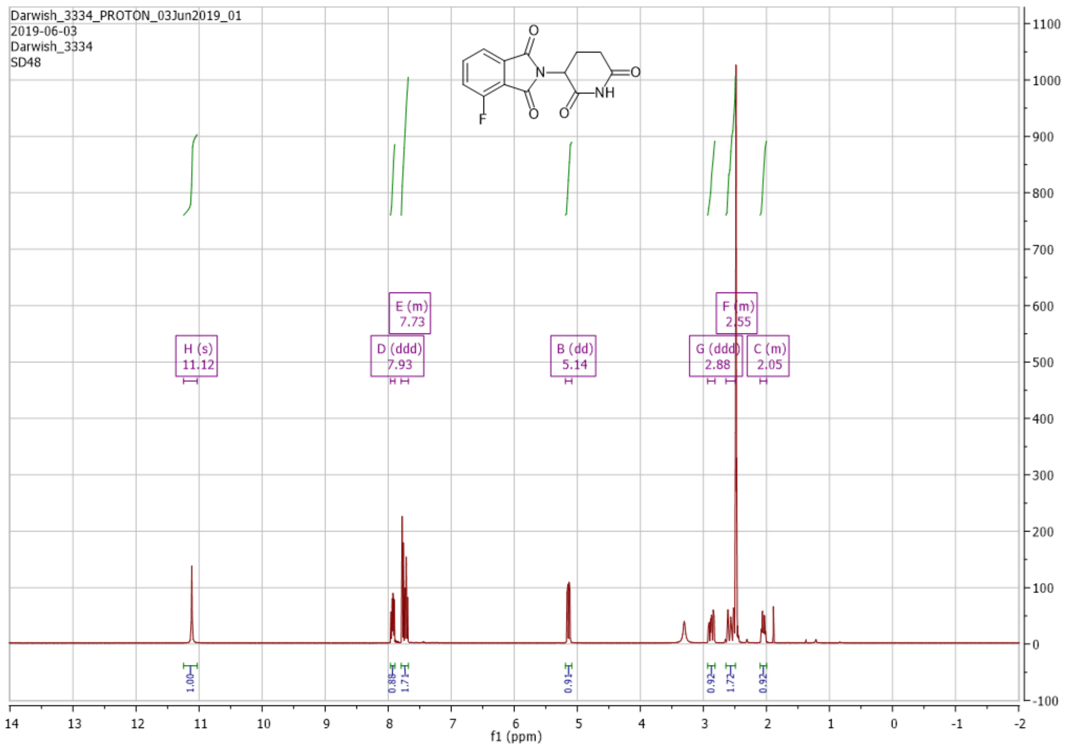

VHL ligand (40)

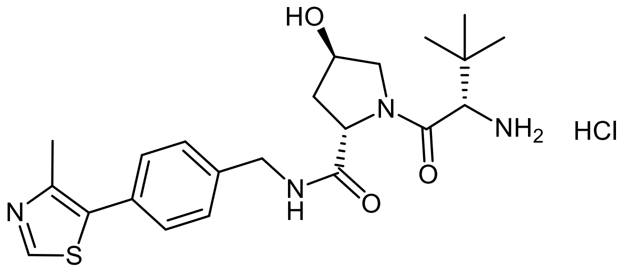

MS m/z: 431.05 [M+H]⁺

^{1}H NMR (400 MHz, DMSO-d6) δ 9.06 (s, 1H), 8.73 (t, J = 5.9 Hz, 1H), 8.20 – 8.13 (m, 2H), 7.39 – 7.38 (m, 2H), 6.41 (br s, 3H), 4.53 (t, J = 8.3 Hz, 1H), 4.41 (dd, J = 15.8, 6.4 Hz, 1H), 4.37 – 4.32 (m, 1H), 4.23 (dd, J =

15.8, 5.5 Hz, 1H), 3.93 – 3.82 (m, 1H), 3.78 (d, J = 11.0 Hz, 1H), 3.55 (d, J = 3.6 Hz, 1H), 3.53 (d, J = 3.7 Hz, 1H), 2.43 (s, J = 4.6 Hz, 3H), 2.17 – 2.03 (m, 1H), 1.94 – 1.81 (m, 1H), 1.01 (s, 9H).

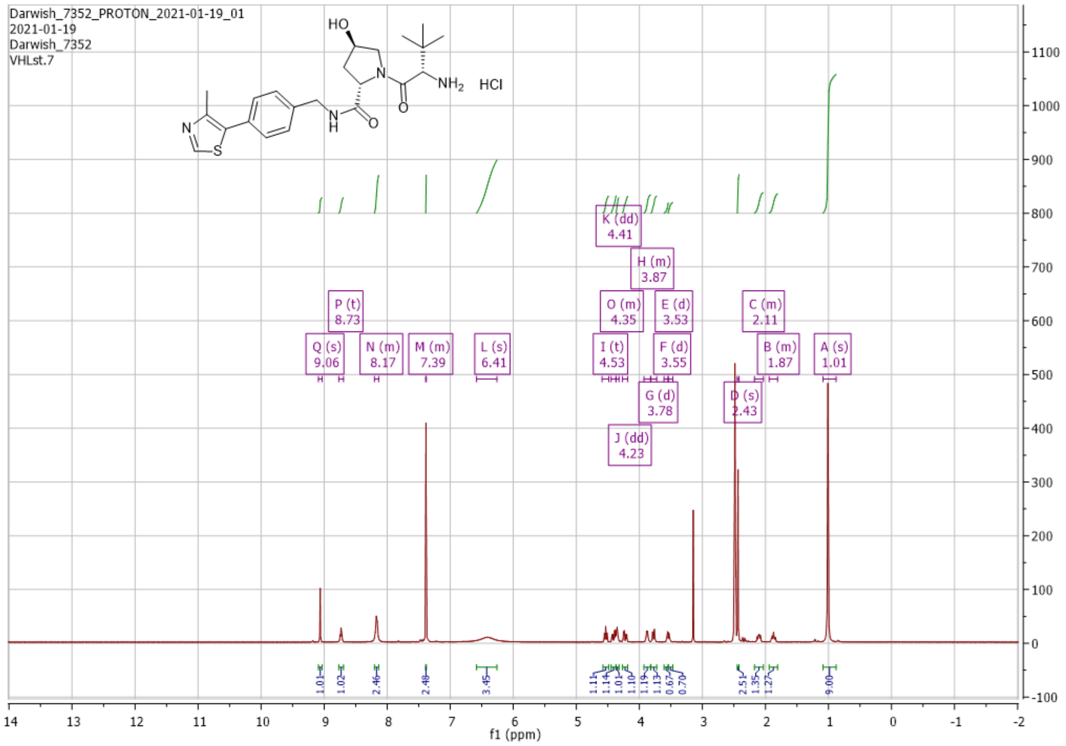

^{13}C NMR (101 MHz, DMSO-d6) δ 171.95, 167.18, 152.18, 147.74, 139.95, 131.80, 129.95, 129.13, 127.87, 69.42, 59.44, 58.46, 56.99, 49.02, 38.54, 34.86, 26.45, 16.20.

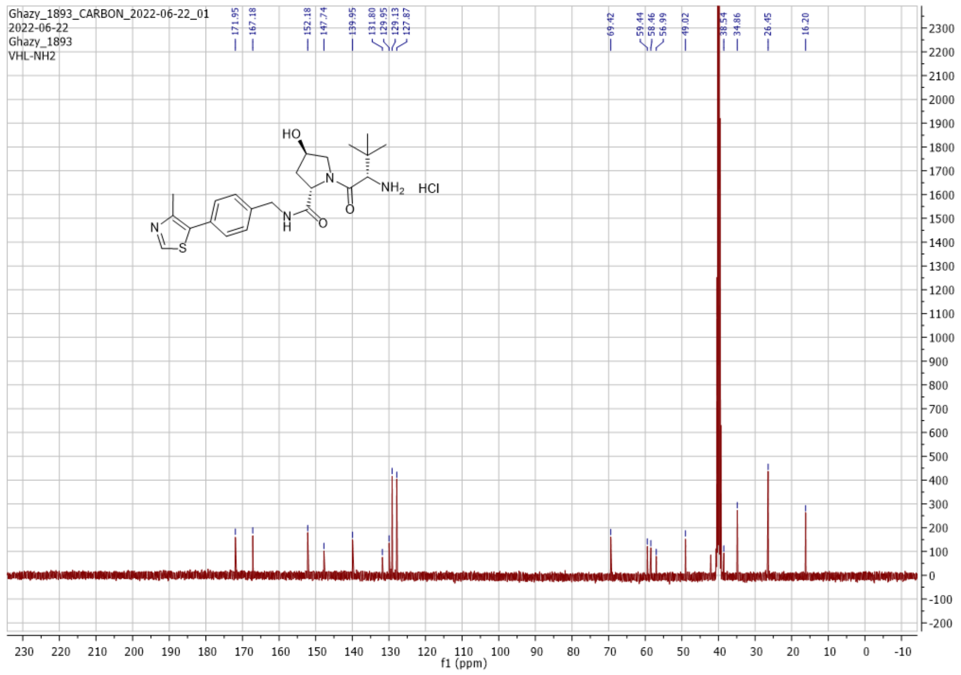

The characterization data of inhibitors 2a-g are reported in the supplementary information part of the following references [16,50].

Methyl 4-methoxy-3-((4-methoxybenzyl)amino)benzoate (28)

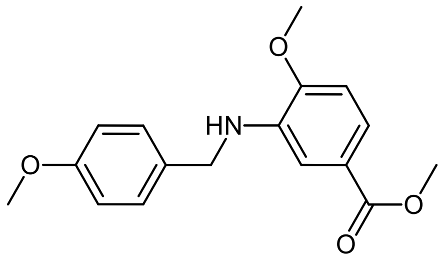

MS m/z: 324.62 $[M+Na]^+$

^{1}H NMR (400 MHz, DMSO-d6) δ 7.24 (d, J = 8.4 Hz, 3H), 6.96 (d, J = 2.0 Hz, 1H), 6.86 (d, J = 8.8 Hz, 3H), 5.66 (t, J = 6.1 Hz, 1H), 4.26 (d, J = 6.1 Hz, 2H), 3.86 (s, 3H), 3.71 (s, 3H), 3.70 (s, 3H).

N-hydroxy-4-methoxy-3-((4-methoxybenzyl)amino)benzamide (2h)

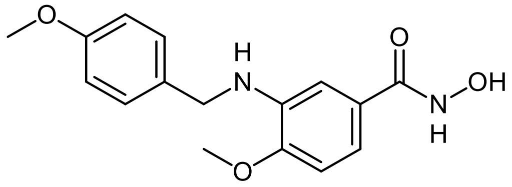

MS m/z: 301.25 $[M-H]^-$

^{1}H NMR (400 MHz, DMSO-d6) δ 10.85 (s, 1H), 8.72 (br s, 1H), 7.24 (d, J = 8.7 Hz, 2H), 6.97 (d, J = 8.2 Hz, 1H), 6.88 – 6.83 (m, 3H), 6.81 (d, J = 8.3 Hz, 1H), 5.49 (m, 1H), 4.26 (s, 2H), 3.82 (s, 3H), 3.70 (s, 3H).

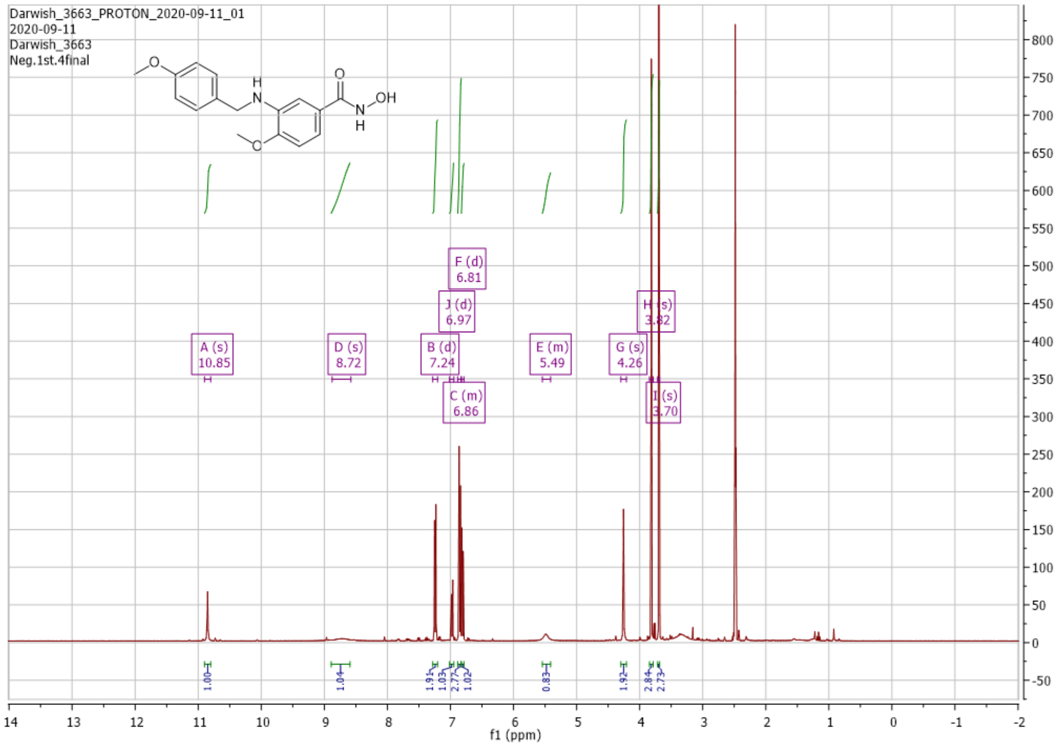

^{13}C NMR (101 MHz, DMSO-d6) δ 158.55, 149.08, 137.80, 132.25, 132.18, 128.73, 125.72, 115.43, 114.16, 109.34, 108.40, 56.00, 55.43, 45.99.

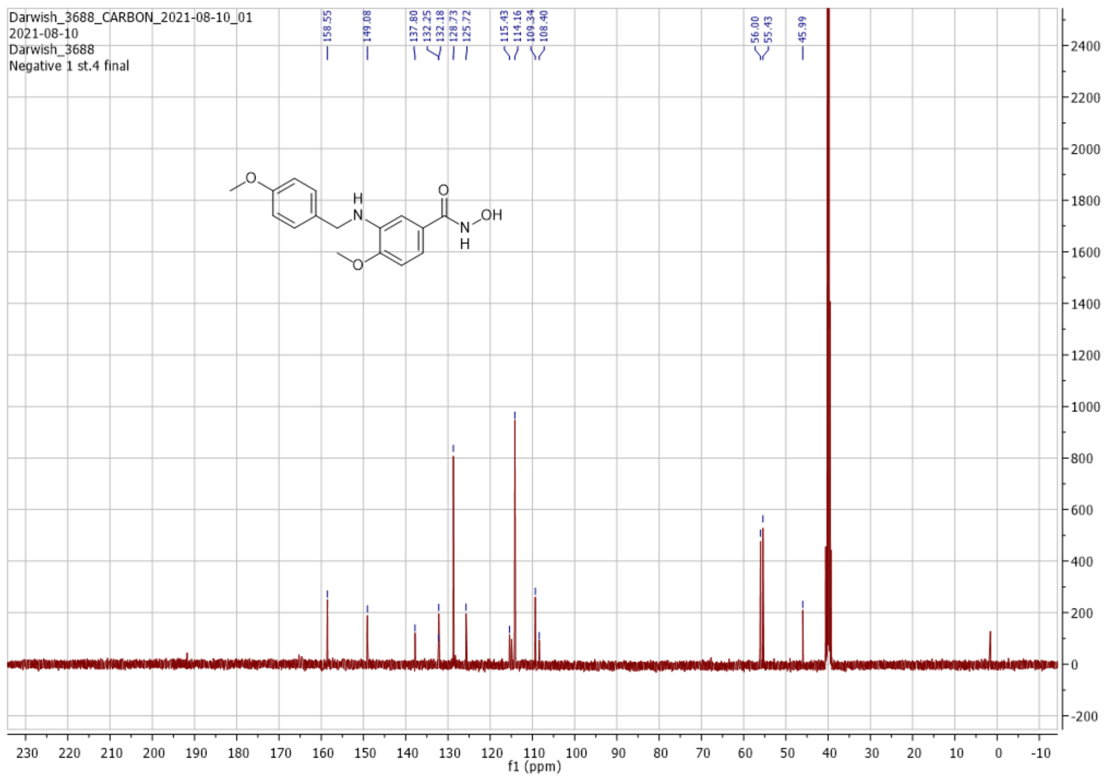

HRMS m/z: 303.134 [M+H]$^+$, calculated: $C_{16}H_{19}N_2O_4^+$: 303.134483

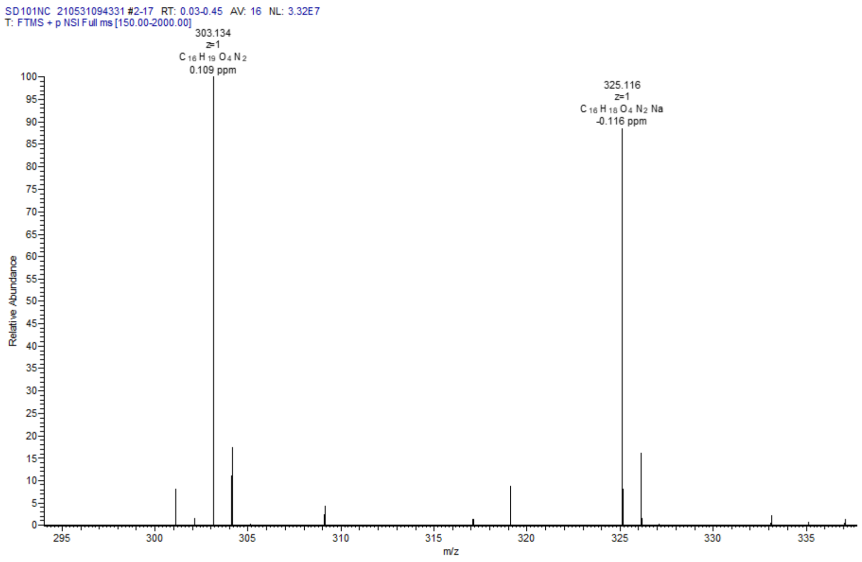

HPLC: (M2) rt. 4.2 min (purity >99%)

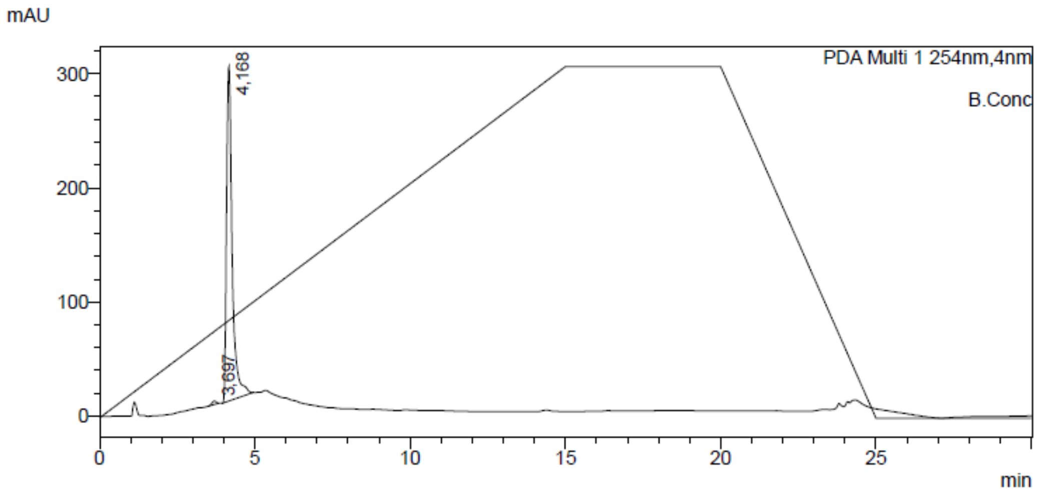

PDA Ch1 254nm

Peak#	Ret. Time	Area	Height	Area%
1	3,697	29547	3009	0,792
2	4,168	3700857	292752	99,208
Total		3730404	295761	100,000

3-({4-[2-(Benzyloxy)-2-oxoethoxy]benzyl}amino)-4-chlorobenzoic acid (31)

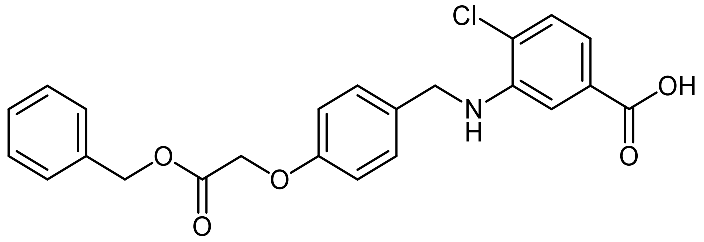

MS m/z: 424.26 [M-H]$^-$

^{1}H NMR (400 MHz, DMSO-d6) δ 12.78 (s, 1H), 7.41 – 7.27 (m, 6H), 7.23 (d, J = 8.6 Hz, 2H), 7.14 – 7.06 (m, 2H), 6.87 (d, J = 8.7 Hz, 2H), 6.27 (t, J = 6.0 Hz, 1H), 5.17 (s, 2H), 4.80 (s, 2H), 4.36 (d, J = 5.9 Hz, 2H).

Benzyl 2-[4-({[2-chloro-5-(hydroxycarbamoyl)phenyl]amino}methyl)phenoxy]acetate (2i)

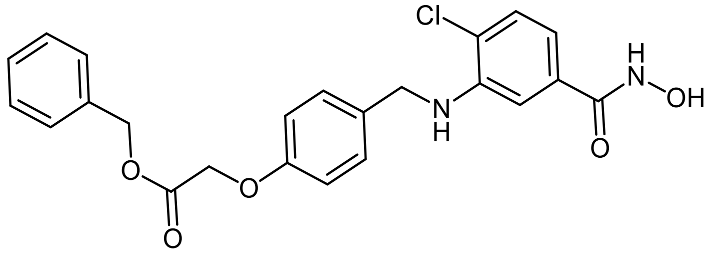

MS m/z: 439.20 [M-H]$^-$

^{1}H NMR (400 MHz, DMSO-d6) δ 11.03 (s, 1H), 8.97 (s, 1H), 7.36 – 7.20 (m, 8H), 6.97 – 6.84 (m, 4H), 6.16 (t, J = 6.1 Hz, 1H), 5.17 (s, 2H), 4.80 (s, 2H), 4.36 (d, J = 6.0 Hz, 2H).

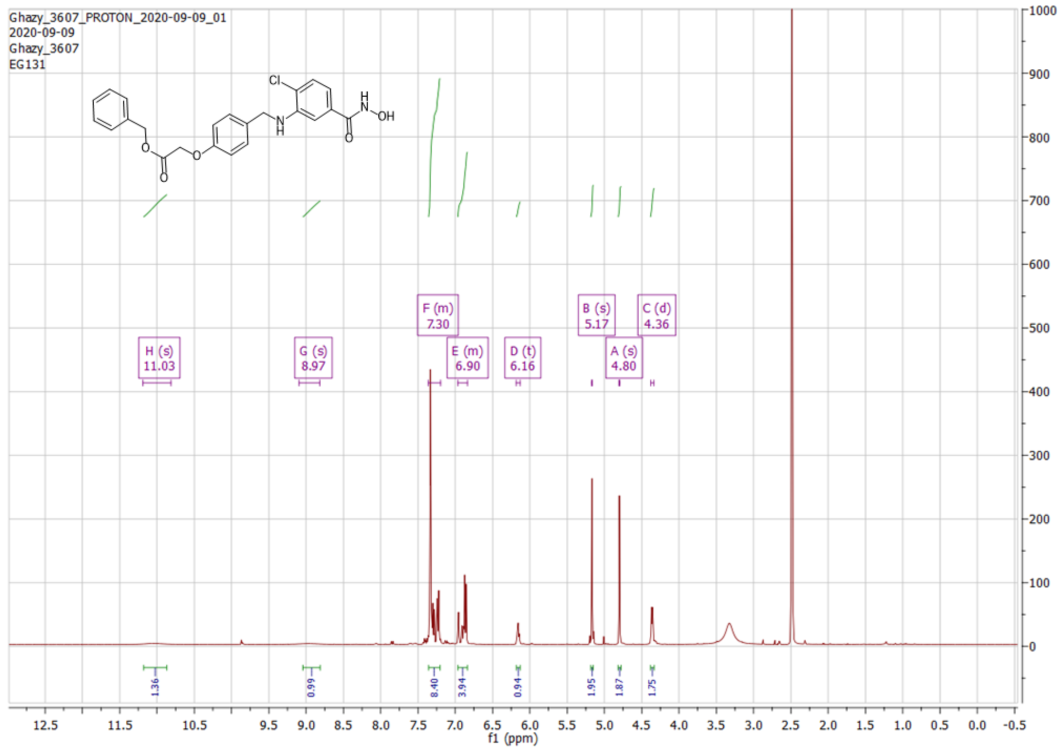

HRMS m/z: 441.1210 [M+H]+, calculated $C_{23}H_{22}ClN_2O_5^+$: 441.1217

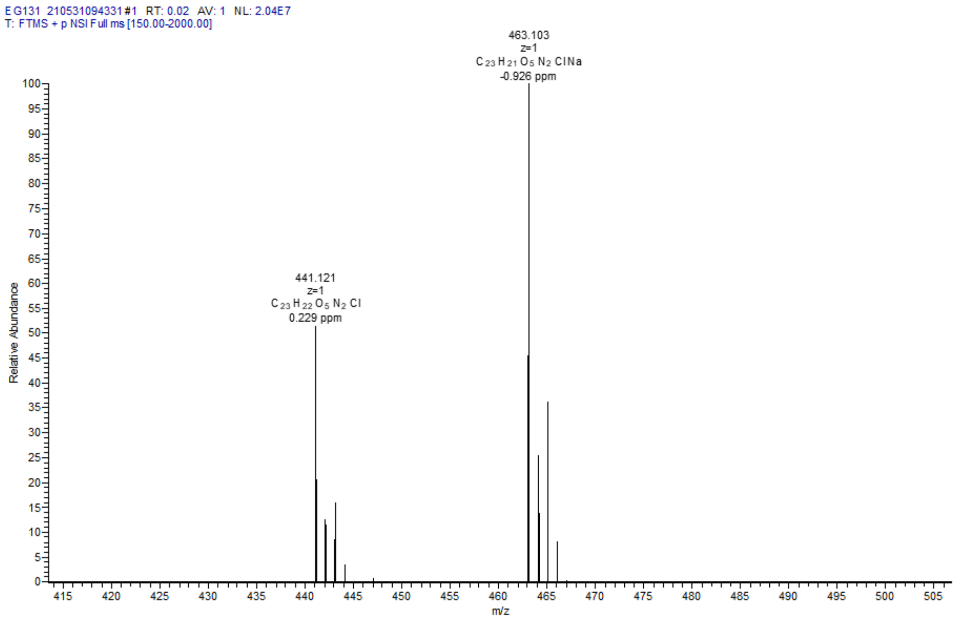

HPLC: (M2) rt. 9.9 min (purity 98%)

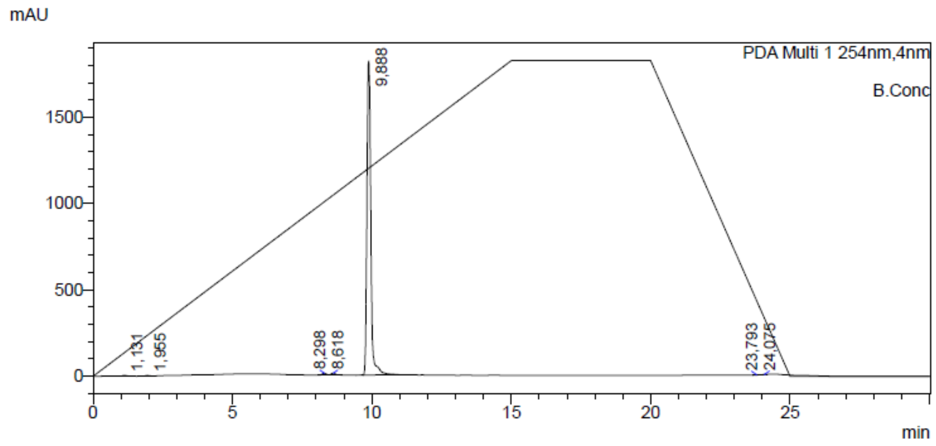

PDA Ch1 254nm

Peak#	Ret. Time	Area	Height	Area%
1	1,131	29448	3668	0,163
2	1,955	21498	2223	0,119
3	8,298	111914	10628	0,619
4	8,618	118727	10984	0,656
5	9,888	17782029	1820671	98,287
6	23,793	18379	2344	0,102
7	24,075	9976	1722	0,055
Total		18091971	1852242	100,000

Methyl 3-(4-((tert-butoxycarbonyl)amino)benzamido)-4-methoxybenzoate (7)

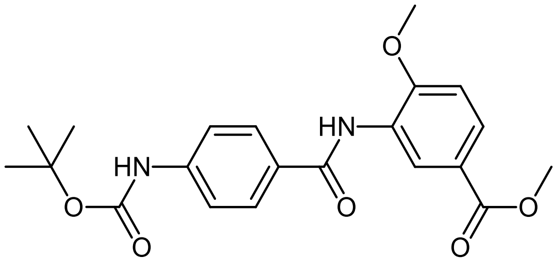

^{1}H NMR (400 MHz, DMSO-d6) δ 9.67 (s, 1H), 9.34 (s, 1H), 8.43 (d, J = 2.2 Hz, 1H), 7.88 (d, J = 8.8 Hz, 2H), 7.79 (dd, J = 8.6, 2.2 Hz, 1H), 7.57 (d, J = 8.8 Hz, 2H), 7.19 (d, J = 8.7 Hz, 1H), 3.91 (s, 3H), 3.82 (s, 3H), 1.48 (s, J = 7.2 Hz, 9H).

3-(4-Aminobenzamido)-4-methoxy-N-((tetrahydro-2H-pyran-2-yl)oxy)benzamide (9)

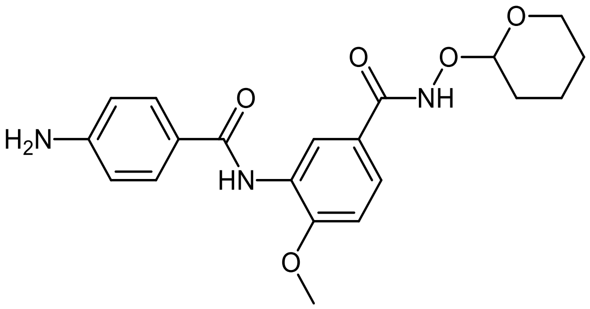

MS m/z: 384.23 $[M-H]^-$

^{1}H NMR (400 MHz, DMSO-d6) δ 11.48 (s, 1H), 8.98 (s, 1H), 8.26 (d, J = 2.1 Hz, 1H), 7.68 (d, J = 8.6 Hz, 2H), 7.57 (dd, J = 8.6, 2.1 Hz, 1H), 7.12 (d, J = 8.7 Hz, 1H), 6.59 (d, J = 8.6 Hz, 2H), 5.76 (s, 2H), 5.03 – 4.91 (m, 1H), 4.11 – 4.00 (m, 1H), 3.88 (s, 3H), 3.56 – 3.47 (m, 1H), 1.78 – 1.65 (m, 3H), 1.60 – 1.47 (m, 3H).

8-((4-((2-Methoxy-5-(((tetrahydro-2H-pyran-2-yl)oxy)carbamoyl)phenyl)carbamoyl)phenyl)amino)-8-oxooctanoic acid (10)

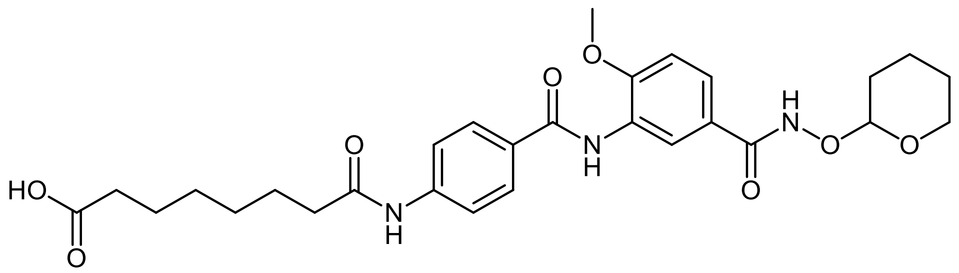

MS m/z: 540.35 [M-H]$^-$

^{1}H NMR (400 MHz, DMSO-d6) δ 11.53 (s, 2H), 10.20 (s, 1H), 9.42 (s, 1H), 8.17 (d, J = 2.2 Hz, 1H), 7.91 (d, J = 8.8 Hz, 2H), 7.72 (d, J = 8.8 Hz, 2H), 7.63 (dd, J = 8.6, 2.2 Hz, 1H), 7.15 (d, J = 8.7 Hz, 1H), 5.00 – 4.95 (m, 1H), 4.12 – 4.00 (m, 2H), 3.87 (s, 3H), 2.33 (t, J = 7.5 Hz, 2H), 2.19 (t, J = 7.4 Hz, 2H), 1.76 – 1.66 (m, 3H), 1.63 – 1.43 (m, 7H), 1.35 – 1.24 (m, 4H).

3-(4-(((Tert-butoxycarbonyl)amino)methyl)benzamido)-4-methoxybenzoic acid (13)

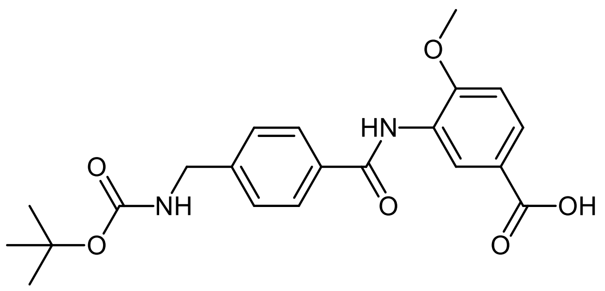

^{1}H NMR (500 MHz, DMSO-d6) δ 12.55 (s, 1H), 9.33 (s, 1H), 8.24 (s, 1H), 7.77 (d, J = 7.9 Hz, 2H), 7.65 (dd, J = 8.6, 1.9 Hz, 1H), 7.34 (t, J = 5.8 Hz, 1H), 7.23 (d, J = 8.0 Hz, 2H), 7.04 (d, J = 8.7 Hz, 1H), 4.05 (d, J = 5.9 Hz, 2H), 3.76 (s, 3H), 1.26 (s, 9H).

3-(4-(Aminomethyl)benzamido)-N-(benzyloxy)-4-methoxybenzamide (14)

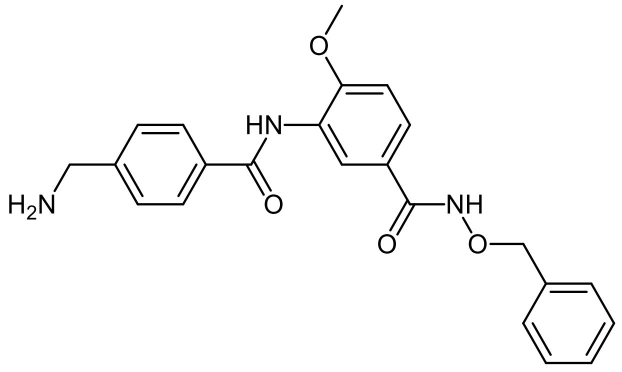

MS m/z: 406.06 [M+H]$^+$

^{1}H NMR (400 MHz, DMSO-d6) δ 9.48 (s, 1H), 8.18 (d, J = 2.2 Hz, 1H), 7.92 (d, J = 8.3 Hz, 2H), 7.61 (dd, J = 8.6, 2.2 Hz, 1H), 7.53 – 7.30 (m, 6H), 7.26 – 7.21 (m, 1H), 7.18 – 7.10 (m, 2H), 4.91 (s, 2H), 3.87 (s, 3H), 3.83 (s, 2H).

4-Methoxy-3-((4-(prop-2-yn-1-yloxy)benzyl)amino)benzoic acid (17a)

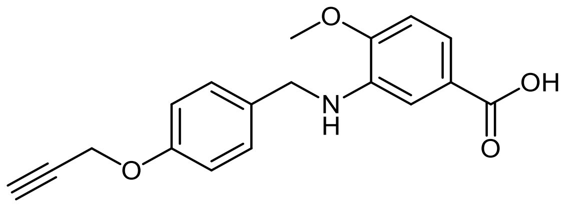

^{1}H NMR (400 MHz, DMSO-d6) δ 12.26 (s, 1H), 7.25 (d, J = 8.6 Hz, 2H), 7.20 (dd, J = 8.3, 1.9 Hz, 1H), 6.96 (d, J = 1.9 Hz, 1H), 6.91 (d, J = 8.6 Hz, 2H), 6.86 (d, J = 8.4 Hz, 1H), 5.70 – 5.53 (m, 1H), 4.73 (d, J = 2.4 Hz, 2H), 4.26 (s, 2H), 3.85 (s, 3H), 3.50 (t, J = 2.4 Hz, 1H).

2-(4-{[(2-Chloro-5-{[(tetrahydro-2H-pyran-2-yl)oxy]carbamoyl}phenyl)amino]methyl}phenoxy)acetic acid (20)

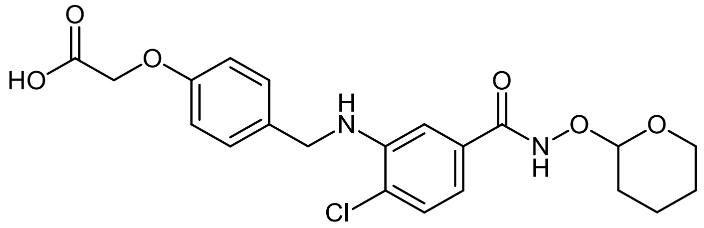

MS m/z: 433.30 [M-H]$^-$

^{1}H NMR (400 MHz, DMSO-d6) δ 12.91 (s, 1H), 11.49 (s, 1H), 7.31 (d, J = 8.2 Hz, 1H), 7.24 (d, J = 8.8 Hz, 2H), 6.98 (d, J = 1.9 Hz, 1H), 6.92 (dd, J = 8.2, 2.0 Hz, 1H), 6.86 – 6.82 (m, 2H), 6.16 (t, J = 6.1 Hz, 1H), 4.91 (s, 1H), 4.60 (s, 2H), 4.34 (d, J = 6.0 Hz, 2H), 4.06 – 3.96 (m, 1H), 3.52 – 3.42 (m, 1H), 1.71 – 1.45 (m, 6H).

Benzyl 4-chloro-3-[4-(2-methoxy-2-oxoethoxy)benzamido]benzoate (23)

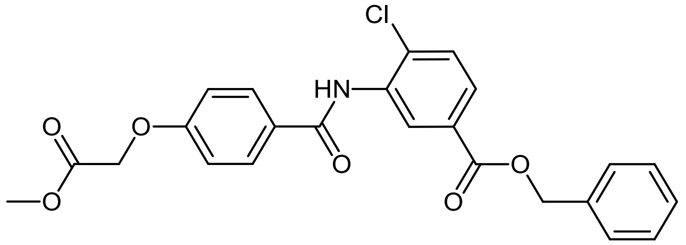

MS m/z: 476.36 [M+Na]$^+$

^{1}H NMR (400 MHz, DMSO-D6) δ 10.06 (s, 1H), 8.19 (d, J = 2.1 Hz, 1H), 7.98 – 7.93 (m, 2H), 7.86 (dd, J = 8.4, 2.1 Hz, 1H), 7.71 (d, J = 8.4 Hz, 1H), 7.50 – 7.32 (m, 5H), 7.10 – 7.04 (m, 2H), 5.37 (s, 2H), 4.92 (s, 2H), 3.71 (s, 3H).

2-[4-({5-[(Benzyloxy)carbamoyl]-2-chlorophenylcarbamoyl)phenoxy]acetic acid (26)

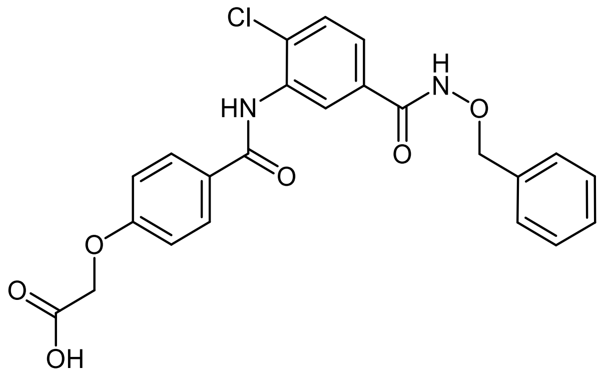

MS m/z: 453.17 [M-H]-

^{1}H NMR (400 MHz, DMSO-d6) δ 12.98 (s, 1H), 11.87 (s, 1H), 10.01 (s, 1H), 7.97 – 7.92 (m, 3H), 7.67 – 7.60 (m, 2H), 7.47 – 7.33 (m, 5H), 7.07 – 7.01 (m, 2H), 4.92 (s, 2H), 4.78 (s, 2H).

4-[(3-{2-[2-(3-Aminopropoxy)ethoxy]ethoxy}propyl)amino]-2-(2,6-dioxopiperidin-3-yl)isoindoline-1,3-dione (53)

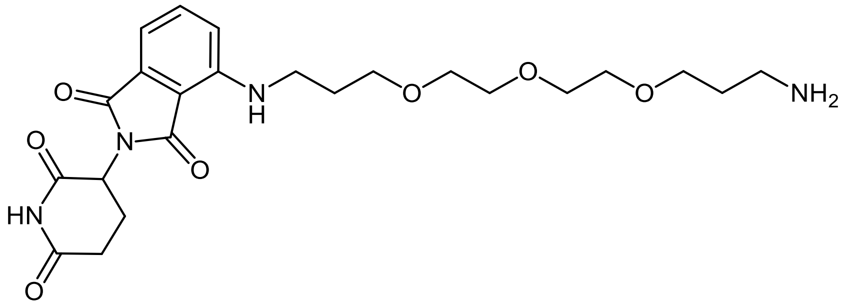

1-N-Boc-1,13-diamino-4,7,10-trioxatridecane (52) was prepared as previously reported [70], and then used to prepare the title compound according to an already published method [71]. Characterization data were in accordance with the reported values.

4-[(5-Aminopentyl)amino]-2-(2,6-dioxopiperidin-3-yl)isoindoline-1,3-dione (56)

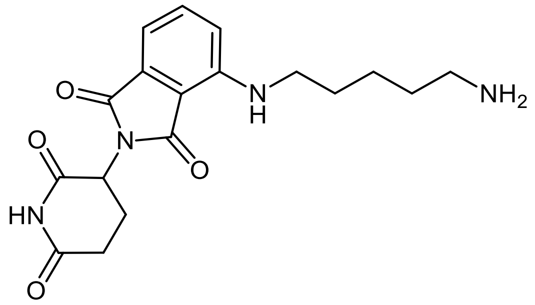

Tert-butyl (5-aminopentyl)carbamate (55) was similarly prepared using the previously reported method [70], and then used to prepare the title compound according to an already published method [71]. Characterization data were in accordance with the reported values.

2-(Adamantan-1-yl)-N-(5-aminopentyl)acetamide (57)

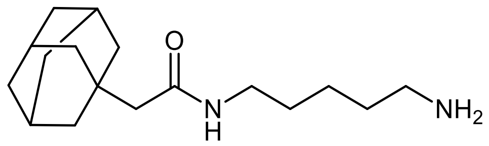

Tert-butyl (5-aminopentyl)carbamate (55) was reacted with commercially available 1-adamantaneacetic acid (45) using method IIIA, followed by removal of the Boc protecting group using method (VI) to afford the title compound as TFA salt.

Yield: 40%

MS m/z: 279.20 $[M+H]^+$

^{1}H NMR (400 MHz, CDCl3) δ 7.94 (s, 1H), 6.56 – 6.62 (m, 3H), 3.21 (t, J = 10.7 Hz, 2H), 2.88 – 3.03 (m, 2H), 2.84 (s, 1H), 1.89 – 2.03 (m, 5H), 1.32 – 1.80 (m, 17H).

(2S,4R)-1-[(S)-2-(6-aminohexanamido)-3,3-dimethylbutanoyl]-4-hydroxy-*N*-[4-(4-methylthiazol-5-yl)benzyl]pyrrolidine-2-carboxamide (59)

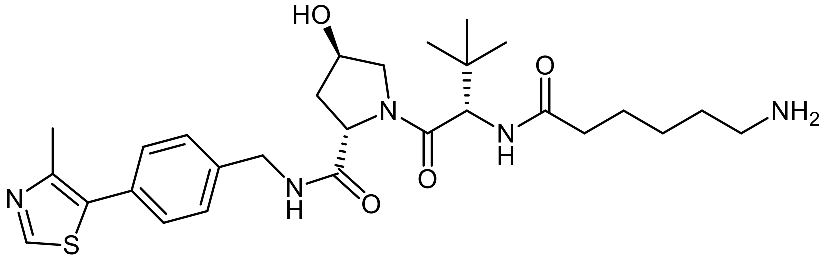

6-[(Tert-butoxycarbonyl)amino]hexanoic acid (58) was prepared as previously reported [72], and then used to prepare the title compound according to an already published method [73]. Characterization data were in accordance with the reported values.

S6. Non- Enzymatic stability data for PROTACs CRBN_1b and CRBN_1e

	0h - %	6h - %	12h - %	24h - %
**CRBN_1b**	100.0	100.0	100.0	91.6
**CRBN_1e**	100.0	91.6	82.8	65.5

CRBN_1b

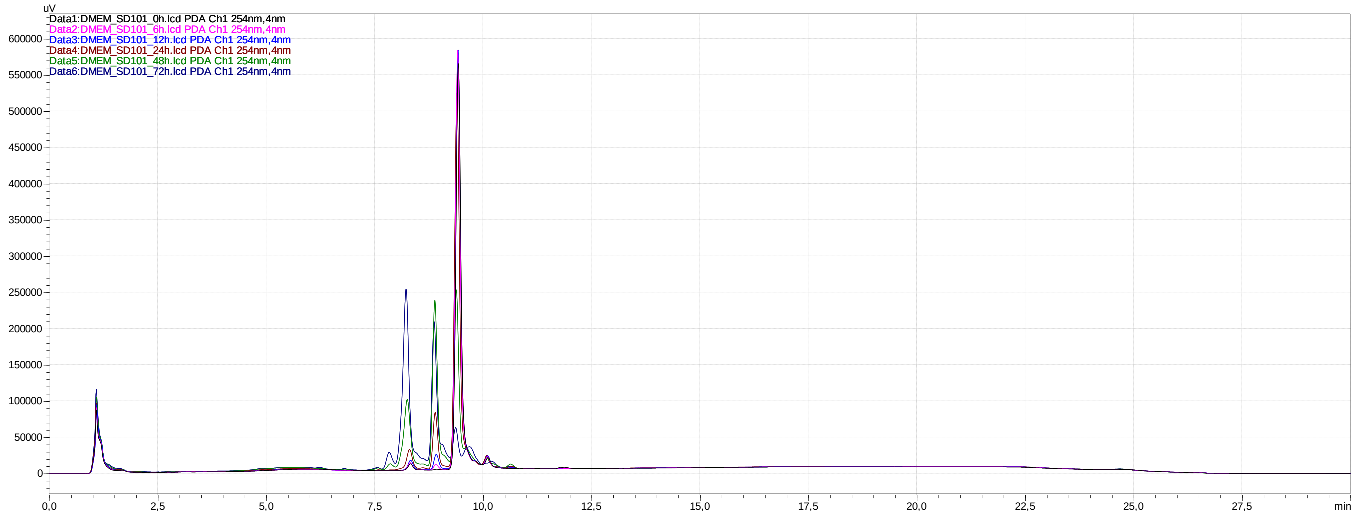

CRBN_1e

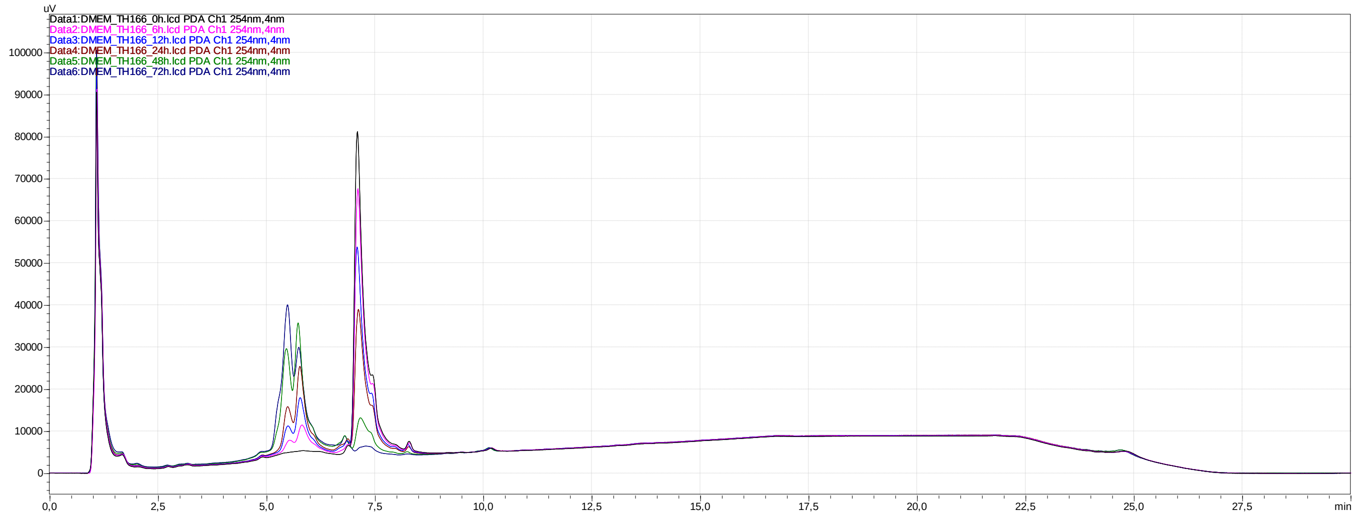

S7. HDAC8 degradation by CRBN_1e in combination with the de novo synthesis inhibitor cycloheximide (CHX)

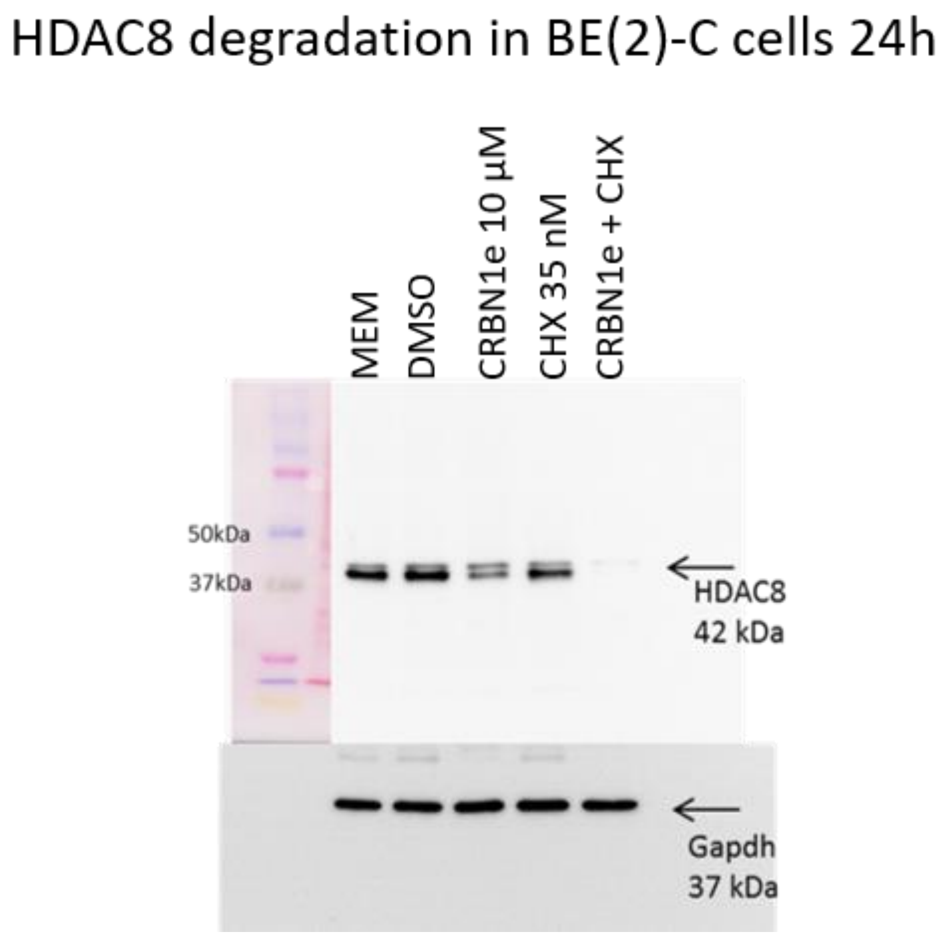

S8. HDAC6 degradation and tubulin hyperacetylation by PROTACs and control compounds

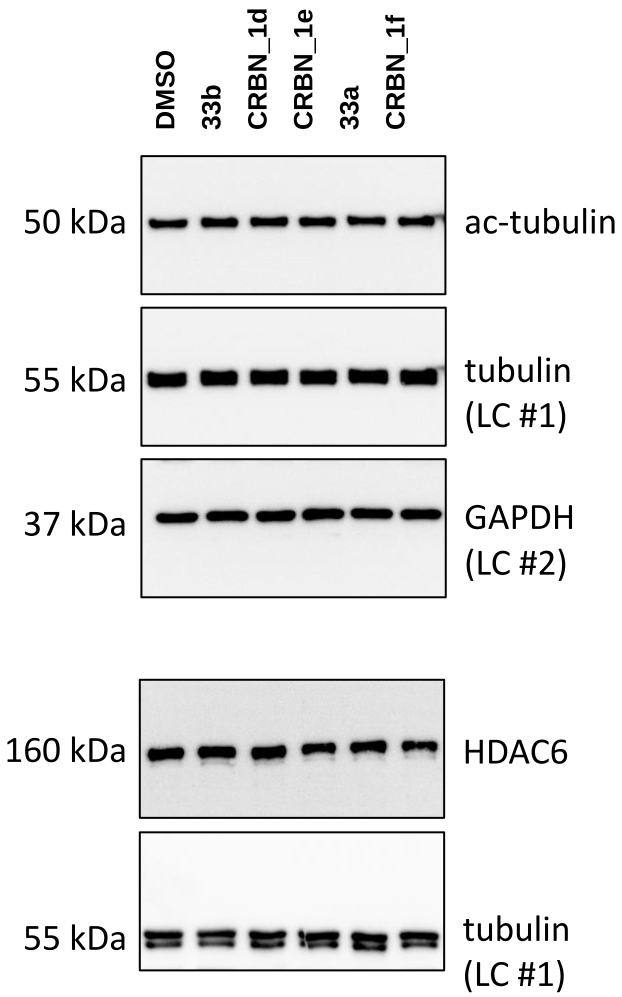

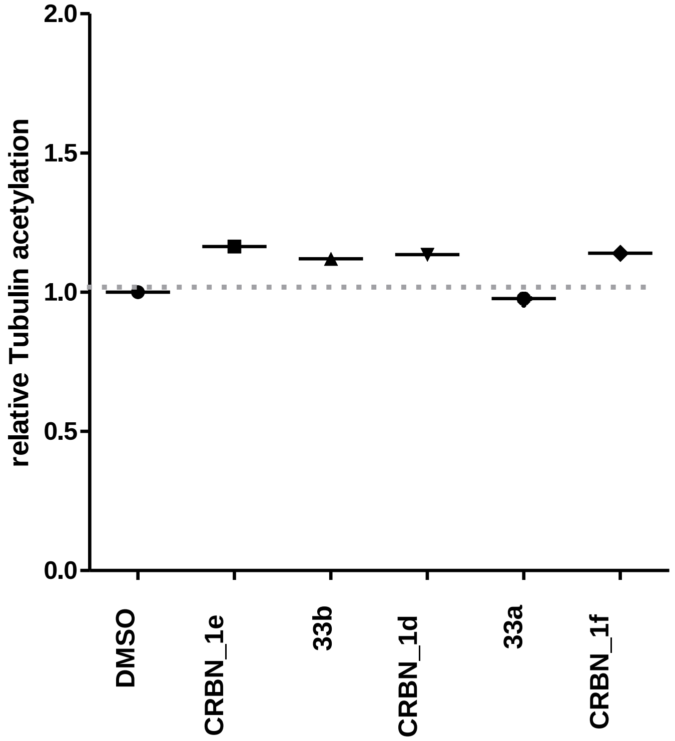

Left: SK-N-BE(2)-C neuroblastoma cells were treated for 6 h with indicated PROTACs (10 μM each). Total HDAC6, acetyl-tubulin and total tubulin (LC #1: loading control #1) expression levels, were assessed by western blot. Total GAPDH protein levels served as a second loading control (LC #2). Right: presentation of quantified acetyl-tubulin levels, normalized to the DMSO control
